# Cobalt-catalyzed *peri*-selective alkoxylation of 1-naphthylamine derivatives

**DOI:** 10.3762/bjoc.14.183

**Published:** 2018-08-09

**Authors:** Jiao-Na Han, Cong Du, Xinju Zhu, Zheng-Long Wang, Yue Zhu, Zhao-Yang Chu, Jun-Long Niu, Mao-Ping Song

**Affiliations:** 1 College of Chemistry and Molecular Engineering, Zhengzhou University, Zhengzhou 450001, People’s Republic of China

**Keywords:** alkoxylation, C–H activation, cobalt catalysis, 1-naphthylamines, secondary alcohols

## Abstract

A cobalt-catalyzed C(sp^2^)–H alkoxylation of 1-naphthylamine derivatives has been disclosed, which represents an efficient approach to synthesize aryl ethers with broad functional group tolerance. It is noteworthy that secondary alcohols, such as hexafluoroisopropanol, isopropanol, isobutanol, and isopentanol, were well tolerated under the current catalytic system. Moreover, a series of biologically relevant fluorine-aryl ethers were easily obtained under mild reaction conditions after the removal of the directing group.

## Introduction

Aryl ethers are common structural units present in many natural products, functional materials, and pharmaceuticals [1]. Consequently, a variety of strategies have emerged to access them, including Pd-catalyzed and Cu-catalyzed coupling reactions (Buchwald–Harting couplings and Ullmann reactions) [2–4]. However, these classic methods always possess some limitations such as preactivated starting materials, poor regioselectivities, and tedious steps [5]. Therefore, it is desirable to develop an effective strategy to achieve this transformation [6–7].

**Figure 1:**
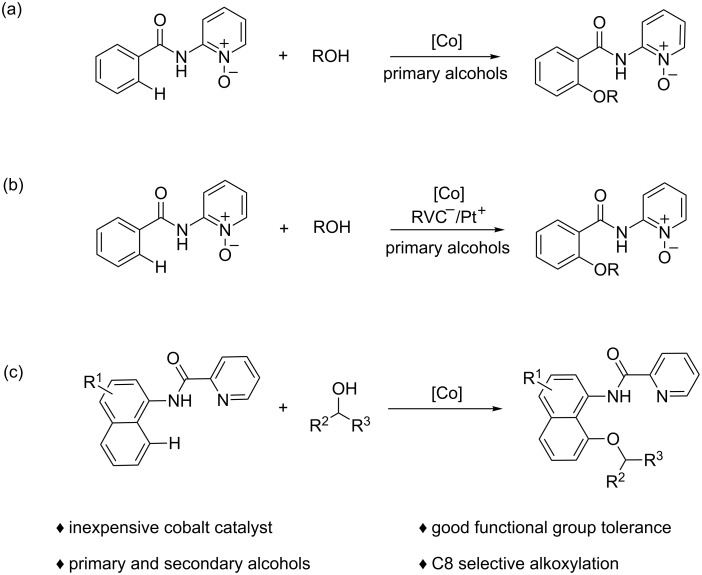
Strategies for cobalt-catalyzed alkoxylation.

Over the past few decades, transition-metal-catalyzed C–H activation to form C–C or C–heteroatom bonds has attracted more attention [8–13]. In particular, the formation of C–O bonds is widely used in the syntheses of pharmaceuticals and functional materials [14–17]. The direct hydroxylation [18–19] and acetoxylation [20–22] have been developed rapidly in recent years. By contrast, alkoxylation or phenoxylation confronts great challenges because alkanols or phenols are easily converted into the corresponding aldehydes, ketones, or carboxylic acids [7,23–25]. Recently, Gooßen [26–27], Sanford [28], Song, [29–30] and others [31–42] have successfully reported alkoxylation reactions with the auxiliary of directing groups. However, the transition-metal-catalyzed C–H alkoxylation is still largely limited to palladium- [28,33–40] or copper- [26–27,29,41–42] catalyzed systems. Recently, the inexpensive cobalt catalysts have received significant attention because of their unique and versatile activities in the C–H functionalizations [43–51]. In 2015, the cobalt-catalyzed alkoxylation of aromatic (and olefinic) carboxamides with primary alcohols was first reported by the Niu and Song group (Figure 1a) [30]. Successively, Ackermann realized the electrochemical cobalt-catalyzed alkoxylation via a similar process (Figure 1b) [32]. However, cobalt-catalyzed directed coupling of arenes with secondary alcohols has not been reported so far. Herein, we explored a simple and facile protocol for cobalt-catalyzed picolinamide-directed alkoxylation of 1-naphthylamine derivatives with alcohols (Figure 1c).

## Results and Discussion

Initially, *N*-(naphthalen-1-yl)picolinamide (**1a**) and hexafluoroisopropanol (HFIP, **2a**) were chosen as the model substrates to optimize the alkoxylation reaction (Table 1, see more in Tables S1–S5 in Supporting Information File 1). To our delight, the desired product **3aa** was obtained in 66% yield when using Co(OAc)_2_·4H_2_O as catalyst and Ag_2_CO_3_ as oxidant (Table 1, entry 1). Other cobalt salts such as CoF_3_ and CoF_2_ were also employed as metal catalysts for C8 alkoxylation of **1a**, and CoF_2_ was proved to be the optimal catalyst, affording **3aa** in 71% yield (Table 1, entries 2 and 3). Subsequently, various bases such as Na_2_CO_3_, K_2_CO_3_, and Cs_2_CO_3_ were screened (Table 1, entries 4–6), which indicated that Cs_2_CO_3_ was most effective and the alkoxylated product **3aa** could be isolated in 82% yield. Next, the effect of oxidants on the reactivity was investigated, and Ag_2_CO_3_ showed a superior result compared with alternative oxidants (Table 1, entries 6–8). Moreover, DCE and HFIP as co-solvents demonstrated higher reactivity, resulting in a slightly increased yield in 84% (Table 1, entries 9–11). Finally, variation of the reaction temperature did not promote the reaction efficiency.

**Table 1:** Optimization of the reaction conditions.^a^

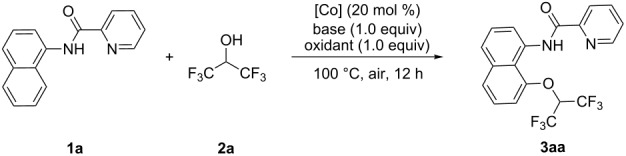				

entry	catalyst	base	oxidant	yield (%)

1	Co(OAc)_2_·4H_2_O	*t*-AmONa	Ag_2_CO_3_	66
2	CoF_3_	*t*-AmONa	Ag_2_CO_3_	60
3	CoF_2_	*t*-AmONa	Ag_2_CO_3_	71
4	CoF_2_	Na_2_CO_3_	Ag_2_CO_3_	58
5	CoF_2_	K_2_CO_3_	Ag_2_CO_3_	67
6	CoF_2_	Cs_2_CO_3_	Ag_2_CO_3_	82
7	CoF_2_	Cs_2_CO_3_	AgNO_3_	11
8	CoF_2_	Cs_2_CO_3_	Ag_2_O	32
9^b^	CoF_2_	Cs_2_CO_3_	Ag_2_CO_3_	84
10^c^	CoF_2_	Cs_2_CO_3_	Ag_2_CO_3_	80
11^d^	CoF_2_	Cs_2_CO_3_	Ag_2_CO_3_	77

^a^Reaction conditions: **1a** (0.2 mmol), **2a** (1.0 mL), Co-catalyst (20 mol %), oxidant (1.0 equiv), base (1.0 equiv), 100 °C, air, 12 h. ^b^DCE (1.0 mL) as co-solvent. ^c^PhCF_3_ (1.0 mL) as co-solvent. ^d^PhF (1.0 mL) as co-solvent. DCE = 1,2-dichloroethane.

**Scheme 1:**
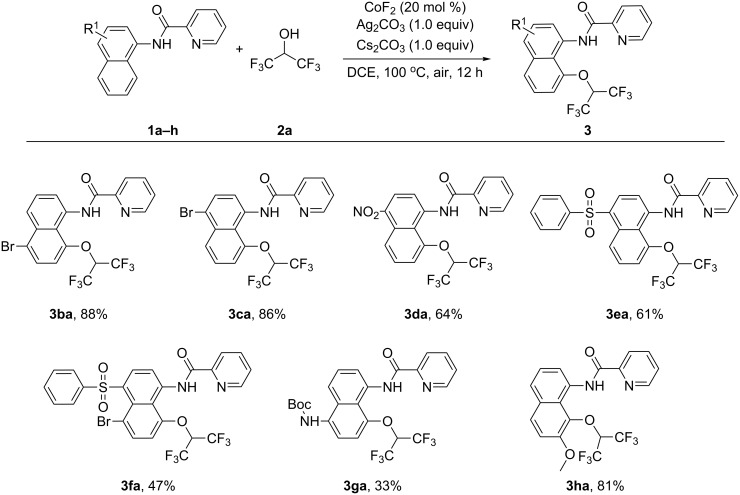
Reaction scope with respect to *N*-(naphthalen-1-yl)picolinamide derivatives. Reaction conditions: **1** (0.2 mmol), **2a** (1.0 mL), CoF_2_ (20 mol %), Ag_2_CO_3_ (1.0 equiv), Cs_2_CO_3_ (1.0 equiv), DCE (1.0 mL), 100 °C, air, 12 h.

With the established alkoxylated protocol in hand, the substrate scope of 1-naphthylamine derivatives was explored as shown in Scheme 1. Halogenated naphthylamines could afford the target products in 86–88% yields (**3ba**–**3ca**). Nitro- (**1d**) and benzenesulfonyl- (**1e**) substituted naphthylamines were found to proceed smoothly via this strategy (61–64%). In addition, a disubstituted naphthylamine provided the alkoxylated product in 47% yield (**3fa**). Moreover, a *Boc* amino group at C5 of the substrate **1g** was also compatible with the transformation (33%). When a methoxy group was located at the C7 site of the naphthylamine, sterically hindered product **3ha** was obtained in 81%. Besides, some benzylamine derivatives (*N*-(1-phenylethyl)picolinamide and *N*-benzhydrylpicolinamide) were attempted. However, no desired product could be detected.

**Scheme 2:**
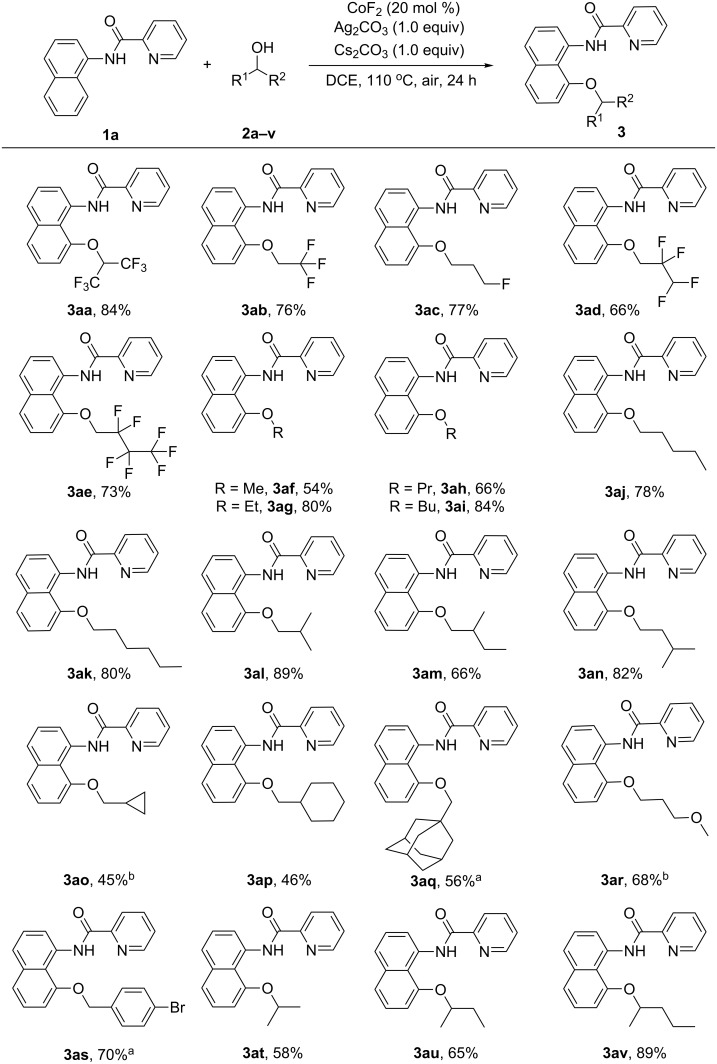
Reaction scope with respect to alcohols. Reaction conditions: **1a** (0.2 mmol), **2** (1.0 mL), CoF_2_ (20 mol %), Ag_2_CO_3_ (1.0 equiv), Cs_2_CO_3_ (1.0 equiv), DCE (1.0 mL), 110 °C, air, 24 h. ^a^**2q** or **2s** (5.0 equiv). ^b^48 h.

Next, the substrate scope of alcohols was investigated. As shown in Scheme 2, both primary and secondary alcohols were compatible with the slightly modified optimized conditions. A variety of fluoro-substituted alcohols **2a**–**e** proceeded smoothly to afford the corresponding products in moderate to good yields (66–84%). Simple primary alkyl alcohols were well tolerated to provide the desired products in 54–84% yields (**3af**–**ak**). Also, the branched alcohols **2l**, **2m**, and **2n** were employed to afford the corresponding products in 66–89% yields. Cyclopropylmethanol (**2o**), cyclohexylmethanol (**2p**), and adamantanemethanol (**2q**) were compatible with the transformation (45–56%). Moreover, an aliphatic ether and benzyl alcohol were proved to be effective coupling partners to provide **3ar** and **3as** in 68% and 70% yields, respectively. Compared with Co-catalyzed alkoxylation of arenes with primary alcohols [30–31], HFIP (**2a**), isopropanol (**2t**), isobutanol (**2u**), and isopentanol (**2v**) could all proceed smoothly to deliver the alkoxylated products in 58–89% yields. Furthermore, we attempted some tertiary alcohols (*tert-*butanol and 2-methyl-2-butanol). However, no desired product could be detected.

**Scheme 3:**
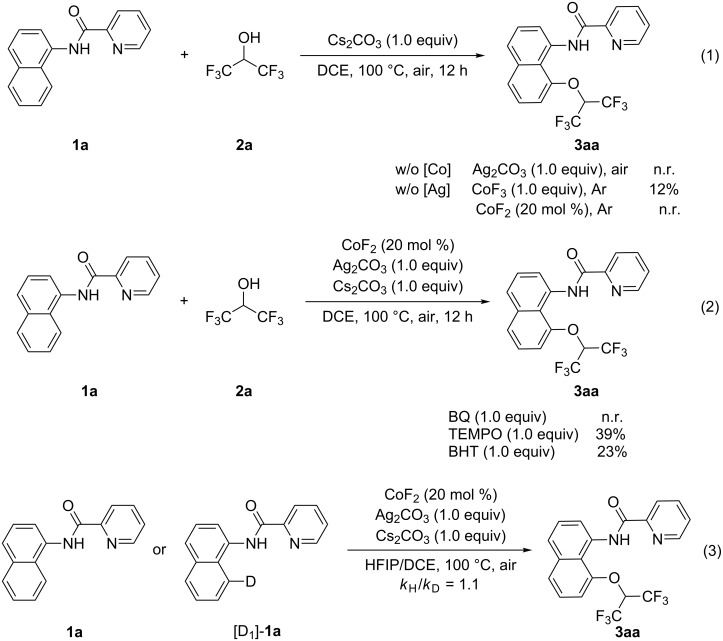
Control experiments and mechanistic studies.

In order to study the reaction mechanism, a series of control experiments were carried out (Scheme 3, see more details in Supporting Information File 1). In the absence of cobalt salt, no product was obtained under the standard reaction conditions. Under an argon atmosphere and without Ag_2_CO_3_, the product was isolated in 12% yield when a stoichiometric amount of CoF_3_ was introduced, whereas no product was obtained in the presence of CoF_2_ (Scheme 3, reaction 1). These results imply that the reaction should initiates from a Co^III^ species. The addition of a radical quencher, benzoquinone (BQ), suppressed the formation of product **3aa**. When 2,2,6,6-tetramethyl-1-piperidinyloxyl (TEMPO) or 2,6-di-*tert*-butyl-4-methylphenol (BHT) was added under the standard reaction conditions, a significantly reduced yield (39% or 23%) was obtained (Scheme 3, reaction 2). These facts suggest that a radical approach may be involved in the reaction. Moreover, in the parallel experiments, a KIE value of 1.1 was observed between **1a** or [D_1_]-**1a** with **2a**, which indicates that Co-catalyzed C–H bond cleavage should not be the rate determining step (Scheme 3, reaction 3).

**Scheme 4:**
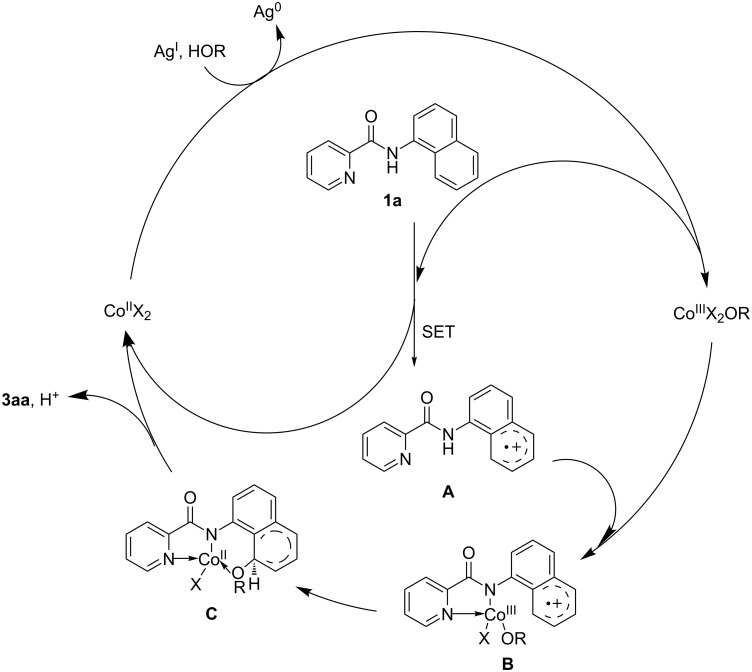
Proposed reaction mechanism.

On the basis of the above studies and previous literature [30–31,46–47], a plausible reaction mechanism for cobalt-catalyzed alkoxylation was proposed. As shown in Scheme 4, initially, Co^II^X_2_ could be oxidized to Co^III^X_2_OR in the presence of a silver salt and an alcohol. Based on the experiments and the density functional theory calculations (DFT) [30–31], the C–H activation most possibly proceeded via a single-electron transfer (SET) path compared to a concerted metalation-deprotonation (CMD) path. Followed by an intermolecular SET process, the cation-radical intermediate **A** was generated, which coordinates with a Co^III^ species to provide the intermediate **B**. Subsequently, the transfer of ligand OR from the coordinated Co^III^ to the naphthalene ring led to the formation of intermediate **C**. Finally, the alkoxylated product **3aa** was released accompanied with the deprotonation and the Co^II^ species was transformed into the Co^III^ species by re-oxidization.

**Scheme 5:**
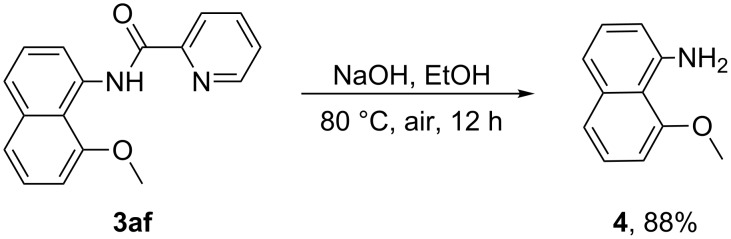
Removal of the directing group.

The current alkoxylation methodology also exhibited potential applications. When treated with NaOH at 80 °C, the picolinic acid directing group could be easily removed, and the corresponding **4** was obtained in 88% yield (Scheme 5).

## Conclusion

In summary, a cobalt-catalyzed C8 alkoxylation of naphthylamine derivatives with both primary and secondary alcohols was developed. This protocol is characterized by mild reaction conditions, broad substrate scope, and good functional group tolerance. Moreover, the excellent compatibility of fluorine-substituted alcohols (for instance, HFIP, trifluoroethanol, and 3-fluoropropanol etc.) shows that this strategy is highly valuable for the syntheses of biologically relevant fluorine-aryl ethers after the removal of the directing group. The above studies of the mechanism indicate that this reaction undergoes a SET process and cobalt salt is the actual catalyst. Overall, this protocol provides a new insight into the cobalt-catalyzed alkoxylation of naphthylamine derivatives. Further exploration of this strategy to aliphatic substrates is currently in progress.

## Supporting Information

File 1 Experimental details and characterization data of new compounds, and X-ray crystal structure details for **3aa**.

File 2 Crystallographic information file for compound **3aa**.
