# Supplementary material for: Cobalt-catalyzed peri-selective alkoxylation of 1-naphthylamine derivatives
Source: Beilstein J Org Chem. 2018 Aug 9;14:2090–7. doi: 10.3762/bjoc.14.183 (PMC6122339; doi:10.3762/bjoc.14.183)
Supplement: File 1 — Experimental details and characterization data of new compounds, and X-ray crystal structure details for 3aa. [file Beilstein_J_Org_Chem-14-2090-s001.pdf]

Supporting Information

for

Cobalt-catalyzed *peri*-selective alkoxylation of 1-naphthylamine derivatives

Jiao-Na Han, Cong Du, Xinju Zhu, Zheng-Long Wang, Yue Zhu, Zhao-Yang Chu, Jun-Long Niu* and Mao-Ping Song*

Address: College of Chemistry and Molecular Engineering, Zhengzhou University, Zhengzhou 450001, People's Republic of China

Email: Jun-Long Niu* - niujunlong@zzu.edu.cn; Mao-Ping Song* - mpsong@zzu.edu.cn

*Corresponding author

Experimental details and characterization data of new compounds, and X-ray crystal structure details for 3aa.

Table of Contents

General information S2
Experimental section.. S2
1. Optimization of reaction conditions.. S2
2. General procedure for the synthesis of **3**... S5
3. Control experimentsand mechanistic studies S5
4. Kinetic isotope effect measurements... S7
5. Removal of directing group S7
6. X-ray crystal structure details for **3aa**. S9
Reference S10
Characterization of products S11
NMR spectra.. S19

General information

Unless otherwise mentioned, all materials were commercially available and used without further purification. All the procedures were completed under the ambient air. ^{1}H NMR, ^{13}C NMR and ^{19}F NMR spectra were recorded at 400 (or 600) MHz, 101 (or 151) MHz or 376 MHz, respectively on a Bruker DPX instrument using Me_4Si as an internal standard. Chemical shift multiplicities are represented as follows: (s = singlet, d = doublet, t = triplet, q = quadruple,p = quintuple, m = multiplet, td = tripletdoublet, dt = doublet triplet,dd = double doublet, dq = doublet quadruple). Melting points were measured on a WC-1 instrument and uncorrected. New compounds for HRMS were tested on a Waters Q-Tof Micro MS/MS System ESI spectrometer. All the naphthylamine substrates were synthesized according to literature procedures [1,2].[D_1]-**1a** was prepared according to the literature [3].

Experimental section

1. Optimization of reaction conditions

Table S1. Optimization of catalysts.[a]

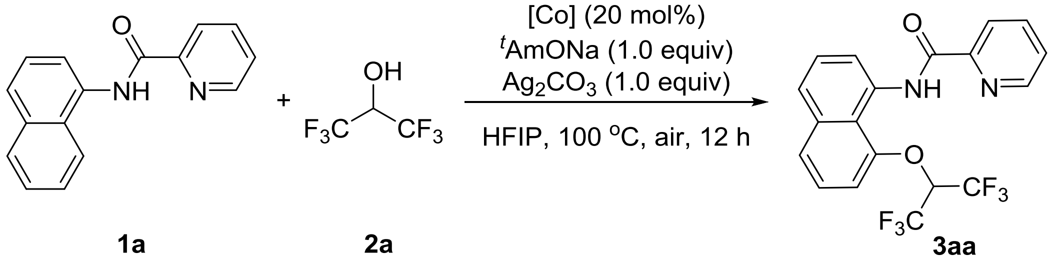

Entry	Catalyst	Yield (%)
1	$Co(OAc)_2 \cdot 4H_2O$	60
2	$Co(acac)_2$	34
3	$Co(acac)_3$	36
4	$CoCO_3 \cdot H_2O$	n.r.
5	CoO	n.r.
6	$Co(pph_3)Cl_2$	66
7	CoF_3	60
8	$CoSO_4 \cdot H_2O$	trace
9	$Co(OOCC_6H_5)_2$	63
10	CoF_2	71
11	$CoCl_2 \cdot 6H_2O$	57
12	$CoC_2O_4 \cdot 4H_2O$	56
13	$Co(NH_3)_6Cl_3$	53

14	$CoBr_2 \cdot 6H_2O$	65

[a]Reaction conditions:**1a** (0.2 mmol), **2a** (1.0 mL), Co Catalyst (20 mol %), Ag_2CO_3 (1.0 equiv), Cs_2CO_3 (1.0 equiv), 100 °C, air, 12 h. Isolated yield. HFIP = hexafluoroisopropanol. n.r. = no reaction.

Table S2. Optimization of base/acid.[a]

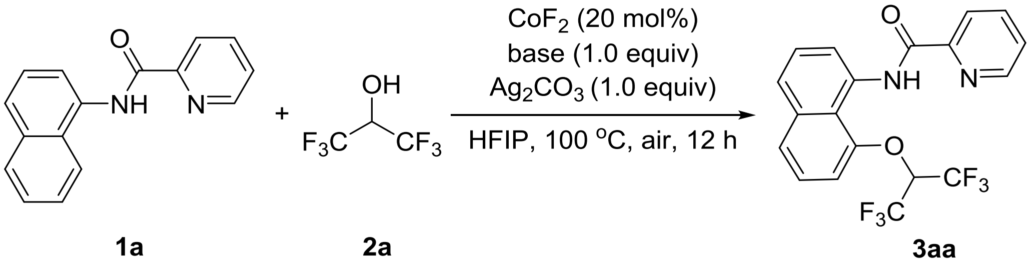

Entry	Base	Yield(%)
1	DBU	66
2	Et_3N	46
3	CH_3ONa	72
4	^{t}AmONa	71
5	EtONa	70
6	^{t}BuONa	65
7	CsOAc	8
8	NaOAc	12
9	Li_2CO_3	24
10	Na_2CO_3	58
11	K_2CO_3	67
12	Cs_2CO_3	82

[a]Reaction conditions:**1a** (0.2 mmol), **2a** (1.0 mL), CoF_2 (20 mol %), Ag_2CO_3(1.0 equiv), base (1.0 equiv), 100 °C, air, 12 h. Isolated yield.

Table S3. Optimization of oxidants.[a]

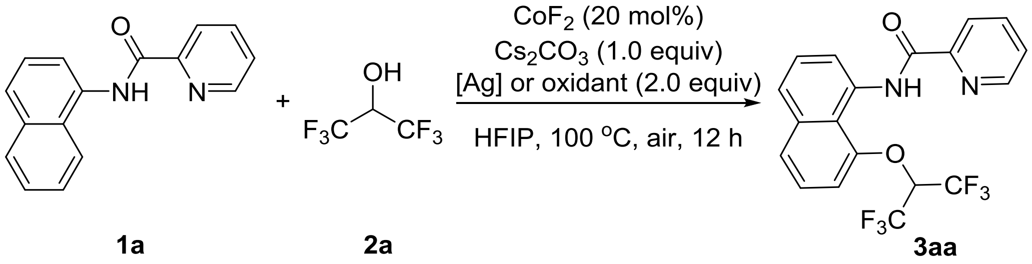

Entry	Oxidant	Yield(%)
1	$AgNO_3$	11
2	$NaIO_4$	trace
3	Ag_2CO_3	82
4	NMO	7
5	$K_2S_2O_8$	n.r.
6	$PhI(OAc)_2$	n.r.

7	Ag_2SO_4	trace
8	Ag_2O	32
9	AgTFA	trace
10	AgOAc	trace
11	$Mn(OAc)_2{\cdot}4H_2O$	trace
12	$Mn(acac)_2$	n.r.
13	$Mn(acac)_3$	n,r,

[a]Reaction conditions: **1a** (0.2 mmol), **2a** (1.0 mL),CoF_2 (20 mol %), oxidant (2.0 equiv), Cs_2CO_3(1.0 equiv), 100 ^oC, air, 12 h. Isolated yield.

Table S4. Optimization of cosolvents.[a]

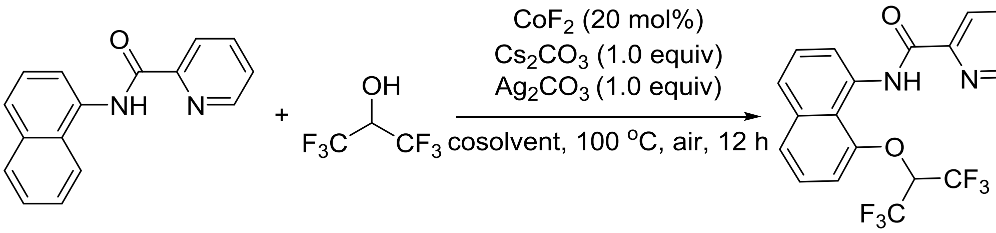

1a **2a** **3aa**

Entry	cosolvent	Yield(%)
1	DCE	84
2	$PhCF_3$	80
3	dioxane	15
4	PhMe	61
5	CH_3CN	19
6	THF	trace
7	PhF	77
8	TCP	75
9	PhOMe	79
10	acetone	22

[a]Reaction conditions:**1a** (0.2 mmol),**2a** (1.0 mL), CoF_2 (20 mol %), Ag_2CO_3 (1.0 equiv), Cs_2CO_3(1.0 equiv), cosolvent (1.0 mL), 100 ^oC, air, 12 h. Isolated yield.DCE = 1,2-dichloroethane. TCP = 1, 2, 3-Trichloropropane

Table S5. Optimization of temperature.[a]

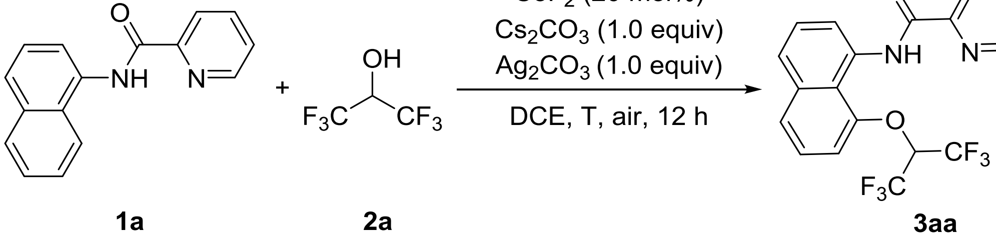

1a **2a** **3aa**

Entry	temperature	Yield (%)
1	60	trace

2	70	5
3	80	37
4	90	48
5	100	84
6	110	75
7	120	63

[a]Reaction conditions:**1a** (0.2 mmol), CoF_2 (20 mol %), Ag_2CO_3 (1.0 equiv), Cs_2CO_3 (1.0 equiv), HFIP (1.0 mL), DCE (1.0 mL), temperature, air, 12 h. Isolated yield.

2. General procedure for the synthesis of 3

A 15 mL oven-dried screw cap tube equipped with a magnetic stir bar was filled with **1** (0.20 mmol), Ag_2CO_3 (55.2 mg, 0.20 mmol), Cs_2CO_3 (65.0 mg, 0.20 mmol,), CoF_2 (3.8 mg, 20 mol %), **2** (1.0 mL) and DCE (1.0 mL). The reaction was stirred at 100 ^oC for 12 h and then cooled down to room temperature. The resulting mixture was diluted with 25 mL of dichloromethane, and filtered through a celite pad. The reaction solution was detected by TLC, and concentrated in vacuum. The product was purified bypreparative TLC on silica gel (petroleum ether/EtOAc) to afford the target product **3**.

3. Control experimentsand mechanistic studies

Control experiments

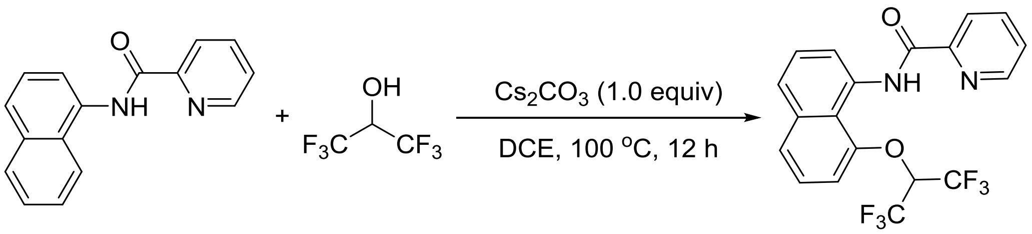

1a **2a** **3aa**

Entry	Catalyst	oxidant	atmosphere	Yield (%)
1	CoF_2	--	Ar	n.r.
2	CoF_2	--	air	29
3	CoF_2	--	O_2	51
4	--	Ag_2CO_3	air	n.r.
5	CoF_3	Ag_2CO_3	Ar	67
6[b]	CoF_3	--	Ar	12
7[c]	CoF_2	Ag_2CO_3	air	n.r.
8[d]	CoF_2	Ag_2CO_3	air	39
9[e]	CoF_2	Ag_2CO_3	air	23

[a]Reaction conditions: **1a** (0.2 mmol), **2a** (1.0 mL), Co salts (20 mol %),

Ag_2CO_3(1.0 equiv), Cs_2CO_3(1.0 equiv), DCE (1.0 mL), 100 ^oC, air, 12 h. Isolated yield.[b]CoF_3 (1.0 equiv).[c]BQ (1.0 equiv) was added. [d]TEMPO (1.0 equiv) was added. [e]BHT (1.0 equiv) was added.

Reaction of radicals trapping

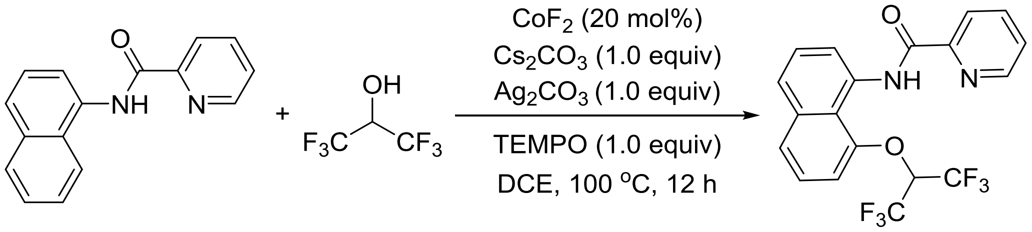

1a **2a** **3aa**

A 15 mL oven-dried screw cap tube equipped with a magnetic stir bar was filled with **1a** (49.6 mg, 0.20 mmol), Ag_2CO_3 (55.2 mg, 0.20 mmol), Cs_2CO_3 (65.0 mg, 0.20 mmol,), CoF_2 (3.8 mg, 20 mol %),TEMPO (1.0 equiv), HFIP (1.0 mL) and DCE (1.0 mL). Then the reaction was stirred in an oil bath at 100 ^oC for 12 h and cooled down to room temperature. The resulting mixture was diluted with 25 mL of dichloromethane, and filtered through a celite pad. The reaction solution was detected by TLC, and concentrated in vacuum. The product was purified bypreparative TLC on silica gel (petroleum ether/EtOAc = 5:1)to afford the target product **3aa** in 39% yield.

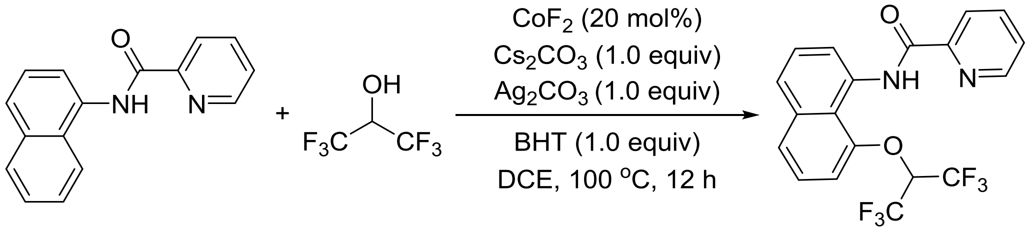

1a **2a** **3aa**

A 15 mL oven-dried screw cap tube equipped with a magnetic stir bar was filled with **1a** (49.6 mg, 0.20 mmol), Ag_2CO_3 (55.2 mg, 0.20 mmol), Cs_2CO_3 (65.0 mg, 0.20 mmol,), CoF_2 (3.8 mg, 20 mol %), BHT (1.0 equiv), HFIP (1.0 mL) and DCE (1.0 mL). Then the reaction was stirred in an oil bath at 100 ^oC for 12 h and cooled down to room temperature. The resulting mixture was diluted with 25 mL of dichloromethane, and filtered through a celite pad. The reaction solution was detected by TLC, and concentrated in vacuum. The product was purified by preparative TLC on silica gel (petroleum ether/EtOAc = 5:1) to afford the target product **3aa** in 23% yield.

4. Kinetic isotope effect measurements

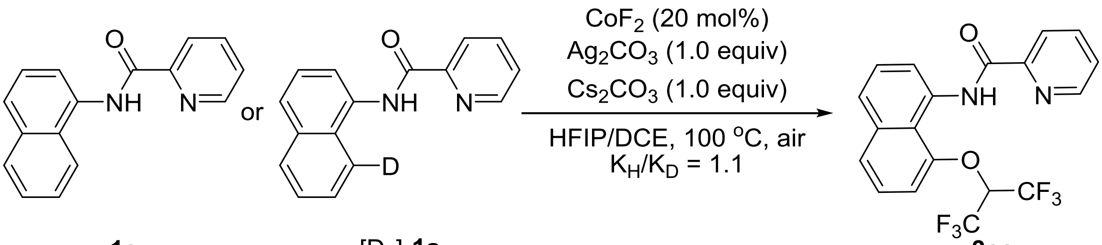

1a [D$_1$]-**1a** **3aa**

A 15 mL oven-dried screw captube was equipped with a magnetic stir bar and charged with **1a** (49.6 mg, 0.2 mmol) or [D$_1$]-**1a** (49.9 mg, 0.2 mmol), **2a** (1.0 mL), Ag_2CO_3 (55.2 mg, 0.20 mmol), Cs_2CO_3 (65.0 mg, 0.20 mmol,), CoF_2 (3.8 mg, 20 mol %) and DCE (1.0 mL). Then the tubes were heated at 100 ^oC for 20, 22, 24, 26 minutes and quenched separately with 1mL dichloromethane. Next, the reaction mixture was diluted with 25 mL dichloromethane and filtered through a celite pad. The solvent was removed in vacuum and ^1H NMR was taken separately using 0.1 mmol anisole (10.8 mg) as the internal standard. The KIE was determined as $k_H/k_D \approx 1.1$.

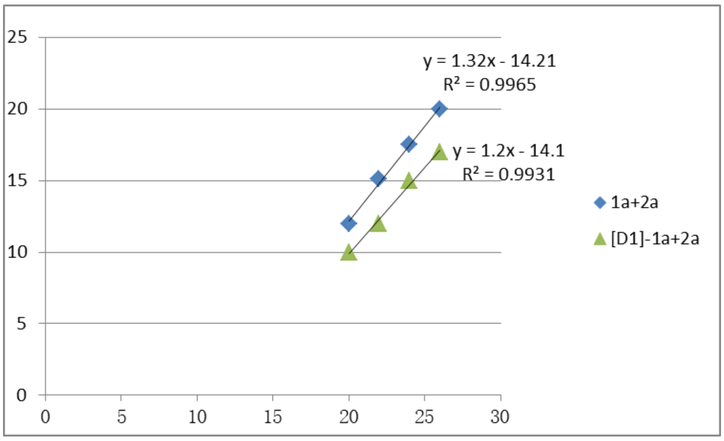

5. Removal of directing group

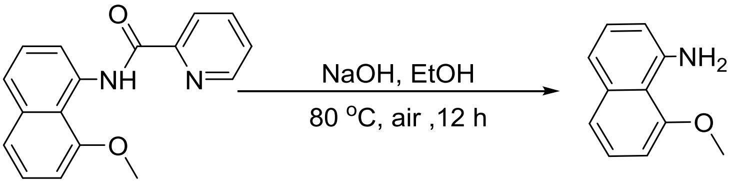

3af **4**, 88%

A 15 mL oven-dried screw cap tube was equipped with a magnetic stir bar and charged with **3af** (13.9 mg, 0.05 mmol), NaOH (40.0 mg, 1 mmol, 20 equiv) and MeOH (0.5 mL). Then the reaction was stirred in an oil bath at 100 ^oC for 12 h. After completion, the resulting mixture was cooled to room temperature, quenched with 5 mL H_2O, then treated with HCl (2.0 mol/L) until pH = 5. Next, the reaction mixture was diluted with dichloromethane

(10 ml × 2). The organic layer was collected and dried over Na_2SO_4. After concentration in vacuum, the residue was purified by preparative TLC on silica gel and the product was isolated in 88%. ^{1}H NMR (400 MHz, $CDCl_3$): δ 7.35-7.28 (m, 1H), 7.28-7.16 (m, 2H), 7.15-7.08 (m, 1H), 6.69 (dd, J = 7.5, 0.7 Hz, 1H), 6.57 (dd, J = 7.5, 1.1 Hz, 1H), 3.96 (s, 9H).^{13}C NMR (101 MHz, $CDCl_3$): δ 157.7, 144.9, 137.3, 127.1, 125.6, 121.5, 116.9, 115.1, 109.7, 103.6, 55.7.

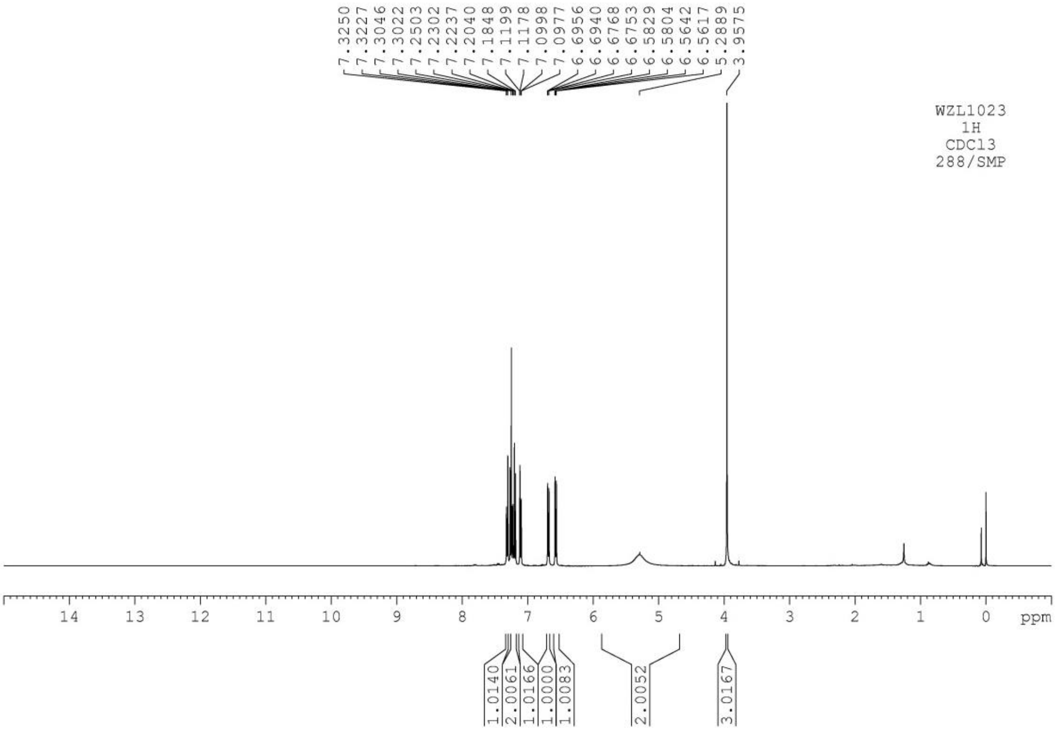

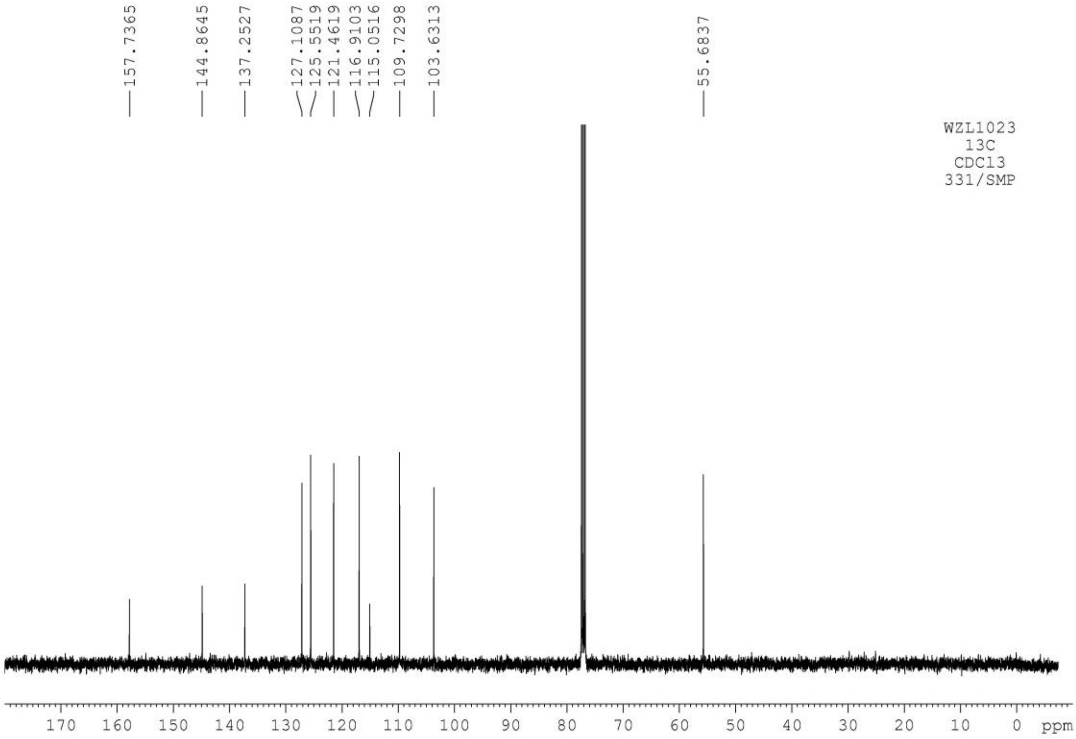

6. X-ray crystal structure details for 3aa

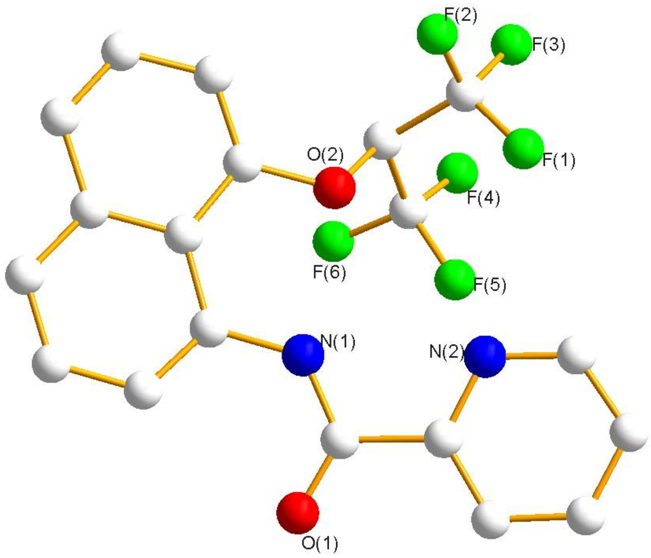

**Chemical formula**	$C_{19}H_{12}F_6N_2O_2$
**Formula weight**	414.31 g/mol
**Temperature**	293(2) K

**Wavelength**	1.54184Å	
**Crystal system**	orthorhombic	
**Space group**	Pbca	
**Unit cell dimensions**	*a* = 7.3762(3)Å	α = 90°
	b = 17.5081(5) Å	β = 90°
	c = 28.0730(11) Å	γ = 90°
**Volume**	3625.4(2) $Å^3$	
**Z**	8	
**Density (calculated)**	1.518 g/cm^3	
**Absorption coefficient**	1.249 mm^{-1}	
**F(000)**	1680.0	

Data collection and structure refinement for compound 3aa.

**Theta range for datacollection**	10.104 to 134.16^o
**Index ranges**	-8 ≤ h ≤ 6, -13 ≤ k ≤ 20, -21 ≤ l ≤ 33
**Reflections collected**	8287
**Independent reflections**	3240 [R(int) = 0.0291]
**Absorption correction**	Multi-Scan
**Structure solution technique**	direct methods
**Structure solution program**	SHELX,VERSION 2014/7

Reference:

1. Bai, P.; Sun, S.; Li, Z.; Qiao, H.; Su, X.; Yang, F.; Wu, Y.*J. Org. Chem.***2017**, *82*, 12119-12127.
2. Li, Z.; Sun, S.; Qiao, H.; Yang, F.; Zhu, Y.; Kang, J.; Wu, Y. J. *Org. Lett.* **2016**, *18*, 4594-4597.
3. Huang, L.; Li, Q.; Wang, C.; Qi, C. *J. Org. Chem.***2013**, *78*, 3030-3038.

Characterization of products

***N*-(8-(Hexafluoropropan-2-yl)oxy)naphthalen-1-yl)picolinamide (3aa)**: purified by analytical TLC on silica gel with petroleum ether/EtOAc (5:1) as an eluent R_f = 0.41; light yellow solid (69.6 mg, 84%), mp 153-158^oC. ^1H NMR(400 MHz, CDCl3): δ 11.85 (s, 1H), 8.92 (dd, *J* = 7.7, 1.2 Hz, 1H), 8.65 (d, *J* = 4.5 Hz, 1H), 8.31 (d, *J* = 7.8 Hz, 1H), 7.91-7.87 (m, 1H), 7.66-7.55 (m, 3H), 7.47-7.44 (m, 1H), 7.41 (t, *J* = 11.9 Hz, 1H), 7.12 (d, *J* =11.6 Hz, 1H), 5.24-5.15 (m, 1H). ^{13}C NMR (101 MHz, CDCl3): δ 162.8, 154.9, 150.2, 147.9, 137.2, 136.6, 133.4, 127.4, 126.2, 125.9, 125.2, 124.3, 122.3, 120.9 (dq, J_{C-F} = 284.7, 2.9Hz), 119.2, 117.8, 116.7, 78.1 (p, J_{C-F} = 34.0Hz). HRMS (positive ESI) Calcd. ^{19}F NMR (376 MHz, $CDCl_3$): δ -72.46. For $C_{19}H_{12}F_6N_2O_2$ (M + H) 415.0881, Found: 415.0881.

***N*-(5-Bromo-8-(hexafluoropropan-2-yl)oxy)naphthalen-1-yl)picolinamide (3ba)**: purified by analytical TLC on silica gel with petroleum ether/EtOAc (5:1) as an eluent R_f = 0.50; white solid (86.8 mg, 88%), mp 152-156^oC.^1H NMR (400 MHz, $CDCl_3$): δ 11.82 (s, 1H), 8.97 (dd, *J* = 7.9, 1.0 Hz, 1H), 8.61 (d, *J* = 4.4 Hz, 1H), 8.31 (dt, *J* = 7.8, 1.0 Hz, 1H), 8.10 (dd, *J* = 8.5, 1.0 Hz, 1H), 7.93-7.88 (m, 1H), 7.75-7.68 (m, 2H), 7.49-7.46 (m, 1H), 7.00 (d, *J* = 8.4 Hz, 1H), 5.15-5.06 (m, 1H). ^{13}C NMR (101 MHz, CDCl3): δ 162.8, 154.7, 150.0, 147.9, 137.3, 134.2, 133.8, 129.3, 128.9, 126.4, 123.8, 122.4, 120.8 (dq, J_{C-F} = 285.3, 3.8 Hz), 120.3, 119.4, 119.0, 111.1, 78.5 (p, J_{C-F} = 33.7 Hz).^{19}F NMR (376 MHz, $CDCl_3$): δ -72.42. HRMS (positive ESI) Calcd. For $C_{19}H_{11}BrF_6N_2O_2$ (M + H) 492.9986, Found: 492.9986.

***N*-(4-Bromo-8-(hexafluoropropan-2-yl)oxy)naphthalen-1-yl)picolinamide (3ca):** purified by analytical TLC on silica gel with petroleum ether/EtOAc (5:1) as an eluent R_f = 0.47; white solid (84.8 mg, 86%), mp 122-126 ^oC. ^1H NMR (400 MHz, $CDCl_3$): δ 11.88 (s, 1H), 8.82 (d, *J* = 8.6 Hz, 1H), 8.60 (d, *J* = 4.4 Hz, 1H), 8.29 (dt, *J* = 7.8, 0.9 Hz, 1H), 8.15 (dd, *J* = 8.6, 0.9 Hz, 1H), 7.91-7.86 (m, 2H), 7.57-7.42 (m, 2H), 7.22-7.21 (d, *J* = 8.6, 1H), 5.20-5.11 (m,1H). ^{13}C NMR (101 MHz, $CDCl_3$): δ 162.8, 155.1, 150.0, 147.9, 137.4, 134.2, 133.5, 131.6, 126.5, 126.4, 125.3, 122.4, 120.7(dq, J_{C-F} = 284.8, 2.8 Hz), 119.3, 119.2, 117.6, 112.0, 78.7 (p, J_{C-F} = 34.6 Hz).^{19}F NMR (376 MHz, $CDCl_3$): δ -72.44. HRMS (positive ESI) Calcd. For $C_{19}H_{11}BrF_6N_2O_2$ (M + H) 492.9986, Found: 492.9985.

***N*-(4-Nitro-8-(hexafluoropropan-2-yl)oxy)naphthalen-1-yl)picolinamide (3da):** purified by analytical TLC on silica gel with petroleum ether/EtOAc (5:1) as an eluent R_f = 0.40; yellow solid (58.8 mg, 64%), mp 176-178^oC. ^1H NMR (400 MHz, $CDCl_3$): δ 12.30 (s, 1H), 9.15 (d, *J* = 8.9 Hz, 1H), 8.62 (d, *J* = 4.4 Hz, 1H), 8.48 (dd, *J* = 8.8, 0.7 Hz, 1H), 8.33 (dd, *J* = 8.3, 5.3 Hz, 2H), 7.96-7.91 (m, 1H), 7. 68 (t, *J* = 8.0, Hz 1H), 7.54-7.52 (m, 1H), 7.31 (d, *J* = 7.9 Hz, 1H), 5.21-5.07 (m, 1H). ^{13}C NMR (101 MHz, $CDCl_3$): δ 163.3, 155.4, 149.3, 148.1, 142.2, 139.3, 137.6, 128.7, 128.5, 126.9, 126.6, 122.7, 120.8, 120.7(dq, J_{C-F} = 284.5, 2.7 Hz),117.8, 115.9, 112.8, 79.1 (p, J_{C-F} = 34.1 Hz).^{19}F NMR (376 MHz,

$CDCl_3$): δ -72.35. HRMS (positive ESI) Calcd. For $C_{19}H_{11}F_6N_3O_2$ (M + K) 498.0291, Found: 498.0287.

***N*-(4-Benzenesulfonyl-8-(hexafluoropropan-2-yl)oxy)naphthalen-1-yl)picolinamide (3ea):**purified by analytical TLC on silica gel with petroleum ether/EtOAc (1:1) as an eluent R_f = 0.53; white solid (67.6 mg, 61%), mp 184-188°C. ^{1}H NMR (400 MHz, $CDCl_3$): δ 12.23 (s, 1H), 9.18 (d, *J* = 8.6 Hz, 1H), 8.64-8.52 (m, 3H), 8.30(d, *J* = 7.8 Hz, 1H), 7.95-7.89 (m, 3H), 7.56-7.46 (m, 5H), 7.21 (d, *J* = 7.8, 1H), 5.16-5.06 (m, 1H). ^{13}C NMR (101 MHz, $CDCl_3$): δ 163.3, 155.5, 149.4, 148.0, 141.7, 139.7, 137.5, 133.1, 132.5, 131.8, 130.3, 129.2, 127.8, 127.4, 126.8, 122.6, 121.9, 120.7 (dq, J_{C-F} = 284.5, 2.7 Hz), 118.2, 116.2, 112.3, 78.8 (p, J_{C-F} = 34.3 Hz).^{19}F NMR (376 MHz, $CDCl_3$): δ -72.35. HRMS (positive ESI) Calcd. For $C_{25}H_{16}F_6N_2O_4S$ (M + H) 555.0813, Found: 555.0813.

***N*-(4-Benzenesulfonyl-5-bromo-8-(hexafluoropropan-2-yl)oxy)naphthalen-1-yl)picolinamide (3fa):** purified by analytical TLC on silica gel with petroleum ether/EtOAc (3:1) as an eluent R_f = 0.29; white solid (59.5 mg, 47%), mp 232-236 °C.^{1}H NMR (400 MHz, CDCl3): δ 12.00 (s, 1H), 9.54 (d, J = 1.8 Hz, 1H), 8.77 (d, J = 1.8 Hz, 1H), 8.58 (d, J = 4.5 Hz, 1H), 8.30 (d, J = 7.8 Hz, 1H), 8.15-8.12 (m, 2H), 7.92 (td, J = 7.7, 1.7 Hz, 1H), 7.86 (d, J = 8.4 Hz, 1H), 7.63-7.53 (m, 3H), 7.51-7.48 (m, 1H), 7.15 (d, J = 8.5 Hz, 1H), 5.12-5.06 (m, 1H).^{13}C NMR (101 MHz, $CDCl_3$): δ 163.0, 154.5, 149.4, 148.0, 141.8, 140.8, 137.5, 136.0, 133.7, 133.6, 131.1, 129.5, 128.2, 126.7, 123.3, 122.6, 120.9 (dq, J_{C-F} = 284.7, 2.3 Hz), 120.8, 120.4, 116.0, 114.0, 78.7(p, J_{C-F} = 34.3 Hz).^{19}F NMR (376 MHz, $CDCl_3$): δ-72.31.HRMS (positive ESI) Calcd. For $C_{25}H_{15}BrF_6N_2O_4S$ (M + H) 632.9918, Found: 632.9913.

***N*-(5-*tert*-Butylmethylcarbamate-8-(hexafluoropropan-2-yl)oxy)naphthalen-1-yl)picolinamide (3ga):** purified by analytical TLC on silica gel with petroleum ether/EtOAc (3:1) as an eluent R_f = 0.60; white solid (43.9mg, 33%), mp 157-161°C. ^{1}H NMR (400 MHz, $CDCl_3$): δ 11.85 (s, 1H), 8.91 (dd, *J* = 7.8, 0.9 Hz, 1H), 8.62 (d, *J* = 4.3 Hz, 1H), 8.30 (d, *J* = 7.8 Hz, 1H), 7.92-7.88 (m, 1H), 7.77 (d, *J* = 8.0 Hz, 1H), 7.68 (d, *J* = 7.9 Hz, 1H), 7.58 (t, *J* = 8.2 Hz, 1H), 7.49-7.45 (m, 1H), 7.09 (d, *J* = 8.6 Hz, 1H), 6.81 (s, 1H), 5.12-5.04 (m, 1H), 1.55 (s, 9H). ^{13}C NMR (101 MHz, $CDCl_3$): δ 162.8, 153.7, 152.0, 150.2, 148.0, 137.3, 134.0, 130.3, 127.7, 126.3, 122.4,121.1(dq, J_{C-F} = 286.1, 3.5 Hz), 119.4, 118.2, 116.8, 111.0, 81.0,78.8(p, J_{C-F} = 33.8 Hz), 28.4.^{19}F NMR (376 MHz, $CDCl_3$): δ -72.52. HRMS (positive ESI) Calcd. For $C_{24}H_{21}F_6N_3O_4$ (M + H) 530.1515, Found: 530.1509.

***N*-(7-Methoxy-8-(hexafluoropropan-2-yl)oxy)naphthalen-1-yl)picolinamide (3ha):** purified by analytical TLC on silica gel with petroleum ether/EtOAc (3:1) as an eluent R_f = 0.36; light yellow solid (72.0 mg, 81%), mp 155-157°C. ^{1}H NMR (400 MHz, $CDCl_3$): δ 11.76 (s, 1H), 8.89 (dd, *J* = 7.8, 1.1 Hz, 1H), 8.62 (d, *J* = 4.3 Hz, 1H), 8.31 (dt, *J* = 7.8, 1.0 Hz, 1H), 7.90-7.86 (m, 1H), 7.69 (d, *J* = 9.1 Hz, 1H), 7.55 (dd, *J* = 8.1, 0.8 Hz, 1H), 7.46-7.41 (m, 2H), 7.30 (d, *J* = 9.0 Hz, 1H), 5.38-5.30 (m, 1H), 4.03 (s, 3H). ^{13}C NMR (101 MHz, $CDCl_3$): δ 162.7,

150.4, 148.0, 146.8, 140.3, 137.2, 132.6, 131.0, 127.3, 126.1, 125.0, 124.2, 122.2, 121.2 (dq, $J_{C\text{-}F}$ = 285.2, 2.8 Hz), 120.1, 119.5, 114.4, 76.4 (p, $J_{C\text{-}F}$ = 33.8 Hz), 57.1.^{19}F NMR (376 MHz, $CDCl_3$): δ -71.97. HRMS (positive ESI) Calcd. For $C_{20}H_{14}F_6N_2O_3$ (M + H) 445.0987, Found: 445.0982.

***N*-(8-(2,2,2-Trifluoroethoxy)naphthalen-1-yl)picolinamide (3ab)**: purified by analytical TLC on silica gel with petroleum ether/EtOAc (5:1) as an eluent R_f = 0.31; light yellow solid (52.6mg, 76%), mp 200-203 ^{o}C.^{1}H NMR (400 MHz, $CDCl_3$): δ 12.50 (s, 1H), 28.94 (d, *J* = 7.6 Hz, 1H), 8.66 (d, *J* = 4.6 Hz, 1H), 8.34 (d, *J* = 7.8 Hz, 1H), 7.92 (td, *J* = 7.7, 1.5 Hz, 1H), 7.62-7.48 (m, 4H), 7.40 (t, *J* = 8.0 Hz, 1H), 7.07 (d, *J* = 7.7 Hz, 1H), 4.77 (q, *J* = 8.4 Hz, 2H).^{13}C NMR (101 MHz, $CDCl_3$): δ 162.8, 154.8, 150.7, 148.1, 137.5, 136.5, 134.1, 127.1, 126.2, 125.4, 124.5, 124.2, 123.4 (q, $J_{C\text{-}F}$ = 285.3 Hz) 122.1, 118.1, 117.3, 109.8, 36.2(q, $J_{C\text{-}F}$ = 36.3 Hz). ^{19}F NMR (376 MHz, $CDCl_3$): δ -72.83. HRMS (positive ESI) Calcd. For $C_{18}H_{13}F_3N_2O_3$ (M + H) 347.1007, Found: 347.1008.

***N*-(8-(3-Fluoropropoxy)naphthalen-1-yl)picolinamide (3ac)**: purified by analytical TLC on silica gel with petroleum ether/EtOAc (1:1) as an eluent R_f = 0.65; brown solid (50.0mg, 77%), mp 103-105 ^{o}C. ^{1}H NMR (400 MHz, $CDCl_3$): δ 12.59 (s, 1H), 8.99 (dd, *J* = 7.7, 1.3 Hz, 1H), 8.63-8.61 (m, 1H), 8.37 (dt, *J* = 7.9, 1.0 Hz, 1H), 7.91 (td, *J* = 7.7, 1.7 Hz, 1H), 7.59-7.56 (m, 1H), 7.53-7.45 (m, 3H) 7.35 (t, *J* = 7.9 Hz, 1H), 6.95 (d, *J* = 7.3 Hz, 1H), 4.71 (t, *J* = 5.8 Hz, 1H), 4.59 (t, *J* = 5.8 Hz, 1H), 4.47 (t, *J* = 6.1 Hz, 2H), 2.60-2.56 (m, 1H), 2.52-2.48 (m, 1H).^{13}C NMR (101 MHz, $CDCl_3$): δ 162.6, 155.3, 151.0, 147.7, 137.6, 136.5, 134.8, 126.7, 126.2, 125.6, 124.1, 122.9, 122.3, 117.4, 117.0, 106.8, 80.9(d, $J_{C\text{-}F}$ = 164.8Hz), 65.4(d, $J_{C\text{-}F}$ = 5.4Hz), 29.9(d, $J_{C\text{-}F}$ = 20.2Hz).^{19}F NMR (376 MHz, $CDCl_3$): δ -72.83. HRMS (positive ESI) Calcd. For $C_{19}H_{16}FN_2O_2$ (M + H) 325.1352, Found: 325.1347.

***N*-(8-(2,2,3,3-Tetrafluoropropoxy)naphthalen-1-yl)picolinamide (3ad)**: purified by analytical TLC on silica gel with petroleum ether/EtOAc (3:1) as an eluent R_f = 0.44; light green solid (50.0mg, 66%), mp 133-136°C. ^{1}H NMR (400 MHz, $CDCl_3$): δ 12.18 (s, 1H), 8.91 (dd, *J* = 7.7, 1.2 Hz, 1H), 8.64 (d, *J* = 4.5 Hz, 1H), 8.34 (td, *J* = 7.8, 0.9 Hz, 1H), 7.91 (td, *J* = 7.7, 1.7 Hz, 1H), 7.62-7.59 (m, 1H), 7.62-7.59 (m, 1H), 7.57-7.46 (m, 3H), 7.37 (t, *J* = 8.0 Hz, 1H), 7.05 (d, *J* = 7.7 Hz, 1H), 6.23 (tt, *J* = 52.9, 4.5 Hz, 1H), 4.76 (t, *J* = 12.8 Hz, 2H).^{13}C NMR (101 MHz, $CDCl_3$): δ 162.6, 154.7, 150.5, 148.1 137.6, 136.5, 133.9, 126.9, 126.4, 125.4, 124.4, 124.2 122.7, 117.2 (tt, $J_{C\text{-}F}$ = 34.3, 250.3Hz) 118.4, 117.3,111.7(tt, $J_{C\text{-}F}$ = 34.3, 250.3Hz), 109.1, 67.2 (d, $J_{C\text{-}F}$ = 28.9 Hz).^{19}F NMR (376 MHz, $CDCl_3$): δ-123.15, -138.46. HRMS (positive ESI) Calcd. For $C_{19}H_{14}F_4N_2O_2$ (M + H) 379.1070, Found: 379.1064.

***N*-(8-(2,2,3,3,4,4,4-Heptafluorobutoxy)naphthalen-1-yl)picolinamide (3ae)**:purified by analytical TLC on silica gel with petroleum ether/EtOAc (5:1) as an eluent R_f = 0.38; white solid (65.2mg, 73%), mp 130-134°C. ^{1}H NMR (400 MHz, $CDCl_3$): δ 12.49 (s, 1H), 8.94 (dd, *J* = 7.7, 1.3 Hz, 1H), 8.60 (d, *J* = 4.3 Hz, 1H), 8.33 (dt, *J* = 7.8, 1.0 Hz, 1H), 7.91 (td, *J* = 7.7, 1.7 Hz, 1H), 7.61-7.51 (m, 3H), 7.49-7.46 (m, 1H), 7.38 (t, *J* = 8.1 Hz, 1H), 7.07 (d, *J* = 7.7

Hz, 1H), 4.92 (t, *J* = 14.7 Hz, 2H).^{13}C NMR (151 MHz, CDCl3): δ 161.6, 154.0, 149.7, 146.9, 136.5, 135.5, 133.1, 126.1, 125.2, 124.4, 123.6, 123.2, 121.6, 117.5 (t, *J* = 43.7 Hz), 117.1, 115.5 (q, *J* = 37.3 Hz), 116.4, 112.8 (t, *J* = 260.6, 31.0 Hz), 109.1, 66.4 (t, *J* = 23.4 Hz).^{19}F NMR (376 MHz, $CDCl_3$): δ -119.68,-119.74, -127.67. HRMS (positive ESI) Calcd. For $C_{20}H_{13}F_7N_2O_2$ (M + H) 447.0944, Found: 447.0938.

***N*-(8-Methoxynaphthalen-1-yl)picolinamide (3af)**: purified by analytical TLC on silica gel with petroleum ether/EtOAc (3:1) as an eluent R_f = 0.30; light yellow solid (30.1mg, 54%), mp 176-182°C. ^{1}H NMR (400 MHz, $CDCl_3$): δ13.20 (s, 1H), 9.00 (dd, *J* = 7.6, 1.4 Hz, 1H), 8.71-8.69 (m, 1H), 8.35 (dt, *J* = 7.9, 0.9 Hz, 1H), 7.91 (td, *J* = 7.7, 1.7 Hz, 1H), 7.57-7.55 (m, 1H), 7.53-7.44 (m, 3H), 7.37 (t, *J* = 8.0 Hz 1H), 6.91 (dd, *J* = 7.6, 0.7 Hz, 1H), 4.20 (s, 3H).^{13}C NMR (101 MHz, $CDCl_3$): δ 162.4, 156.3, 151.2, 148.1, 137.5, 136.4, 135.2, 126.8, 126.1, 125.6, 123.7, 122.6, 122.1, 116.6, 116.6, 105.7, 56.2.HRMS (positive ESI) Calcd. For $C_{17}H_{14}N_2O_2$ (M + H) 279.1134, Found: 279.1128

***N*-(8-Ethoxynaphthalen-1-yl)picolinamide (3ag):**purified by analytical TLC on silica gel with petroleum ether/EtOAc (3:1) as an eluent R_f = 0.39; white solid (46.8mg, 80%), mp 105-111°C.^{1}H NMR (400 MHz, $CDCl_3$): δ 12.75 (s, 1H), 9.01 (dd, *J* = 7.7, 1.3 Hz, 1H), 8.63-8.61(m, 1H), 8.34 (dt, *J* = 7.9, 0.9 Hz, 1H), 7.88 (td, *J* = 7.7, 1.7 Hz, 1H), 7.57-7.55 (m, 1H), 7.52-7.40 (m, 3H), 7.33 (t, *J* = 8.0 Hz 1H), 6.90 (d, *J* = 7.4 Hz, 1H), 4.35 (q, *J* = 7.0 Hz, 2H), 1.68 (t, *J* = 7.0 Hz, 3H). ^{13}C NMR (101 MHz, $CDCl_3$): δ 162.8, 155.6, 151.1, 147.7, 137.4, 136.5, 135.0, 126.6, 126.1, 125.6, 124.0, 122.7, 121.9, 117.2, 117.0, 106.8, 65.4, 15.1.HRMS (positive ESI) Calcd. For $C_{18}H_{16}N_2O_2$ (M + H) 293.1290, Found: 293.1285.

***N*-(8-Propoxynaphthalen-1-yl)picolinamide (3ah)**: purified by analytical TLC on silica gel with petroleum ether/EtOAc (3:1) as an eluent R_f = 0.43; white solid (40.4mg, 66%), mp 82-84°C ^{1}H NMR (400 MHz, $CDCl_3$): δ 12.74 (s, 1H), 8.99 (dd, *J* = 7.7, 1.2 Hz, 1H), 8.66-8.64 (m, 1H), 8.35 (d, *J* = 7.8 Hz, 1H), 7.92 (td, *J* = 7.7, 1.7 Hz, 1H), 7.58-7.56 (m, 1H), 7.52-7.46 (m, 2H), 7.45-7.43 (m, 1H), 7.35(t, *J* = 8.0 Hz, 1H), 6.93 (d, *J* = 7.8 Hz, 1H), 4.30 (t, *J* = 6.7 Hz, 2H), 2.20-2.11 (m, 2H), 1.04 (t, *J* = 7.4 Hz, 3H). ^{13}C NMR (101 MHz, $CDCl_3$): δ 162.8, 155.7, 151.2, 147.6, 137.4, 136.5, 135.0, 126.6, 126.1, 125.6, 124.0, 122.8, 121.8, 117.2, 117.1, 106.8, 71.5, 22.3, 10.8.HRMS (positive ESI) Calcd. For $C_{19}H_{18}N_2O_2$ (M + H) 307.1447, Found: 307.1441.

***N*-(8-Butoxynaphthalen-1-yl)picolinamide (3ai)**: purified by analytical TLC on silica gel with petroleum ether/EtOAc (3:1) as an eluent R_f = 0.55; white solid (53.8mg, 84%), mp 109-111°C. ^{1}H NMR (400 MHz, $CDCl_3$): δ 12.72 (s, 1H), 8.98 (dd, *J* = 7.7, 1.2 Hz, 1H), 8.68-8.65 (m, 1H), 8.36 (d, *J* = 7.8 Hz, 1H), 7.92 (td, *J* = 7.7, 1.7 Hz, 1H), 7.58-7.56 (m, 1H), 7.52-7.47 (m, 2H), 7.45-7.43 (m, 1H), 7.35 (t, *J* = 8.0 Hz, 1H),6.94 (d, *J* = 7.1 Hz, 1H), 4.33 (t, *J* = 6.9 Hz, 2H), 2.14-2.07 (m, 2H), 1.53-1.43 (m, 2H), 0.92 (t, *J* = 7.4 Hz, 3H). ^{13}C NMR (101 MHz, $CDCl_3$): δ 162.8, 155.7, 151.2, 147.7, 137.4, 136.5, 135.0, 126.6, 126.1, 125.6, 124.0, 122.8, 121.8, 117.2, 117.1, 106.8, 69.7, 31.0, 19.3,

13.9.HRMS (positive ESI) Calcd. For $C_{20}H_{20}N_2O_2$ (M + H) 321.1603, Found: 321.1598.

***N*-(8-(Pentyloxy)naphthalen-1-yl)picolinamide (3aj)**: purified by analytical TLC on silica gel with petroleum ether/EtOAc (3:1) as an eluent R_f = 0.51; white solid (52.2mg, 78%), mp 106-110°C.^{1}H NMR (400 MHz, $CDCl_3$): δ 12.72 (s, 1H), 8.98 (dd, *J* = 7.8, 1.3 Hz, 1H), 8.65-8.64 (m, 1H), 8.36 (dt, *J* = 7.9, 0.9 Hz, 1H), 7.91 (td, *J* = 7.8, 1.7 Hz, 1H), 7.58-7.56 (m, 1H), 7.52-7.46 (m, 2H), 7.44-7.42 (m, 1H), 7.34 (t, *J* = 8.0 Hz, 1H), 6.92 (d, *J* = 7.1 Hz, 1H), 4.31 (t, *J* = 7.0 Hz, 2H), 2.15-2.08 (m, 2H), 1.45-1.27 (m, 4H), 0.85 (t, *J* = 7.2 Hz, 3H). ^{13}C NMR (101 MHz, $CDCl_3$): δ 162.8, 155.7, 151.2, 147.6, 137.4, 136.5, 135.0, 126.6, 126.1, 125.6, 124.0, 122.8, 121.8, 117.2, 117.1, 106.8, 77.4, 77.1, 76.7, 70.0, 28.8, 28.3, 22.5, 14.1.HRMS (positive ESI) Calcd. For $C_{21}H_{22}N_2O_2$ (M + H) 335.1760, Found: 335.1754.

***N*-(8-(Hexyloxy)naphthalen-1-yl)picolinamide (3ak)**: purified by analytical TLC on silica gel with petroleum ether/EtOAc (3:1) as an eluent R_f = 0.57;brownsolid (55.8mg, 80%), mp 78-81°C.^{1}H NMR (400 MHz, $CDCl_3$): δ 12.72 (s, 1H), 8.98 (dd, *J* = 7.7, 1.2 Hz, 1H), 8.66-8.65 (m, 1H), 8.36 (d, *J* = 7.8 Hz, 1H), 7.91 (td, *J* = 7.7, 1.7 Hz, 1H), 7.58-7.56 (m, 1H), 7.52-7.46 (m, 1H) 7.44-7.42 (m, 1H) 7.37-7.33 (m, 1H) 6.92 (d, *J* = 7.2 Hz, 1H), 4.31 (t, *J* = 7.0 Hz, 2H), 2.14-2.07 (m, 2H), 1.46-1.39 (m, 2H), 1.31-1.17 (m, 4H), 0.82 (t, *J* = 7.2, 4.9 Hz, 3H). ^{13}C NMR (101 MHz, $CDCl_3$): δ 162.8, 155.7, 151.2, 147.7, 137.4, 136.5, 135.0, 126.6, 126.1, 125.6, 124.0, 122.8, 121.8, 117.2, 117.1, 106.8, 70.0, 31.6, 29.0, 25.8, 22.6, 14.0.HRMS (positive ESI) Calcd. For $C_{22}H_{24}N_2O_2$ (M + H) 349.1916, Found: 349.1911.

***N*-(8-Isobutoxynaphthalen-1-yl)picolinamide (3al)**: purified by analytical TLC on silica gel with petroleum ether/EtOAc (5:1) as an eluent R_f = 0.55; slight yellow solid (57.0mg, 89%),mp 81-84 °C.^{1}H NMR (400 MHz, $CDCl_3$): δ 12.72 (s, 1H), 8.99 (dd, *J* = 7.7, 1.3 Hz, 1H), 8.63-8.61 (m, 1H), 8.36 (dt, *J* = 7.9, 1.0 Hz, 1H), 7.91 (td, *J* = 7.7, 1.7 Hz, 1H), 7.58-7.56 (m, 1H), 7.53-7.42 (m, 3H), 7.35 (t, *J* = 7.9 Hz, 1H), 6.91 (d, *J* = 7.6 Hz, 1H), 4.09 (d, *J* = 7.1Hz, 2H), 2.69-2.56 (m, 1H), 1.03 (d, *J* = 6.6 Hz, 6H). ^{13}C NMR (101 MHz, $CDCl_3$): δ 162.8, 155.7, 151.2, 147.6, 137.4, 136.5, 135.0, 126.6, 126.1, 125.6, 124.1, 122.8, 121.8, 117.3, 117.1, 106.8, 76.4, 27.3, 19.6.HRMS (positive ESI) Calcd. For $C_{20}H_{20}N_2O_2$ (M + H) 321.1603, Found: 321.1598.

***N*-(8-(2-Methylbutoxy)naphthalen-1-yl)picolinamide (3am)**: purified by analytical TLC on silica gel with petroleum ether/EtOAc (3:1) as an eluent R_f = 0.57;slight yellow oil (44.1mg, 66%).^{1}H NMR (400 MHz, $CDCl_3$): δ 12.71 (s, 1H), 8.98 (dd, *J* = 7.7, 1.3 Hz, 1H), 8.65-8.63 (m, 1H), 8.37-8.35 (m, 1H), 7.91 (td, *J* = 7.7, 1.7 Hz, 1H), 7.59-7.56 (m, 1H), 7.53-7.43 (m, 3H), 7.35 (t, *J* = 7.9 Hz, 1H), 6.92 (d, *J* = 7.1 Hz, 1H), 4.25-4.21 (m, 1H), 4.08-4.04 (m, 1H), 2.50-2.37 (m, 1H), 1.63-1.53 (m, 1H), 1.32-1.20 (m, 1H), 1.00 (d, *J* = 6.6 Hz, 3H), 0.91 (t, *J* = 7.4 Hz, 3H). ^{13}C NMR (101 MHz, $CDCl_3$): δ 162.8, 155.8, 151.2, 147.7, 137.4, 136.5, 135.0, 126.6, 126.1, 125.6, 124.1, 122.8, 121.8,

117.3, 117.1, 106.8, 75.2, 33.5, 26.3, 16.6, 11.1.HRMS (positive ESI) Calcd. For $C_{17}H_{14}N_2O_2$ (M + H) 335.1760, Found: 335.1756.

***N*-(8-(Isopentyloxy)naphthalen-1-yl)picolinamide (3an)**: purified by analytical TLC on silica gel with petroleum ether/EtOAc (3:1) as an eluent R_f = 0.52;white solid (54.8mg, 82%), mp 101-104 °C.^{1}H NMR (400 MHz, $CDCl_3$): δ 12.69 (s, 1H), 8.98 (dd, *J* = 7.7, 1.2 Hz, 1H), 8.69-8.67(m, 1H), 8.36 (d, *J* = 7.9 Hz, 1H), 7.92 (td, *J* = 7.7, 1.7 Hz, 1H), 7.58-7.53(m, 1H), 7.52-7.47(m, 2H), 7.45-7.42 (m, 1H), 7.36 (t, *J* = 7.9 Hz, 1H), 6.94 (d, *J* = 7.2 Hz, 1H), 4.35 (t, *J* = 7.3 Hz, 2H), 2.04 (q, *J* = 7.3 Hz 2H), 1.79 (p, *J* = 6.7 Hz, 1H), 0.94 (d, *J* = 6.6 Hz, 6H). ^{13}C NMR (101 MHz, $CDCl_3$): δ 162.8, 155.7, 151.2, 147.7, 137.5, 136.5, 135.0, 126.6, 126.1, 125.6, 124.0, 122.8, 121.8, 117.2, 117.1, 106.8, 68.6, 37.6, 25.4, 22.7.HRMS (positive ESI) Calcd. For $C_{21}H_{22}N_2O_2$ (M + H) 335.1760, Found: 335.1754.

***N*-(8-(Cyclopropylmethoxy)naphthalen-1-yl)picolinamide (3ao)**: purified by analytical TLC on silica gel with petroleum ether/EtOAc (3:1) as an eluent Rf = 0.45;gray solid (28.7mg, 45%), mp 102-105°C.^{1}H NMR (400 MHz, $CDCl_3$): δ 12.95 (s, 1H), 8.98 (dd, *J* = 7.7, 1.3 Hz, 1H), 8.56-8.54(m, 1H), 8.37-8.33 (m, 1H), 7.90 (td, *J* = 7.7, 1.7 Hz, 1H), 7.58-7.56(m, 1H), 7.52-7.49(m, 1H), 7.46-7.43 (m, 2H), 7.34 (dd, *J* = 14.1, 6.1 Hz, 1H), 6.94 (dd, *J* = 7.7, 0.7 Hz, 1H), 4.20 (d, *J* = 7.1 Hz, 2H), 1.65-1.56 (m, 1H), 0.59-0.54 (m, 2H), 0.42-0.37 (m, 2H). ^{13}C NMR (101 MHz, $CDCl_3$): δ 162.7, 155.6, 151.3, 147.6, 137.4, 136.5, 135.1, 126.6, 126.1, 125.6, 124.0, 122.8, 121.9, 117.3, 117.1, 107.5, 74.8, 10.3, 3.9.HRMS (positive ESI) Calcd. For $C_{20}H_{18}N_2O_2$ (M + H) 319.1447, Found: 319.1441.

***N*-(8-(Cyclohexylmethoxy)naphthalen-1-yl)picolinamide (3ap)**: purified by analytical TLC on silica gel with petroleum ether/EtOAc (3:1) as an eluent R_f = 0.59;light yellow solid (33.2mg, 46%), mp 99-101°C.^{1}H NMR (400 MHz, $CDCl_3$): δ 12.74 (s, 1H), 8.97 (dd, *J* = 7.7, 1.2 Hz, 1H), 8.72-8.70 (m, 1H), 8.36 (d, *J* = 7.8 Hz, 1H), 7.92 (td, *J* = 7.7, 1.7 Hz, 1H), 7.58-7.56 (m, 1H) 7.52-7.47 (m, 2H), 7.44-7.42 (m, 1H), 7.35 (t, *J* = 7.9 Hz, 1H), 6.91 (d, *J* = 7.2 Hz, 1H), 4.14 (d, *J* = 6.9 Hz, 2H), 2.34-2.28 (m, 1H), 1.92-1.90 (m, 2H), 1.66-1.63 (m, 3H), 1.18-0.91 (m, 5H). ^{13}C NMR (101 MHz, $CDCl_3$): δ 162.7, 155.8, 151.3, 147.9, 137.5, 136.5, 135.0, 126.6, 126.0, 125.6, 124.0, 122.8, 121.7, 117.1, 106.9, 77.4, 77.1, 76.7, 75.5, 36.2, 30.0, 26.4, 25.5.HRMS (positive ESI) Calcd. For $C_{23}H_{24}N_2O_2$ (M + H) 361.1916, Found: 361.1911.

N-(8-(Adamantanylmethoxy)naphthalen-1-yl)picolinamide (3aq): purified by analytical TLC on silica gel with petroleum ether/EtOAc (3:1) as an eluent Rf = 0.57; white solid (46.2mg, 56%), mp 166-175 °C. 1H NMR (400 MHz, CDCl3): δ 12.41 (s, 1H), 8.89 (dd, J = 7.7, 1.2 Hz, 1H), 8.67-8.65 (m, 1H), 8.36 (d, J = 7.8 Hz, 1H), 7.91 (td, J = 7.7, 1.7 Hz, 1H), 7.58-7.56 (m, 1H), 7.51-7.46 (m, 2H), 7.44-7.41 (m, 1H), 7.34 (t, J = 7.9 Hz, 1H), 7.04 (d, J = 7.2 Hz, 1H), 4.08 (s, 2H), 1.89 (s, 3H), 1.67-1.66 (m, 7H), 1.62 (s, 2H), 1.48 (d, J = 11.5 Hz, 3H). 13C NMR (101 MHz, CDCl3): δ 162.9, 155.1, 151.2, 147.7, 137.4, 136.7, 135.1, 126.5, 126.1, 125.7, 124.1, 122.9, 121.7, 117.8, 117.2, 108.2, 76.7,

38.3, 20.0, 19.2, 14.0. HRMS (positive ESI) Calcd. For C27H28N2O2 (M + H) 413.2229, Found: 413.2224.

***N*-(8-(3-Methoxypropoxy)naphthalen-1-yl)picolinamide (3ar)**: purified by analytical TLC on silica gel with petroleum ether/EtOAc (1:1) as an eluent R_f = 0. 38;white solid (45.7mg, 68%), mp 136-139^oC.^1H NMR (400 MHz, $CDCl_3$): δ 12.69 (s, 1H), 8.99 (dd, *J* = 7.7, 1.2 Hz, 1H), 8.68-8.67 (m, 1H), 8.36 (d, *J* = 7.8 Hz, 1H), 7.92 (td, *J* = 7.7, 1.7 Hz, 1H), 7.58-7.56 (m, 1H), 7.53-7.46 (m, 2H), 7.45-7.42 (m, 1H) 7.36 (t, *J* = 7.9 Hz, 1H), 6.96 (d, *J* = 7.2 Hz, 1H), 4.44 (t, *J* = 6.3 Hz, 2H), 3.54 (t, *J* = 6.2 Hz, 2H), 3.23 (s, 3H), 2.42 (p, *J* = 6.2 Hz, 2H).^{13}C NMR (101 MHz, $CDCl_3$): δ 162.7, 155.6, 151.1, 147.8, 137.5, 136.5, 134.9, 126.6, 126.1, 125.7, 124.1, 122.8, 122.0, 117.3, 117.0, 106.9, 69.5, 66.8, 58.7, 29.3.HRMS (positive ESI) Calcd. For $C_{20}H_{20}N_2O_2$ (M + H) 337.1552, Found: 337.1547.

***N*-(8-((4-Bromobenzyl)oxy)naphthalen-1-yl)picolinamide (3as)**: purified by analytical TLC on silica gel with petroleum ether/EtOAc (3:1) as an eluent R_f = 0.40;white solid (60.7mg, 70%), mp 127-131 ^oC.^1H NMR (400 MHz, $CDCl_3$): δ 12.93 (s, 1H), 8.93 (dd, *J* = 7.5, 1.4 Hz, 1H), 8.27 (dd, *J* = 7.8, 0.9 Hz, 1H), 8.15-8.13(m, 1H), 7.83 (td, *J* = 7.7, 1.7 Hz, 1H), 7.55-7.45 (m, 2H), 7.41-7.39 (m, 1H), 7.35-7.30 (m, 5H), 7.24-7.22(m, 1H), 6.82 (d, *J* = 7.1 Hz, 1H), 5.39 (s, 2H).^{13}C NMR (101 MHz, $CDCl_3$): δ 162.5, 154.9, 150.7, 147.8, 137.4, 136.5, 135.7, 134.9, 131.7, 129.2, 126.8, 126.0, 125.5, 124.0, 122.5, 122.1, 117.2, 117.1, 108.0, 70.8.HRMS (positive ESI) Calcd. For $C_{23}H_{17}BrN_2O_2$ (M + H) 433.0552, Found: 433.0546.

***N*-(8-Isopropoxynaphthalen-1-yl)picolinamide (3at)**: purified by analytical TLC on silica gel with petroleum ether/EtOAc (3:1) as an eluent R_f = 0.48;light yellow solid (35.5mg, 58%), mp 81-83^oC.^1H NMR (400 MHz, $CDCl_3$) δ 12.71 (s, 1H), 9.01 (dd, *J* = 7.7, 1.3 Hz, 1H), 8.68-8.67(m, 1H), 8.36 (dt, *J* = 7.9, 1.0 Hz, 1H), 7.90 (td, *J* = 7.7, 1.7 Hz, 1H), 7.58-7.56(m, 1H), 7.52-7.46(m, 2H), 7.43-7.42 (m, 1H) 7.35 (t, *J* = 7.9 Hz, 1H), 6.98 (d, *J* = 7.6 Hz, 1H), 4.96-4.86 (m, 1H), 1.59 (d, *J* = 6.1 Hz, 6H). ^{13}C NMR (101 MHz, $CDCl_3$): δ 162.9, 154.7, 151.2, 147.7, 137.4, 136.6, 135.1, 126.5, 126.1, 125.7, 124.1, 122.8, 121.8, 117.8, 117.1, 108.5, 72.9, 22.0.HRMS (positive ESI) Calcd. For $C_{19}H_{18}N_2O_2$ (M + H) 307.1447, Found: 307.1441.

***N*-(8-(*sec*-Butoxy)naphthalen-1-yl)picolinimidamide(3au)**: purified by analytical TLC on silica gel with petroleum ether/EtOAc (3:1) as an eluent R_f = 0.53;yellow oil (41.7mg, 65%).^1H NMR (400 MHz, $CDCl_3$): δ 12.72 (s, 1H), 9.01 (dd, *J* = 7.7, 1.3 Hz, 1H), 8.67-8.65 (m, 1H), 8.36 (dt, *J* = 7.9, 1.0 Hz, 1H), 7.91 (td, *J* = 7.7, 1.7 Hz, 1H), 7.58-7.56 (m, 1H), 7.53-7.46 (m, 2H), 7.44-7.42 (m, 1H), 7.35 (t, *J* = 7.9 Hz, 1H), 6.98 (d, *J* = 7.6 Hz, 1H), 4.710-4.62 (m, 1H), 2.25-2.13 (m, 1H), 1.90-1.79 (m, 1H), 1.54 (d, *J* = 6.1 Hz, 3H), 1.02 (t, *J* = 7.4 Hz, 3H). ^{13}C NMR (101 MHz, $CDCl_3$): δ 162.9, 155.1, 151.2, 147.6, 137.4, 136.7, 135.1, 126.5, 126.1, 125.7, 124.1, 122.8, 121.7, 117.8, 117.2, 108.3, 78.3, 29.0, 19.6, 10.4.HRMS (positive ESI) Calcd. For $C_{20}H_{20}N_2O_2$ (M + H) 321.1603, Found: 321.1598.

***N*-(8-(Pentan-2-yloxy)naphthalen-1-yl)picolinamide (3av)**: purified by analytical TLC on silica gel with petroleum ether/EtOAc (3:1) as an eluent R_f = 0.55;light yellow oil (59.5mg, 89%). ^{1}H NMR (400 MHz, $CDCl_3$): δ 12.71 (s, 1H), 9.01 (dd, *J* = 7.7, 1.3 Hz, 1H), 8.69 -8.67 (m, 1H), 8.36 (dt, *J* = 7.9, 1.0 Hz, 1H), 7.91 (td, *J* = 7.7, 1.7 Hz, 1H), 7.58-7.56 (dm, 1H), 7.53-7.46 (m, 2H), 7.44-7.42 (m, 1H), 7.35 (t, *J* = 7.9 Hz, 1H), 6.97 (d, *J* = 7.6 Hz, 1H), 4.77-4.70 (m, 1H), 2.27-2.16 (m, 1H), 1.80-1.70 (m, 1H), 1.53 (d, *J* = 6.1 Hz, 3H), 1.51-1.38 (m, 2H), 0.88 (t, *J* = 7.4 Hz, 3H). ^{13}C NMR (101 MHz, $CDCl_3$): δ 162.9, 155.1, 151.2, 147.7, 137.4, 136.7, 135.1, 126.5, 126.1, 125.7, 124.1, 122.9, 121.7, 117.8, 117.2, 108.2, 76.7, 38.3, 20.0, 19.2, 14.0. HRMS (positive ESI) Calcd. For C21H22N2O2 (M + H) 335.1760, Found: 335.1754.

NMR spectra

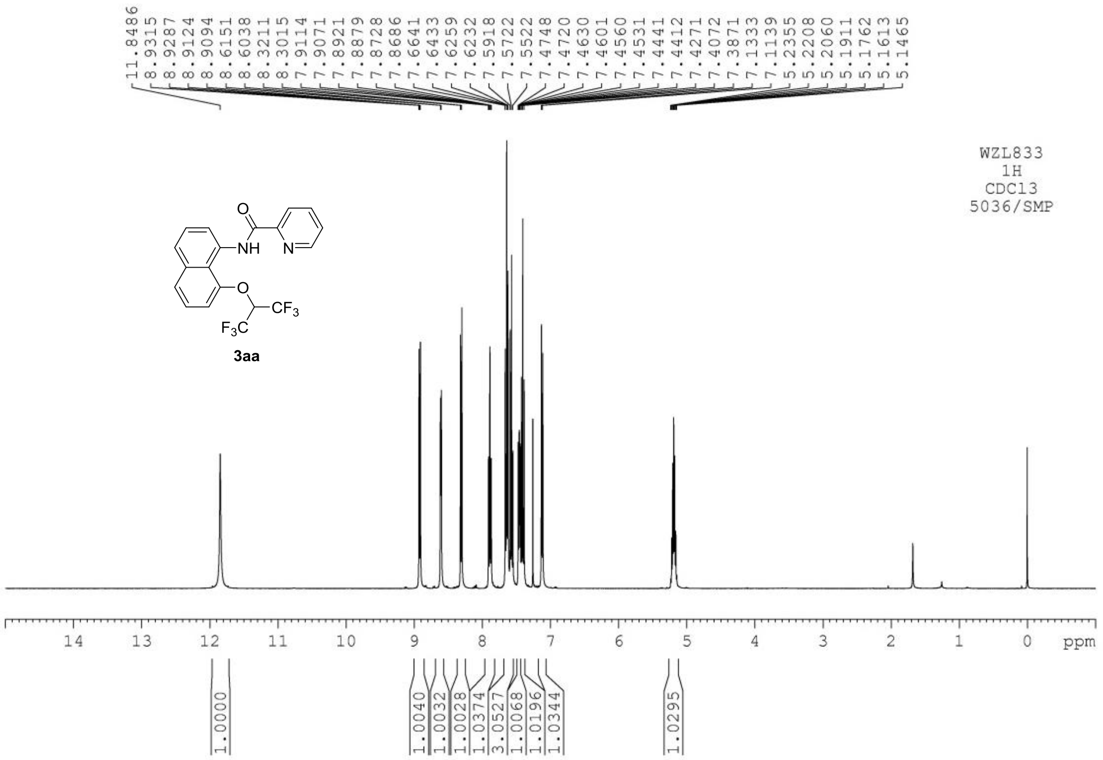

WZL833
1H
CDCl3
5036/SMP
3aa
ppm

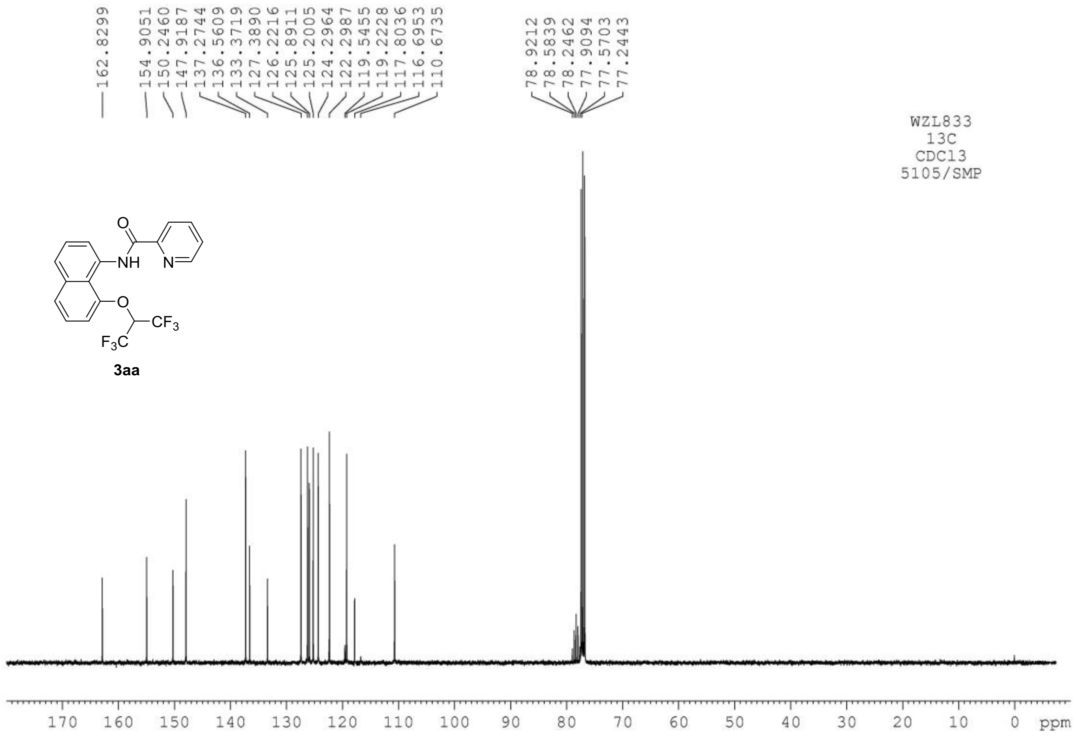

WZL833
13C
CDCl3
5105/SMP
3aa
ppm

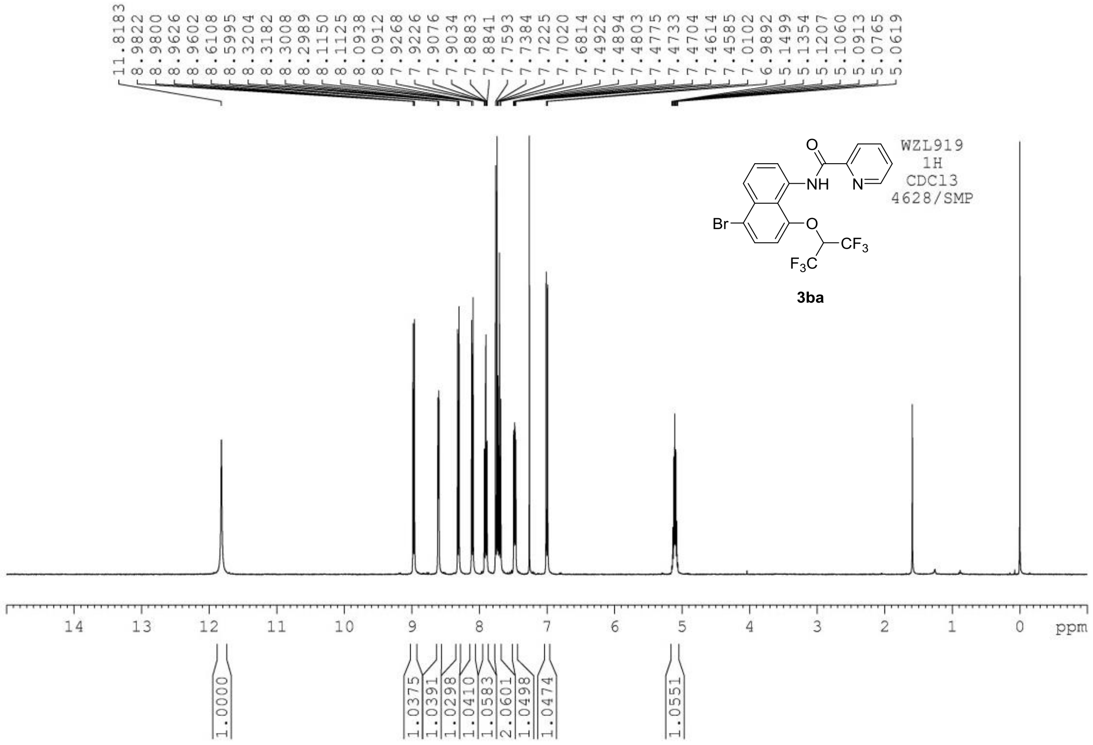

WZL919
1H
CDCl3
4628/SMP
NH
N
O
Br
O
CF3
F3C
3ba
ppm

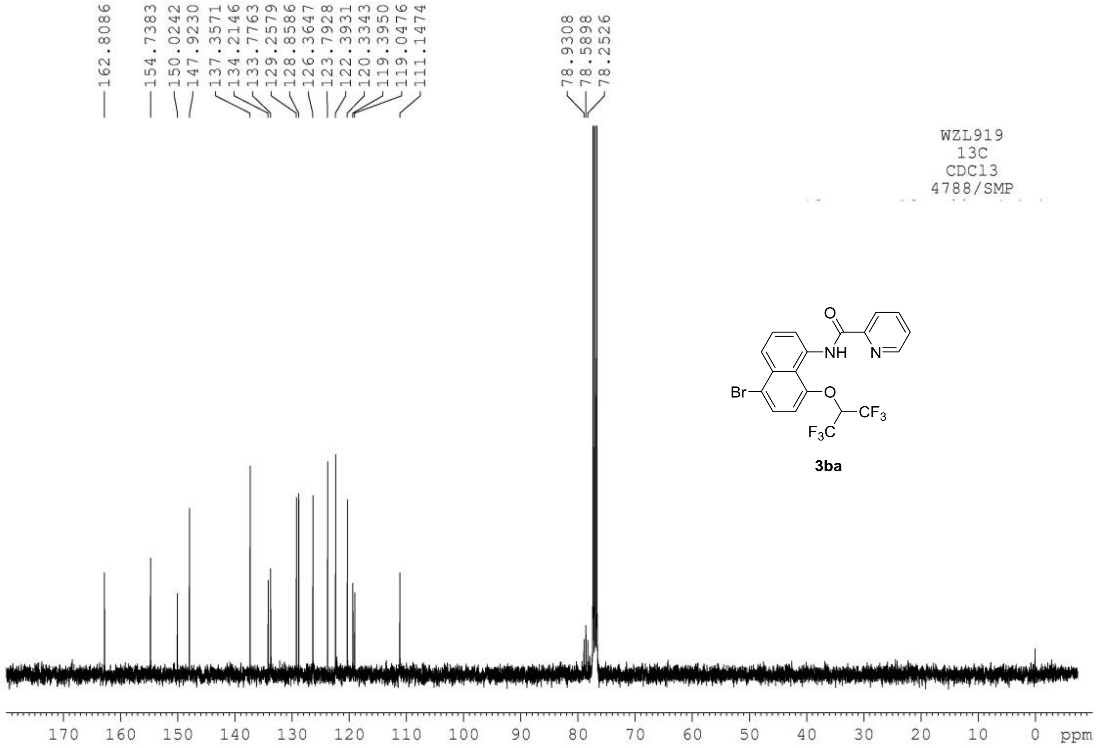

WZL919
13C
CDCl3
4788/SMP
NH
N
O
Br
O
CF3
F3C
3ba
ppm

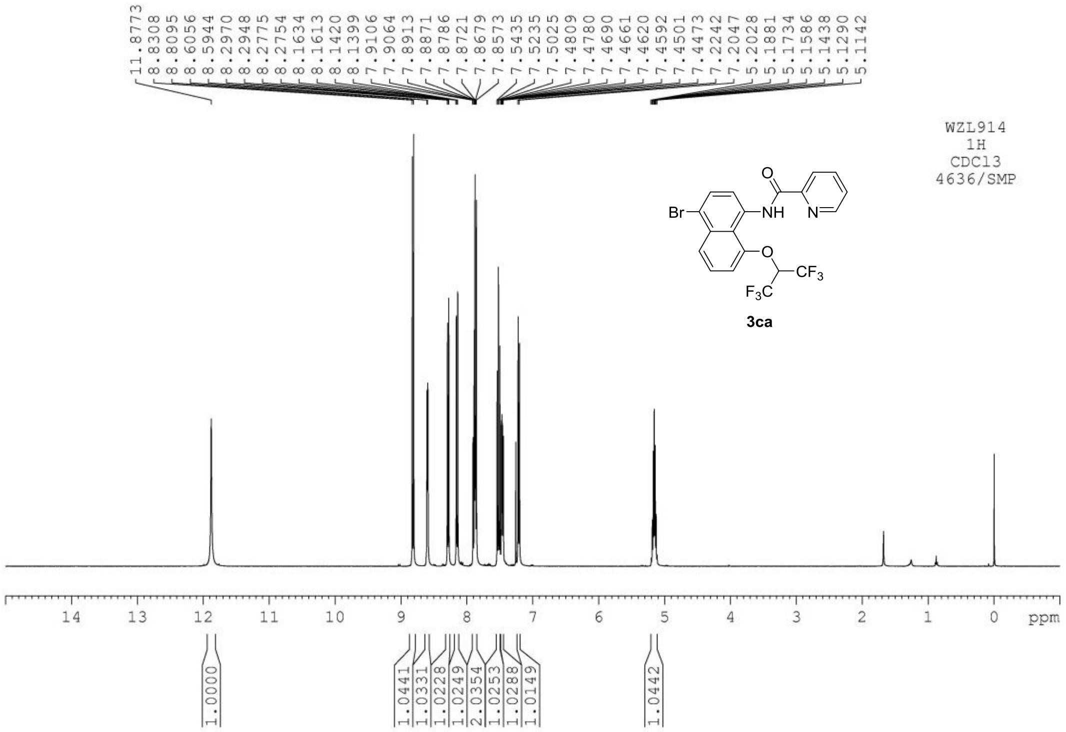
11.8773
8.8308
8.8095
8.6056
8.5944
8.2970
8.2948
8.2775
8.2754
8.1634
8.1613
8.1420
8.1399
7.9106
7.9064
7.8913
7.8871
7.8786
7.8721
7.8679
7.8573
7.5435
7.5235
7.5025
7.4809
7.4780
7.4690
7.4661
7.4620
7.4592
7.4501
7.4473
7.2242
7.2047
7.2028
5.1881
5.1734
5.1586
5.1438
5.1290
5.1142
WZL914
1H
CDCl3
4636/SMP
Br
NH
O
N
O
CF3
F3C
3ca
ppm
1.0000
1.0441
1.0331
1.0228
1.0249
2.0354
1.0253
1.0288
1.0149
1.0442

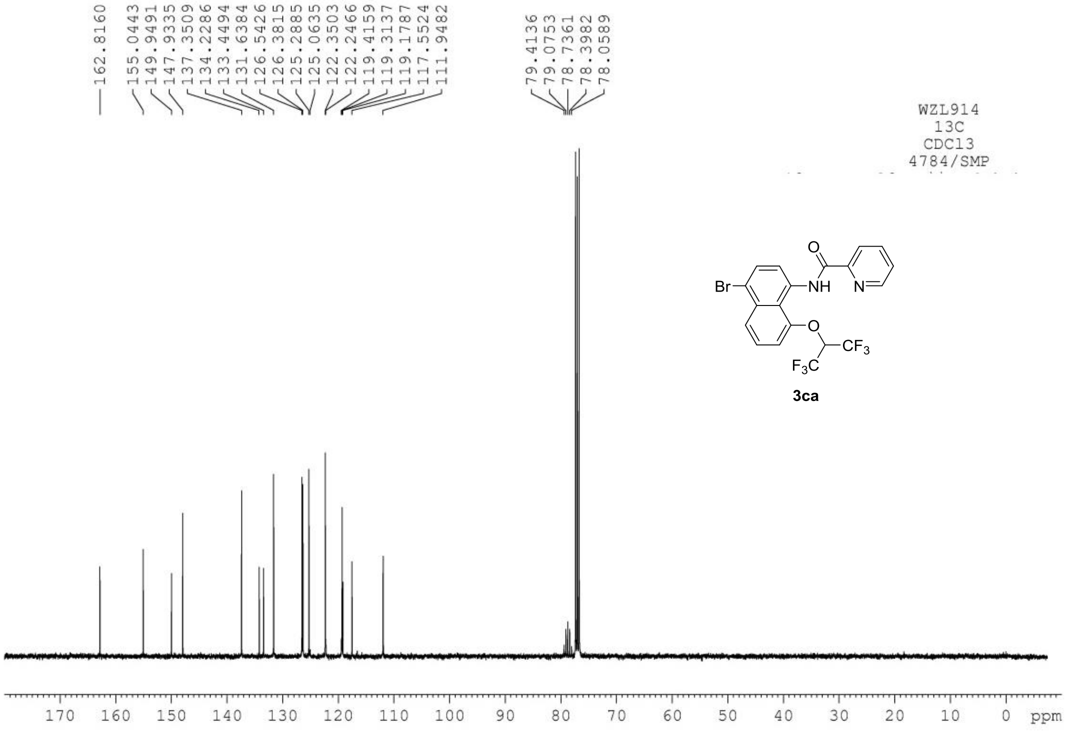
162.8160
155.0443
149.9491
147.9335
137.3509
134.2286
133.4494
131.6384
126.5426
126.3815
125.2885
125.0635
122.3503
122.2466
119.4159
119.3137
119.1787
117.5524
111.9482
79.4136
79.0753
78.7361
78.3982
78.0589
WZL914
13C
CDCl3
4784/SMP
Br
NH
O
N
O
CF3
F3C
3ca
ppm

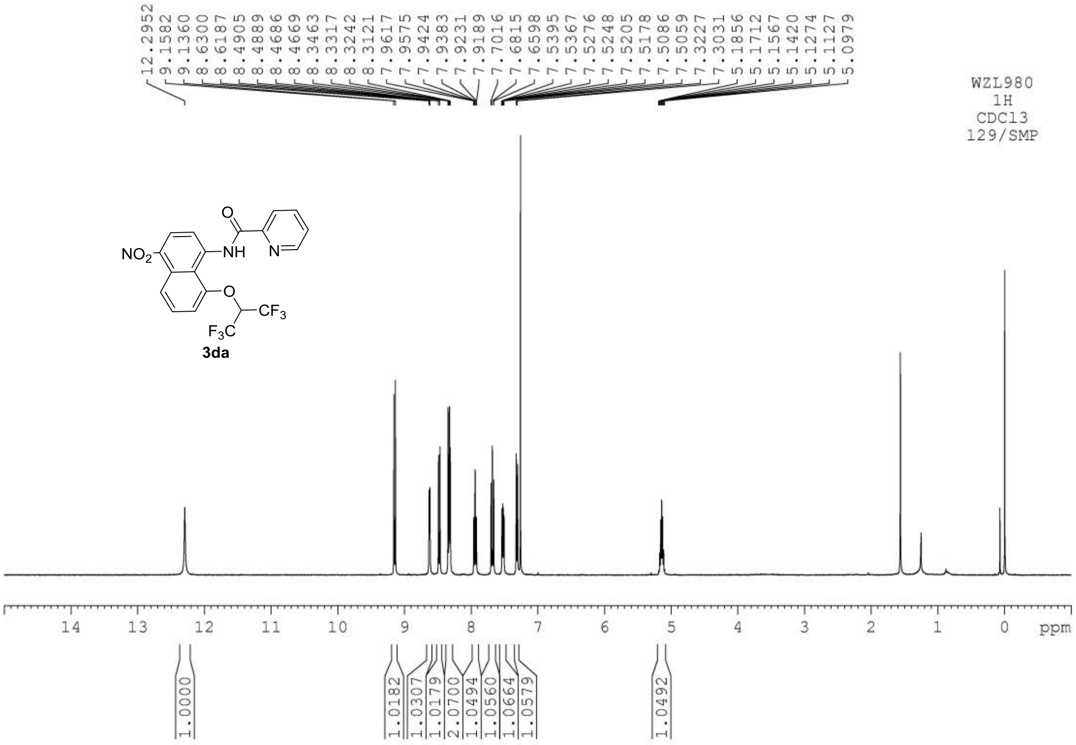
12.2952
9.1582
9.1360
8.6300
8.6187
8.4905
8.4889
8.4686
8.4669
8.3463
8.3317
8.3242
8.3121
7.9617
7.9575
7.9424
7.9383
7.9231
7.9189
7.7016
7.6815
7.6598
7.5395
7.5367
7.5276
7.5248
7.5205
7.5178
7.5086
7.5059
7.3227
7.3031
5.1856
5.1712
5.1567
5.1420
5.1274
5.1127
5.0979
WZL980
1H
CDCl3
129/SMP
O
NO_2
NH
N
O
CF_3
F_3C
3da
14 13 12 11 10 9 8 7 6 5 4 3 2 1 0 ppm
1.0000
1.0182
1.0307
1.0179
2.0700
1.0494
1.0560
1.0664
1.0579
1.0492

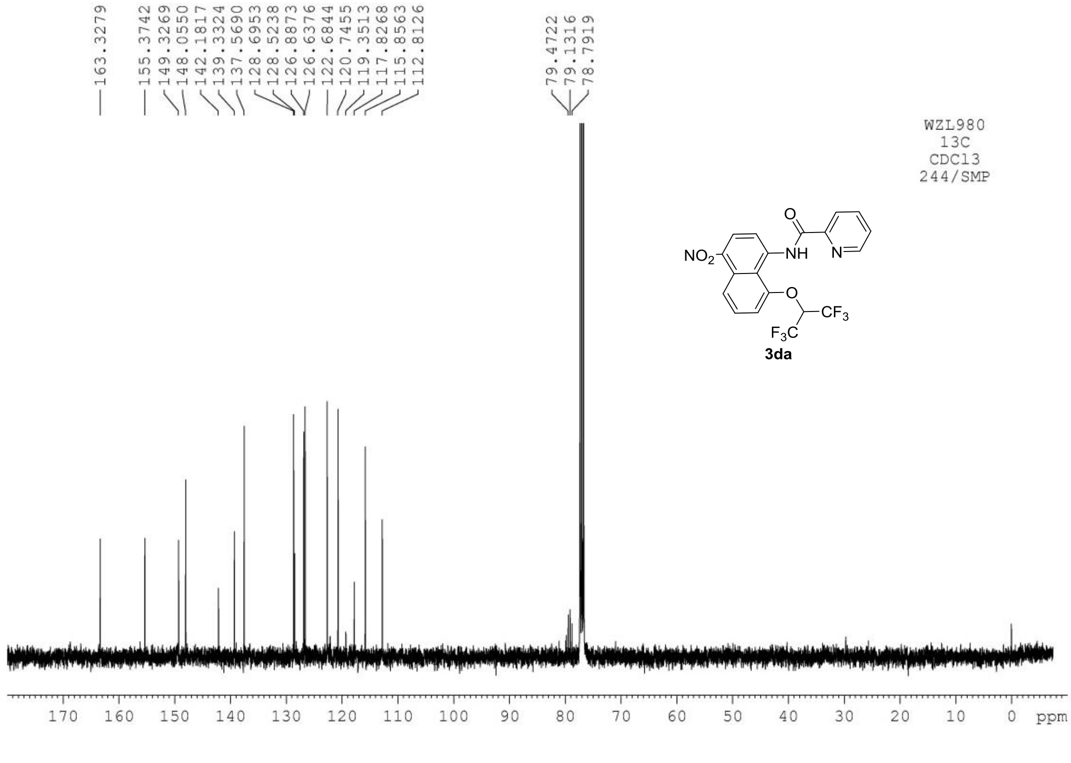
163.3279
155.3742
149.3269
148.0550
142.1817
139.3324
137.5690
128.6953
128.5238
126.8873
126.6376
122.6844
120.7455
119.3513
117.8268
115.8563
112.8126
79.4722
79.1316
78.7919
WZL980
13C
CDCl3
244/SMP
O
NO_2
NH
N
O
CF_3
F_3C
3da
170 160 150 140 130 120 110 100 90 80 70 60 50 40 30 20 10 0 ppm

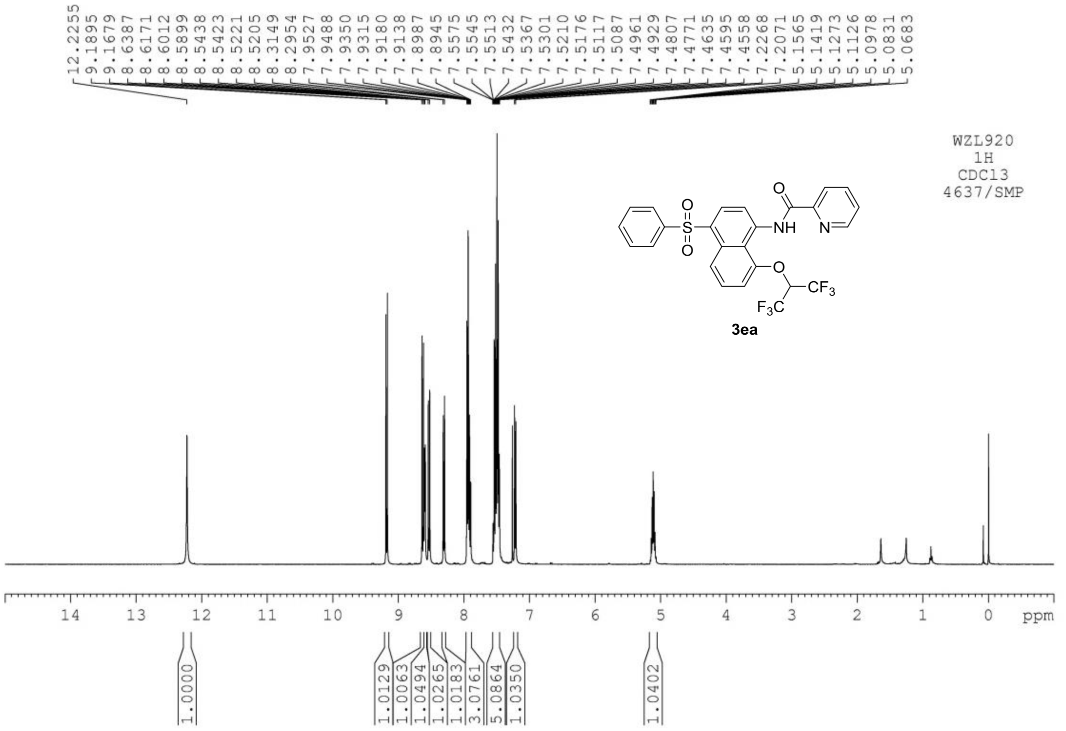
12.2255
9.1895
9.1679
8.6387
8.6171
8.6012
8.5899
8.5438
8.5423
8.5221
8.5205
8.3149
8.2954
7.9527
7.9488
7.9350
7.9315
7.9180
7.9138
7.8987
7.8945
7.5575
7.5545
7.5513
7.5432
7.5367
7.5301
7.5210
7.5176
7.5117
7.5087
7.4961
7.4929
7.4807
7.4771
7.4635
7.4595
7.4558
7.2268
7.2071
5.1565
5.1419
5.1273
5.1126
5.0978
5.0831
5.0683
WZL920
1H
CDCl3
4637/SMP
O
S
O
O
NH
N
O
CF3
F3C
3ea
ppm
1.0000
1.0129
1.0063
1.0494
1.0265
1.0183
3.0761
5.0864
1.0350
1.0402

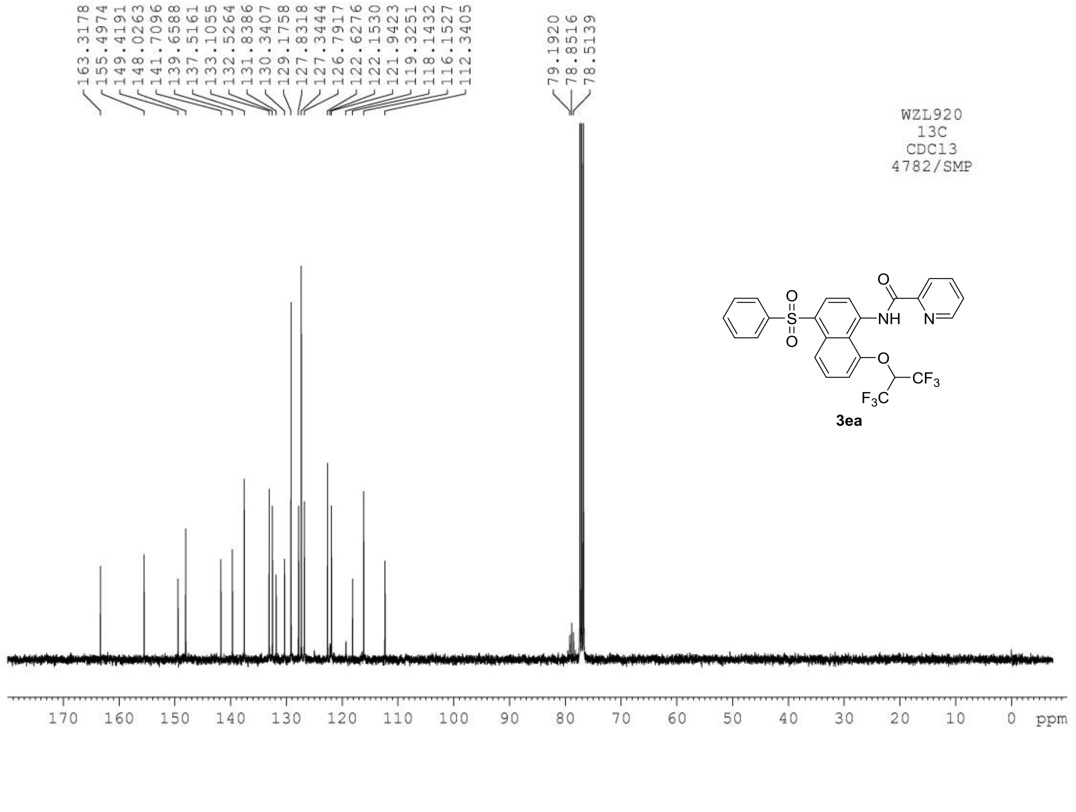
163.3178
155.4974
149.4191
148.0263
141.7096
139.6588
137.5161
133.1055
132.5264
131.8386
130.3407
129.1758
127.8318
127.3444
126.7917
122.6276
122.1530
121.9423
119.3251
118.1432
116.1527
112.3405
79.1920
78.8516
78.5139
WZL920
13C
CDCl3
4782/SMP
O
S
O
O
NH
N
O
CF3
F3C
3ea
ppm

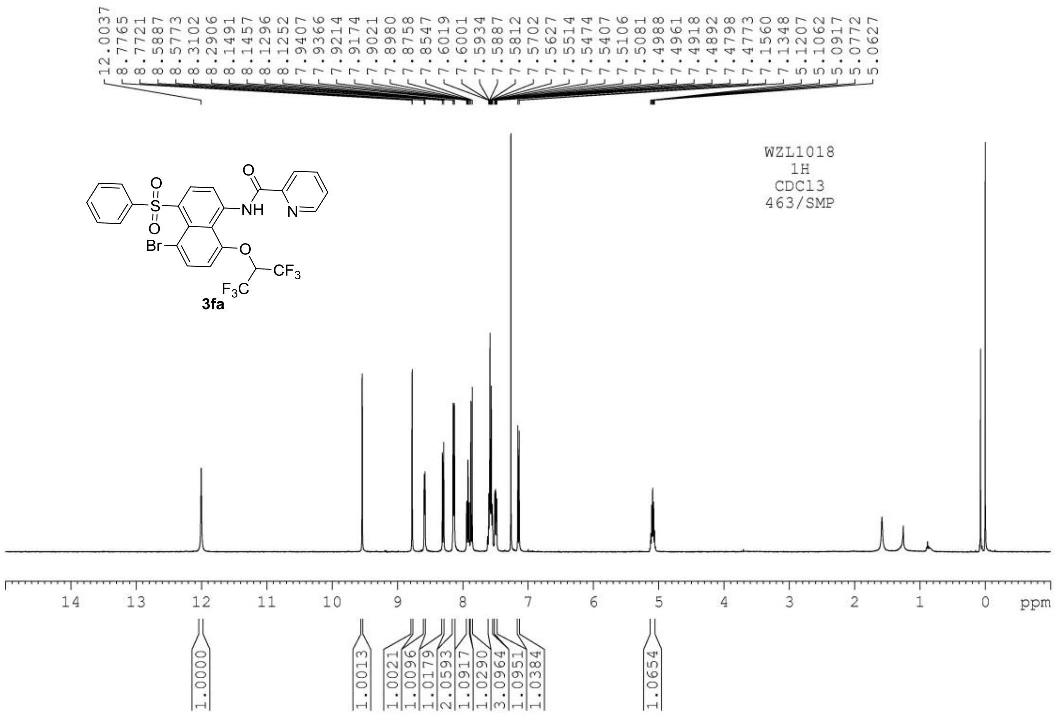
12.0037
8.7765
8.7721
8.5887
8.5773
8.3102
8.2906
8.1491
8.1457
8.1296
8.1252
7.9407
7.9366
7.9214
7.9174
7.9021
7.8980
7.8758
7.8547
7.6019
7.6001
7.5934
7.5887
7.5812
7.5702
7.5627
7.5514
7.5474
7.5407
7.5106
7.5081
7.4988
7.4961
7.4918
7.4892
7.4798
7.4773
7.1560
7.1348
5.1207
5.1062
5.0917
5.0772
5.0627
WZL1018
1H
CDCl3
463/SMP
NH
N
Br
CF3
F3C
3fa
ppm
1.0000
1.0013
1.0021
1.0096
1.0179
2.0593
1.0917
1.0290
3.0964
1.0951
1.0384
1.0654

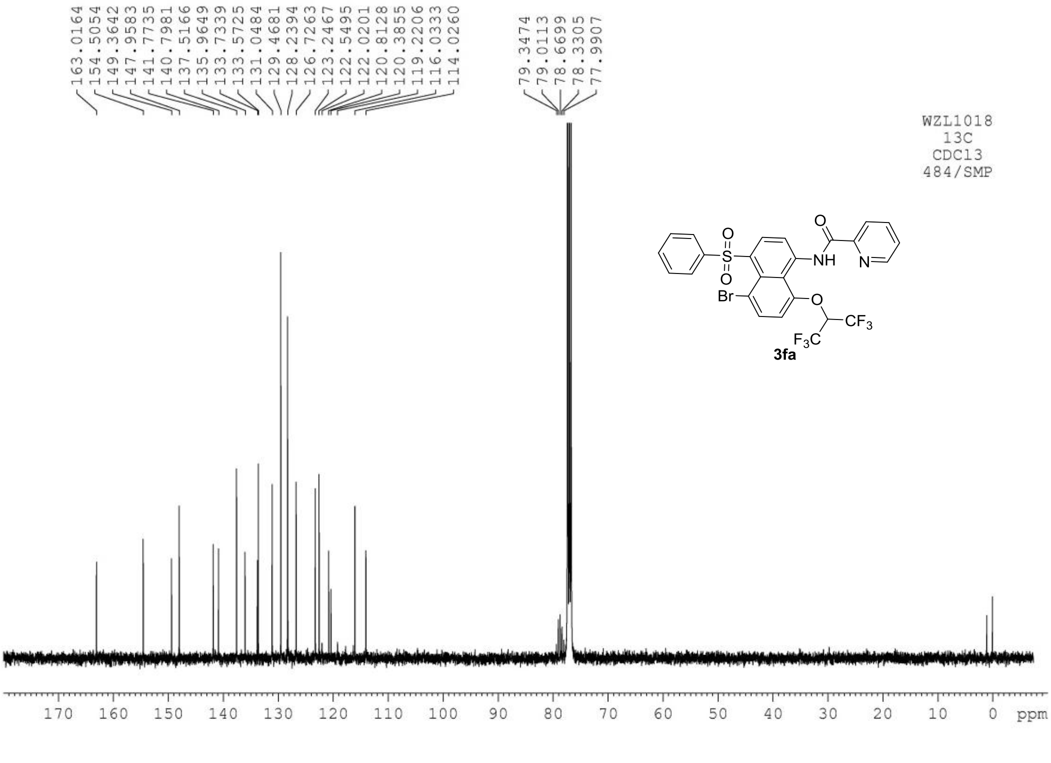
163.0164
154.5054
149.3642
147.9583
141.7735
140.7981
137.5166
135.9649
133.7339
133.5725
131.0484
129.4681
128.2394
126.7263
123.2467
122.5495
122.0201
120.8128
120.3855
119.2206
116.0333
114.0260
79.3474
79.0113
78.6699
78.3305
77.9907
WZL1018
13C
CDCl3
484/SMP
NH
N
Br
CF3
F3C
3fa
ppm

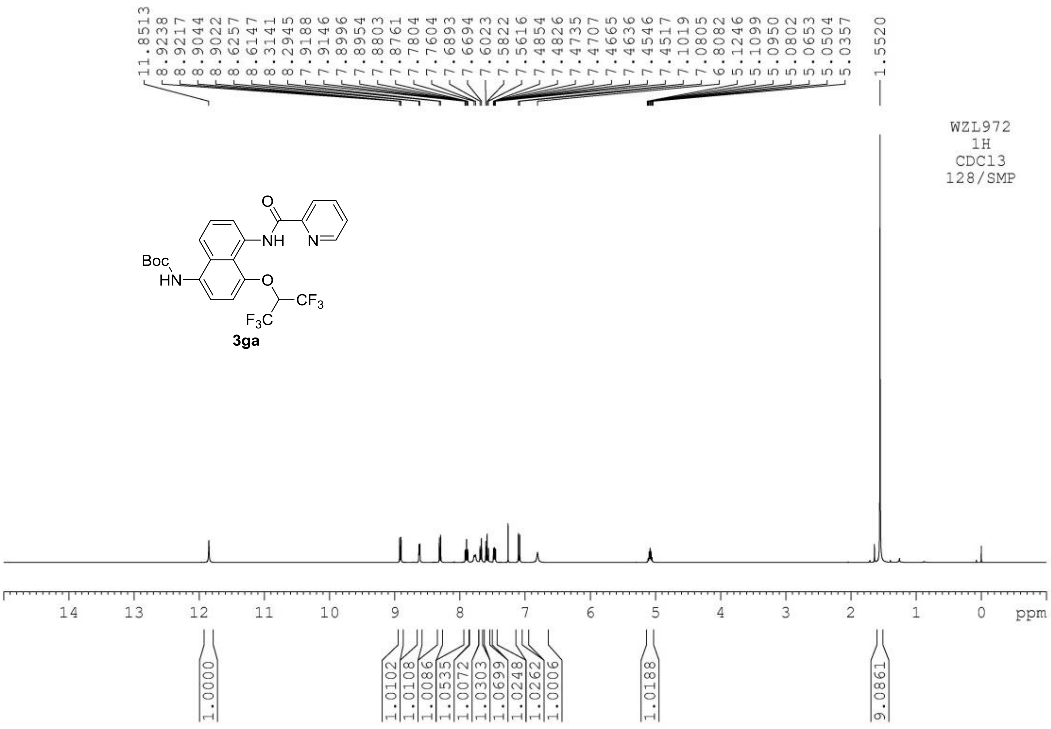
WZL972
1H
CDCl3
128/SMP
3ga
ppm

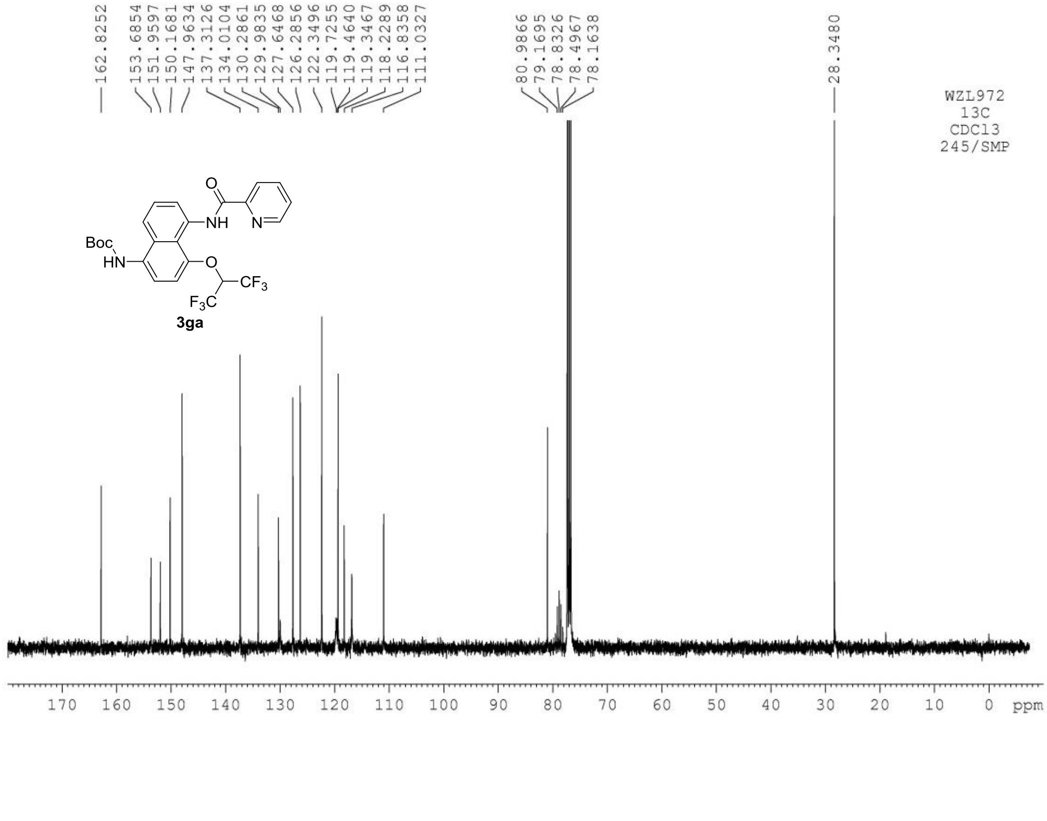
WZL972
13C
CDCl3
245/SMP
3ga
ppm

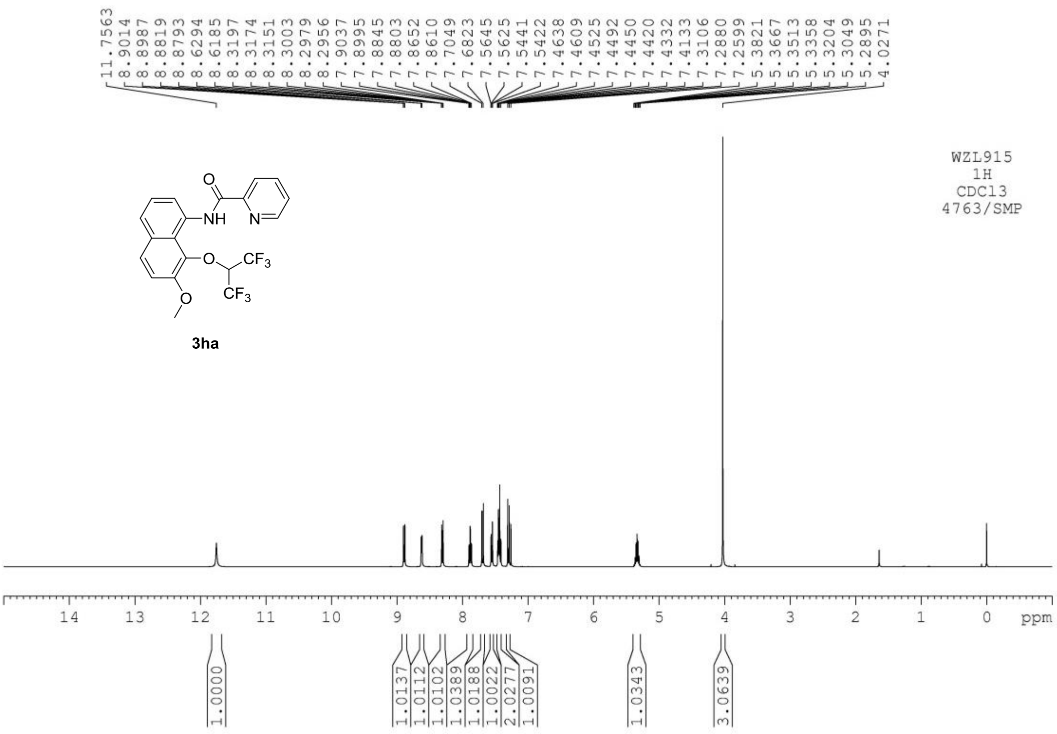
WZL915
1H
CDCl3
4763/SMP
3ha

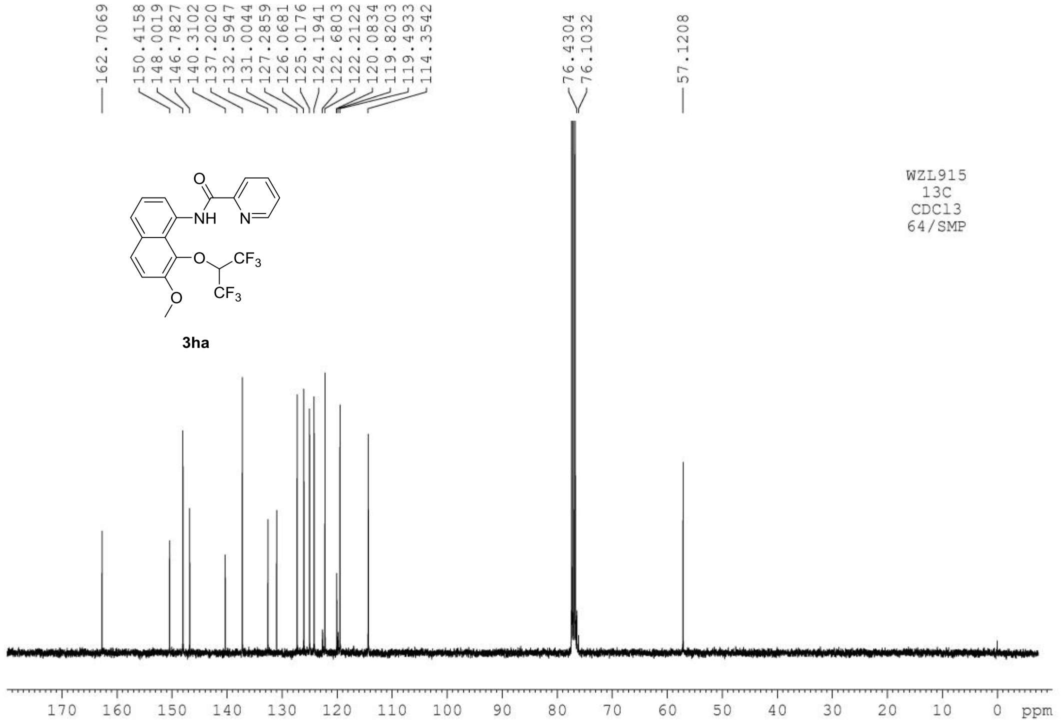
WZL915
13C
CDCl3
64/SMP
3ha

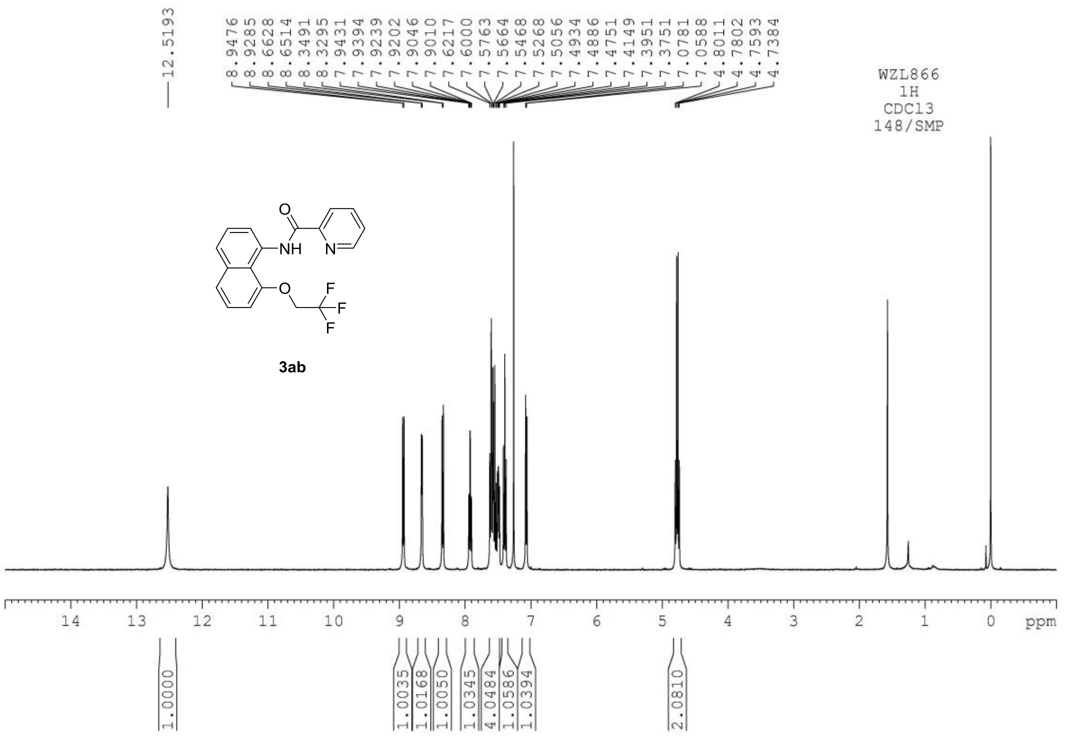
12.5193
8.9476
8.9285
8.6628
8.6514
8.3491
8.3295
7.9431
7.9394
7.9239
7.9202
7.9046
7.9010
7.6217
7.6000
7.5763
7.5664
7.5468
7.5268
7.5056
7.4934
7.4886
7.4751
7.4149
7.3951
7.3751
7.0781
7.0588
4.8011
4.7802
4.7593
4.7384
WZL866
1H
CDCl3
148/SMP
O
NH
N
O
F
F
F
3ab
14 13 12 11 10 9 8 7 6 5 4 3 2 1 0 ppm
1.0000
1.0035
1.0168
1.0050
1.0345
4.0484
1.0586
1.0394
2.0810

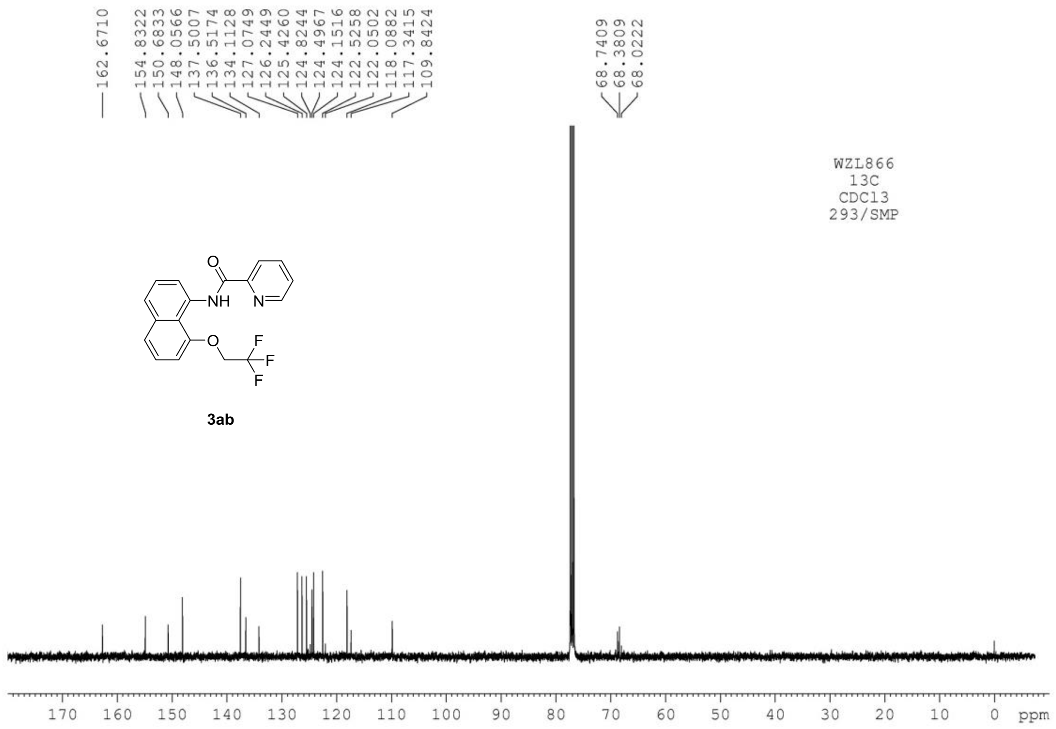
162.6710
154.8322
150.6833
148.0566
137.5007
136.5174
134.1128
127.0749
126.2449
125.4260
124.8244
124.4967
124.1516
122.5258
122.0502
118.0882
117.3415
109.8424
68.7409
68.3809
68.0222
WZL866
13C
CDCl3
293/SMP
O
NH
N
O
F
F
F
3ab
170 160 150 140 130 120 110 100 90 80 70 60 50 40 30 20 10 0 ppm

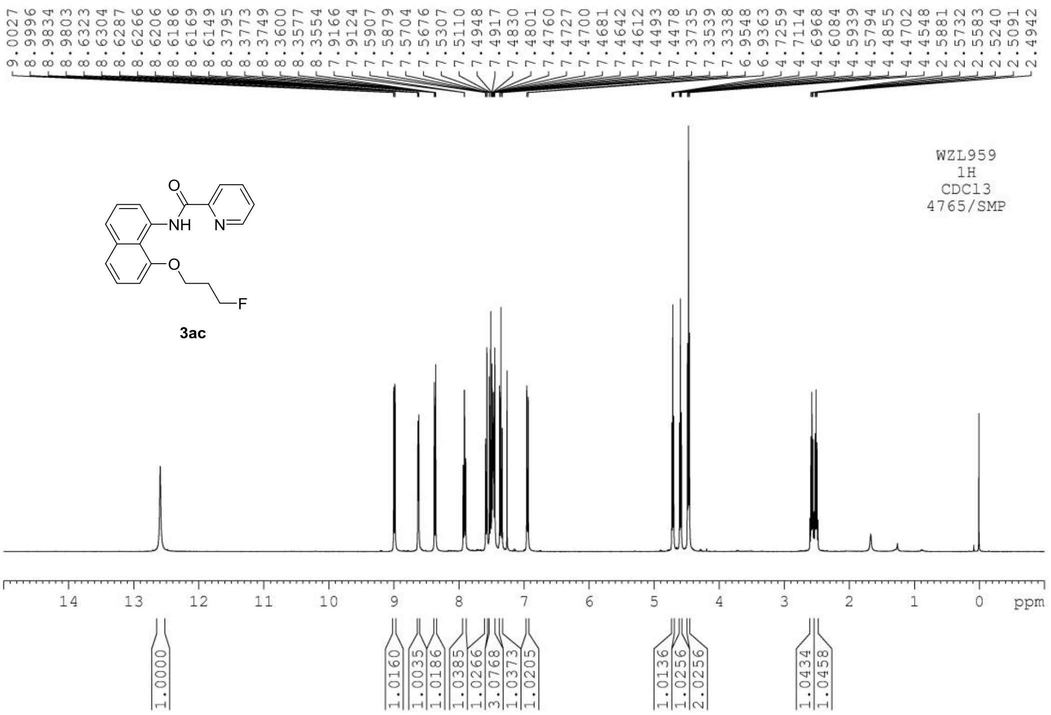
9.0027
8.9996
8.9834
8.9803
8.6323
8.6304
8.6287
8.6266
8.6206
8.6186
8.6169
8.6149
8.3795
8.3773
8.3749
8.3600
8.3577
8.3554
7.9166
7.9124
7.5907
7.5879
7.5704
7.5676
7.5307
7.5110
7.4948
7.4917
7.4830
7.4801
7.4760
7.4727
7.4700
7.4681
7.4642
7.4612
7.4493
7.4478
7.3735
7.3539
7.3338
6.9548
6.9363
4.7259
4.7114
4.6968
4.6084
4.5939
4.5794
4.4855
4.4702
4.4548
2.5881
2.5732
2.5583
2.5240
2.5091
2.4942
WZL959
1H
CDCl3
4765/SMP
O
NH
N
O
F
3ac
ppm
1.0000
1.0160
1.0035
1.0186
1.0385
1.0266
3.0768
1.0373
1.0205
1.0136
1.0256
2.0256
1.0434
1.0458

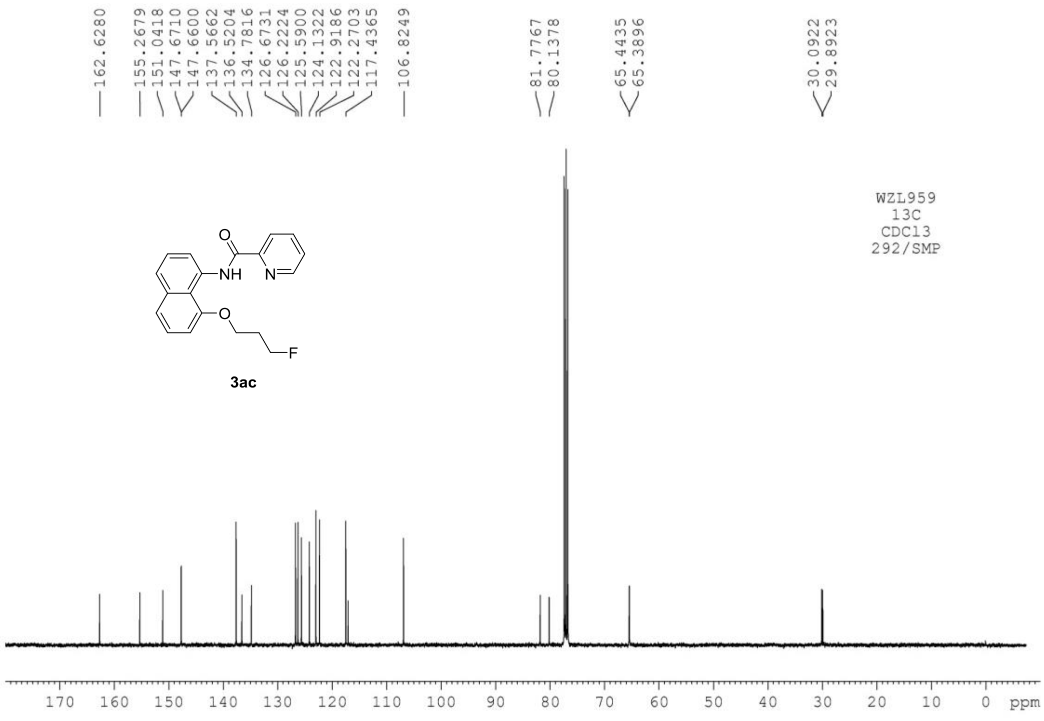
162.6280
155.2679
151.0418
147.6710
147.6600
137.5662
136.5204
134.7816
126.6731
126.2224
125.5900
124.1322
122.9186
122.2703
117.4365
106.8249
81.7767
80.1378
65.4435
65.3896
30.0922
29.8923
WZL959
13C
CDCl3
292/SMP
O
NH
N
O
F
3ac
ppm

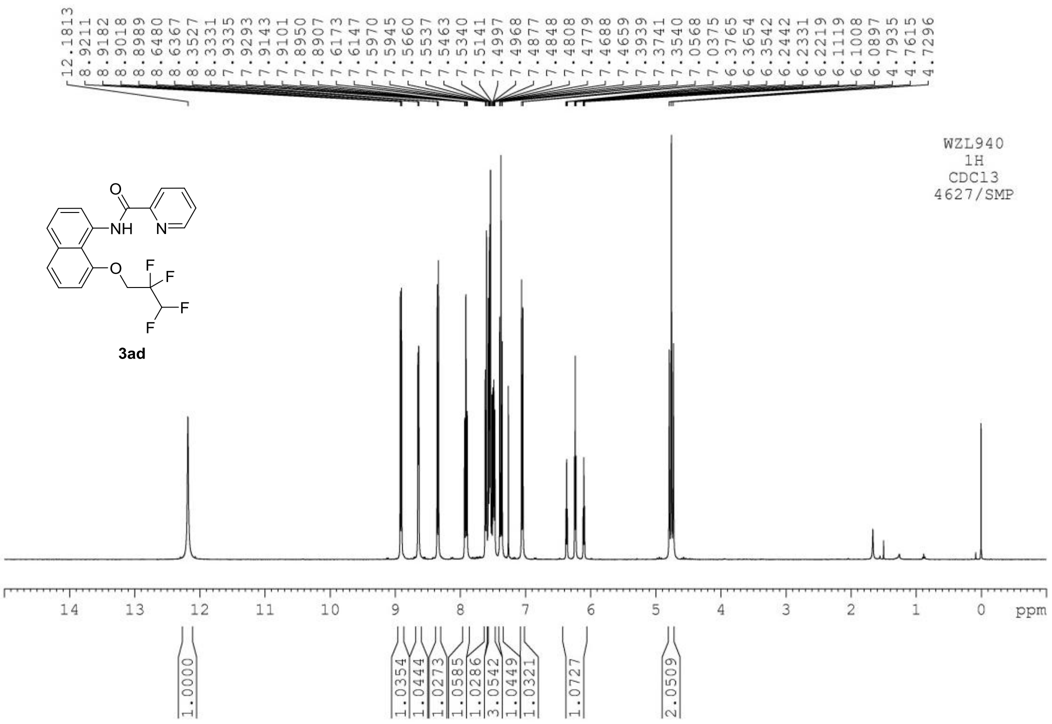
WZL940
1H
CDCl3
4627/SMP
3ad
ppm

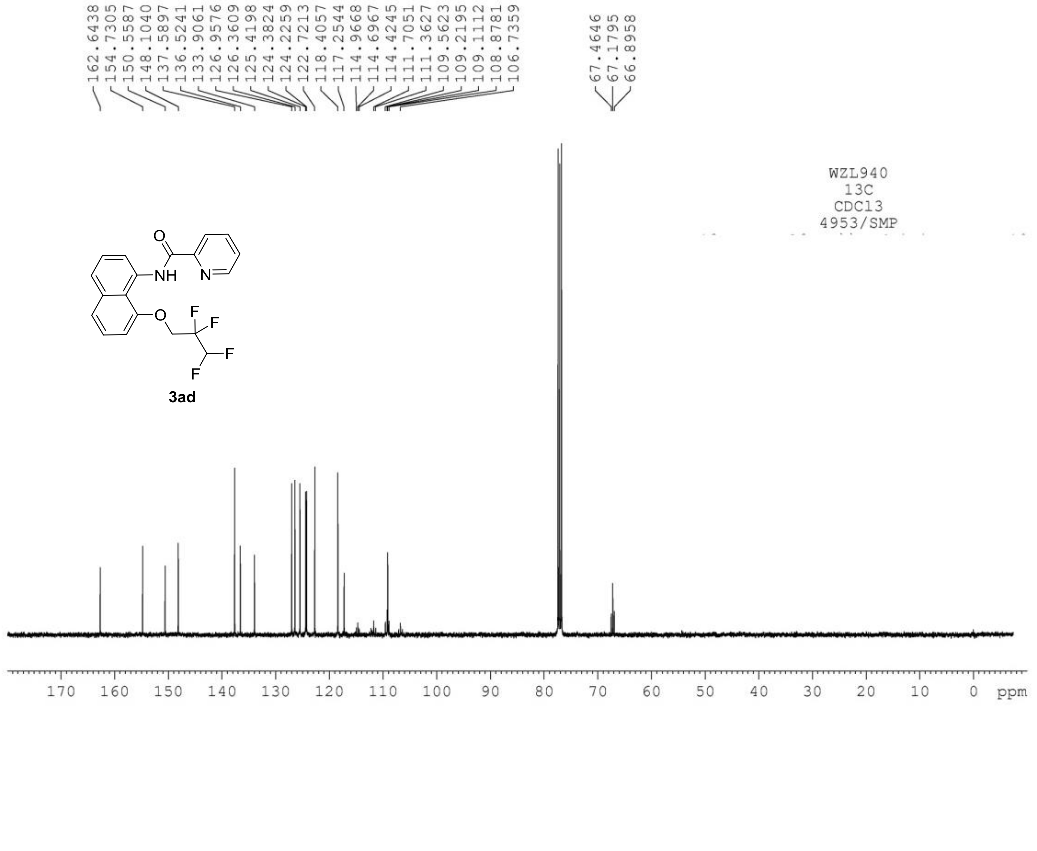
WZL940
13C
CDCl3
4953/SMP
3ad
ppm

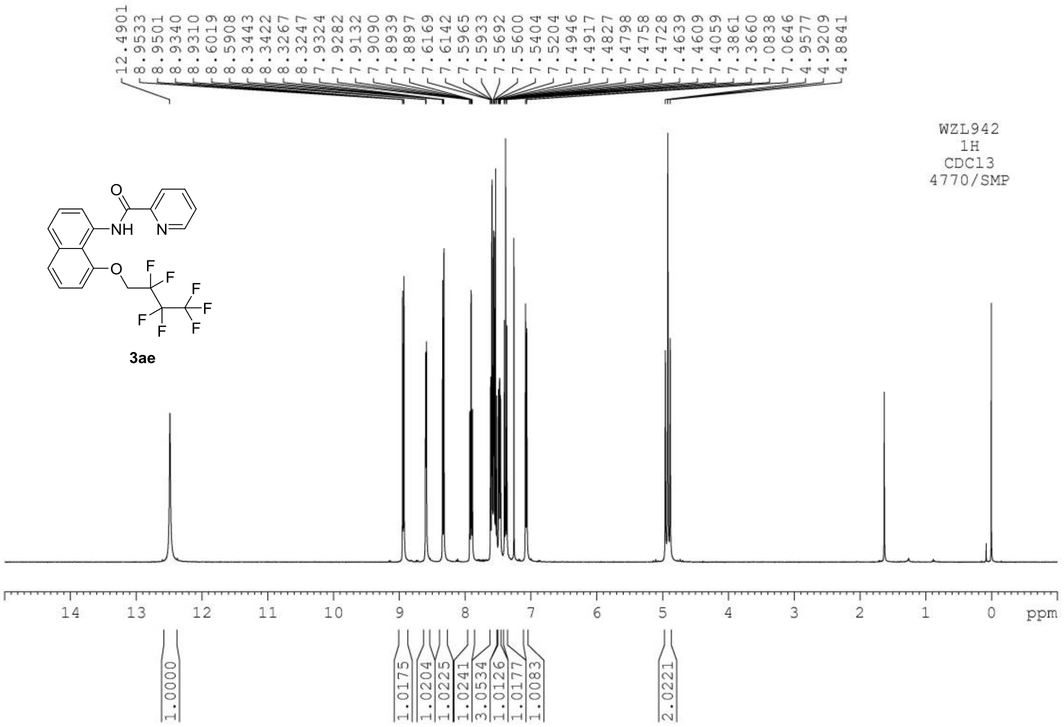
12.4901 8.9533 8.9501 8.9340 8.9310 8.6019 8.5908 8.3443 8.3422 8.3267 8.3247 7.9324 7.9282 7.9132 7.9090 7.8939 7.8897 7.6169 7.6142 7.5965 7.5933 7.5692 7.5600 7.5404 7.5204 7.4946 7.4917 7.4827 7.4798 7.4758 7.4728 7.4639 7.4609 7.4059 7.3861 7.3660 7.0838 7.0646 4.9577 4.9209 4.8841
WZL942
1H
CDCl3
4770/SMP
O
NH
N
O
F
F
F
F
F
F
F
3ae
14 13 12 11 10 9 8 7 6 5 4 3 2 1 0 ppm
1.0000 1.0175 1.0204 1.0225 1.0241 3.0534 1.0126 1.0177 1.0083 2.0221

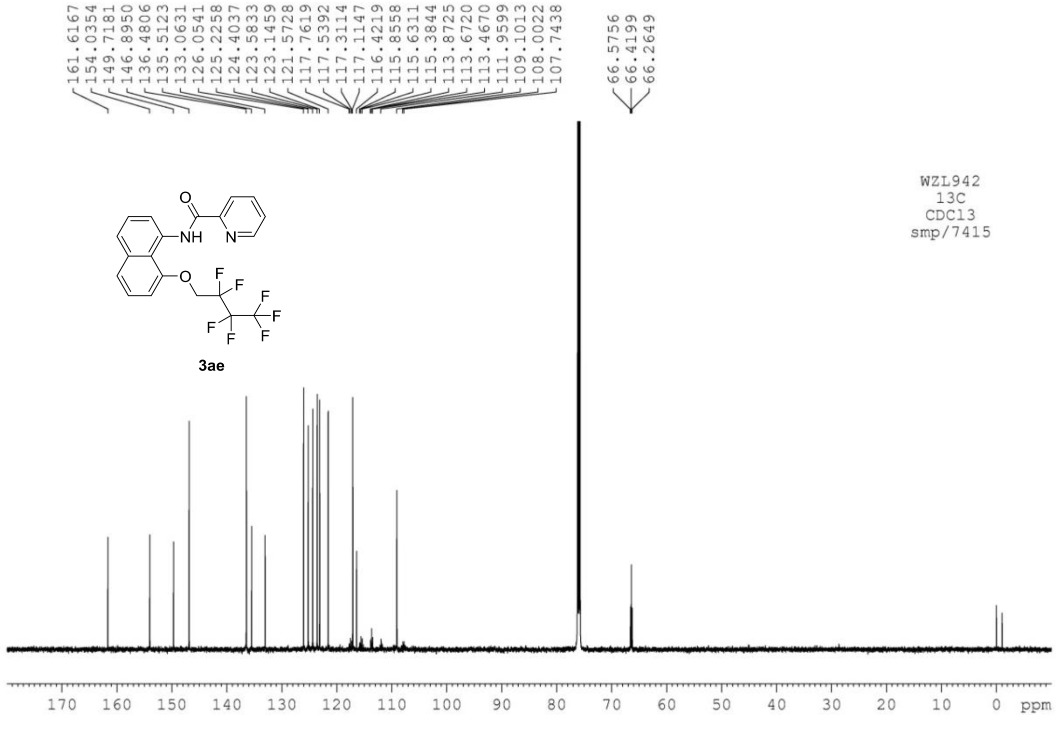
161.6167 154.0354 149.7181 146.8950 136.4806 135.5123 133.0631 126.0541 125.2258 124.4037 123.5833 123.1459 121.5728 117.7619 117.5392 117.3114 117.1147 116.4219 115.8558 115.6311 115.3844 113.8725 113.6720 113.4670 111.9599 109.1013 108.0022 107.7438
66.5756 66.4199 66.2649
WZL942
13C
CDCl3
smp/7415
O
NH
N
O
F
F
F
F
F
F
F
3ae
170 160 150 140 130 120 110 100 90 80 70 60 50 40 30 20 10 0 ppm

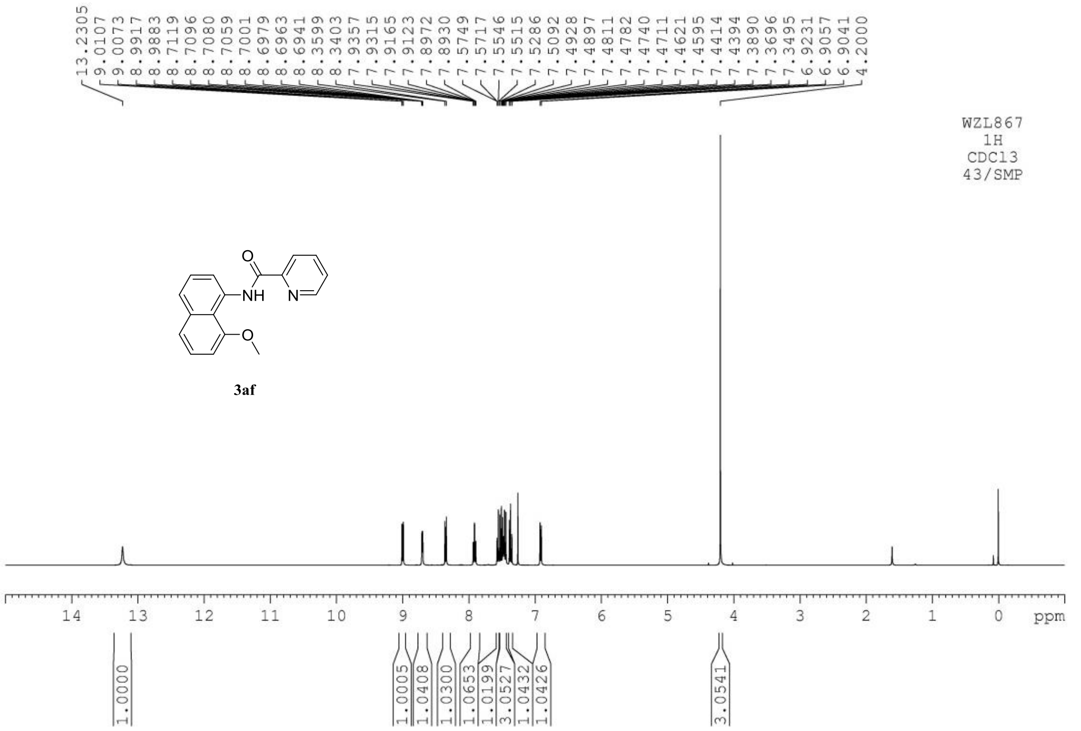
13.2305
9.0107
9.0073
8.9917
8.9883
8.7119
8.7096
8.7080
8.7059
8.7001
8.6979
8.6963
8.6941
8.3599
8.3403
7.9357
7.9315
7.9165
7.9123
7.8972
7.8930
7.5749
7.5717
7.5546
7.5515
7.5286
7.5092
7.4928
7.4897
7.4811
7.4782
7.4740
7.4711
7.4621
7.4595
7.4414
7.4394
7.3890
7.3696
7.3495
6.9231
6.9057
6.9041
4.2000
WZL867
1H
CDCl3
43/SMP
O
NH
N
O
3af
14 13 12 11 10 9 8 7 6 5 4 3 2 1 0 ppm
1.0000
1.0005
1.0408
1.0300
1.0653
1.0199
3.0527
1.0432
1.0426
3.0541

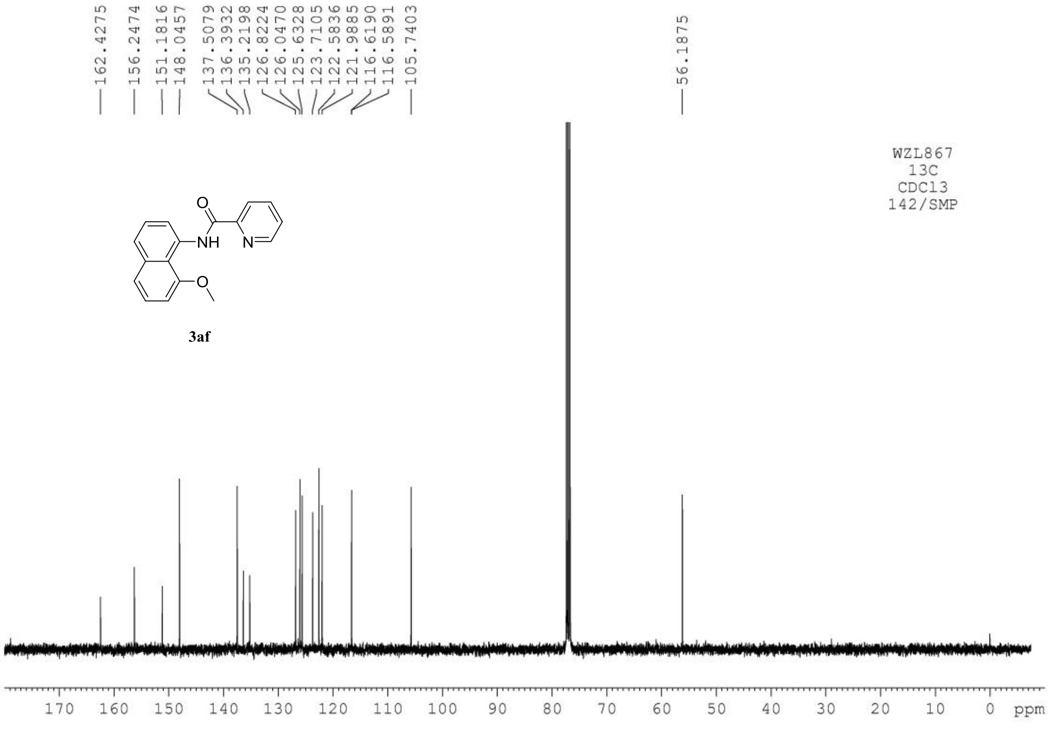
162.4275
156.2474
151.1816
148.0457
137.5079
136.3932
135.2198
126.8224
126.0470
125.6328
123.7105
122.5836
121.9885
116.6190
116.5891
105.7403
56.1875
WZL867
13C
CDCl3
142/SMP
O
NH
N
O
3af
170 160 150 140 130 120 110 100 90 80 70 60 50 40 30 20 10 0 ppm

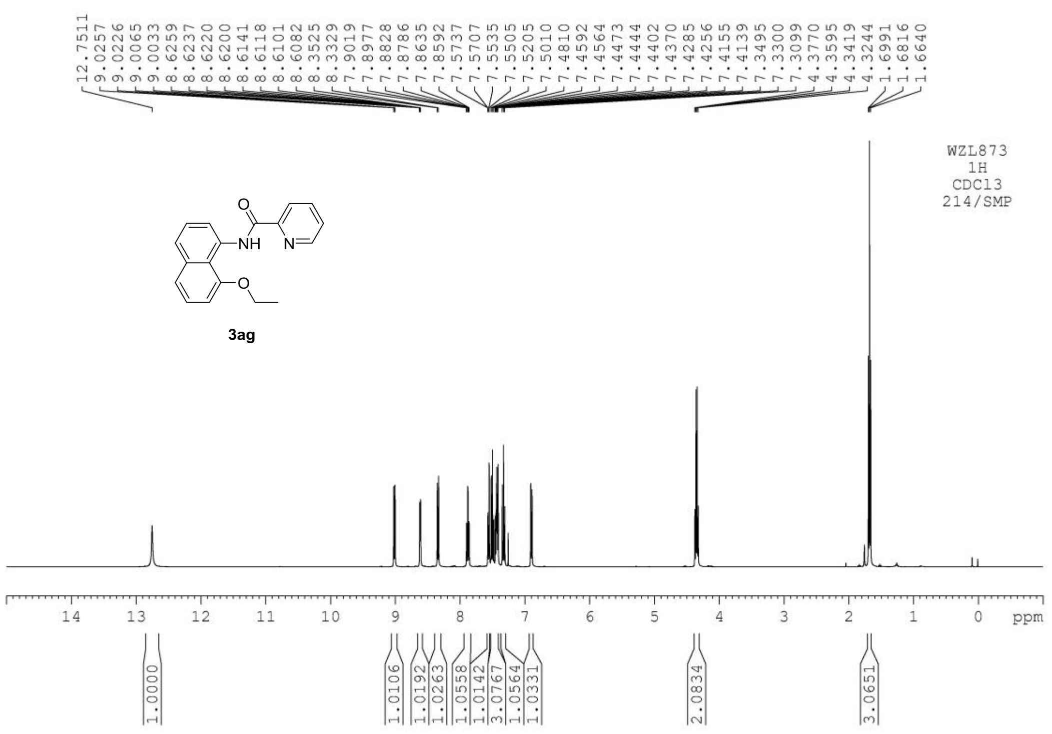
WZL873
1H
CDCl3
214/SMP
3ag
ppm

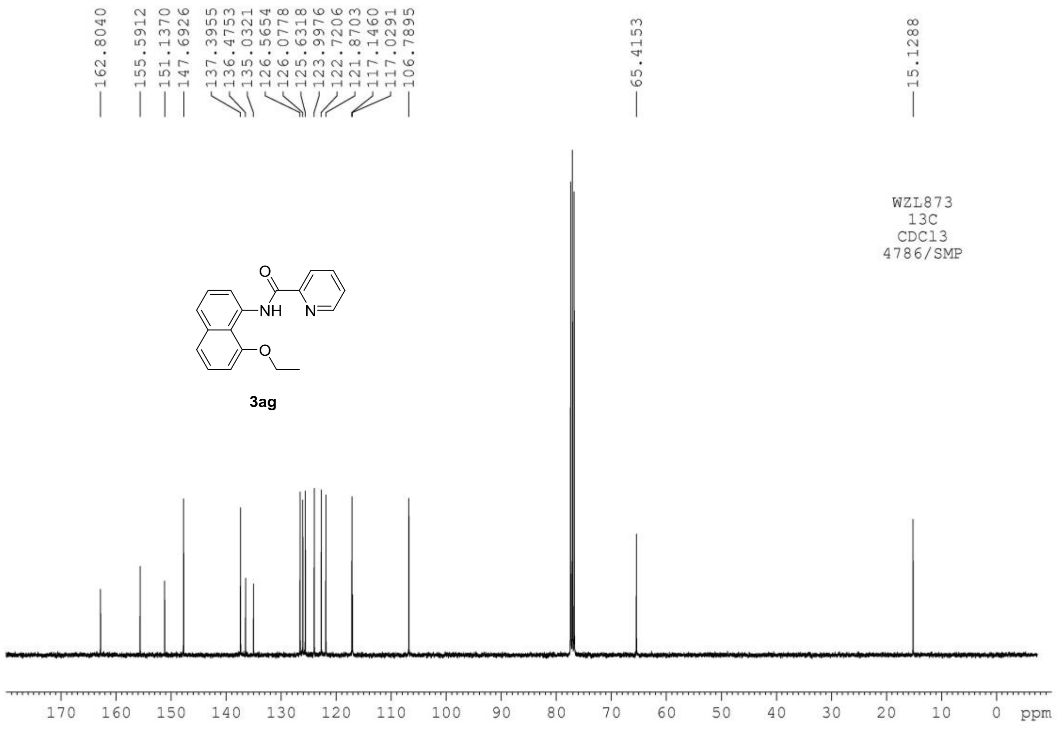
162.8040
155.5912
151.1370
147.6926
137.3955
136.4753
135.0321
126.5654
126.0778
125.6318
123.9976
122.7206
121.8703
117.1460
117.0291
106.7895
65.4153
15.1288
WZL873
13C
CDCl3
4786/SMP
3ag
ppm

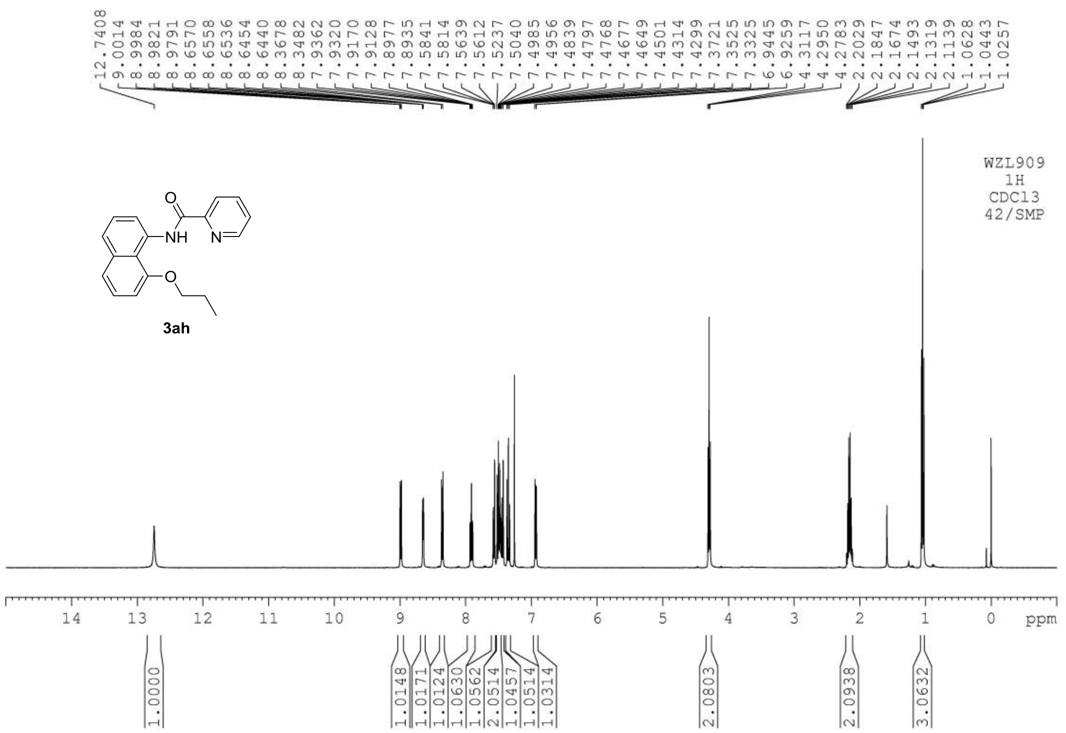
12.7408
9.0014
8.9984
8.9821
8.9791
8.6570
8.6558
8.6536
8.6454
8.6440
8.3678
8.3482
7.9362
7.9320
7.9170
7.9128
7.8977
7.8935
7.5841
7.5814
7.5639
7.5612
7.5237
7.5040
7.4985
7.4956
7.4839
7.4797
7.4768
7.4677
7.4649
7.4501
7.4314
7.4299
7.3721
7.3525
7.3325
6.9445
6.9259
4.3117
4.2950
4.2783
2.2029
2.1847
2.1674
2.1493
2.1319
2.1139
1.0628
1.0443
1.0257
WZL909
1H
CDCl3
42/SMP
O
NH
N
O
3ah
14
13
12
11
10
9
8
7
6
5
4
3
2
1
0
ppm
1.0000
1.0148
1.0171
1.0124
1.0630
1.0562
2.0514
1.0457
1.0514
1.0314
2.0803
2.0938
3.0632

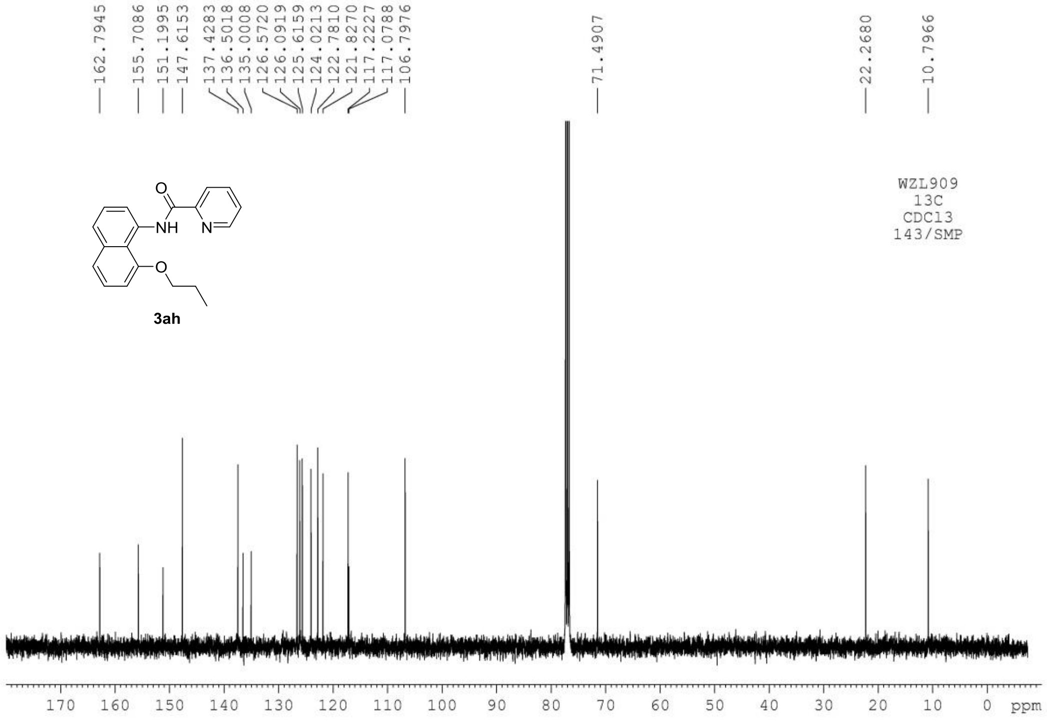
162.7945
155.7086
151.1995
147.6153
137.4283
136.5018
135.0008
126.5720
126.0919
125.6159
124.0213
122.7810
121.8270
117.2227
117.0788
106.7976
71.4907
22.2680
10.7966
WZL909
13C
CDCl3
143/SMP
O
NH
N
O
3ah
170
160
150
140
130
120
110
100
90
80
70
60
50
40
30
20
10
0
ppm

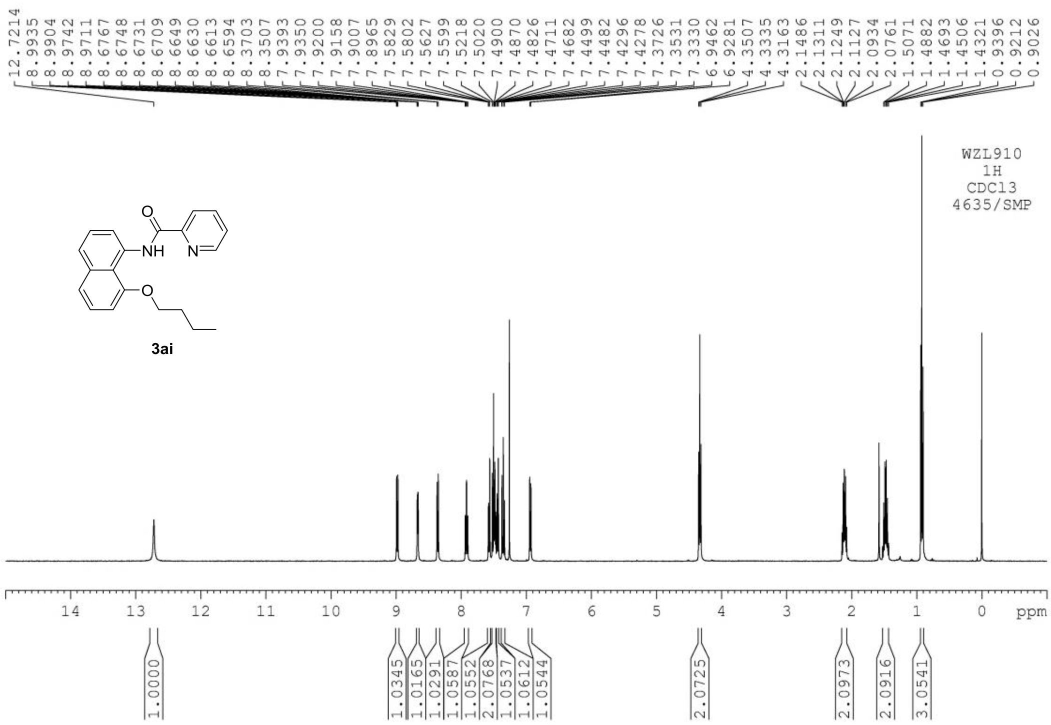
12.7214
8.9935
8.9904
8.9742
8.9711
8.6767
8.6748
8.6731
8.6709
8.6649
8.6630
8.6613
8.6594
8.3703
8.3507
7.9350
7.9393
7.9200
7.9158
7.9007
7.8965
7.5829
7.5802
7.5627
7.5599
7.5218
7.5020
7.4900
7.4870
7.4826
7.4711
7.4682
7.4499
7.4482
7.4296
7.4278
7.3726
7.3531
7.3330
6.9462
6.9281
4.3507
4.3335
4.3163
2.1486
2.1311
2.1249
2.1127
2.0934
2.0761
1.5071
1.4882
1.4693
1.4506
1.4321
0.9396
0.9212
0.9026
WZL910
1H
CDCl3
4635/SMP
O
NH
N
O
3ai
14 13 12 11 10 9 8 7 6 5 4 3 2 1 0 ppm
1.0000
1.0345
1.0165
1.0291
1.0587
1.0552
2.0768
1.0537
1.0612
1.0544
2.0725
2.0973
2.0916
3.0541

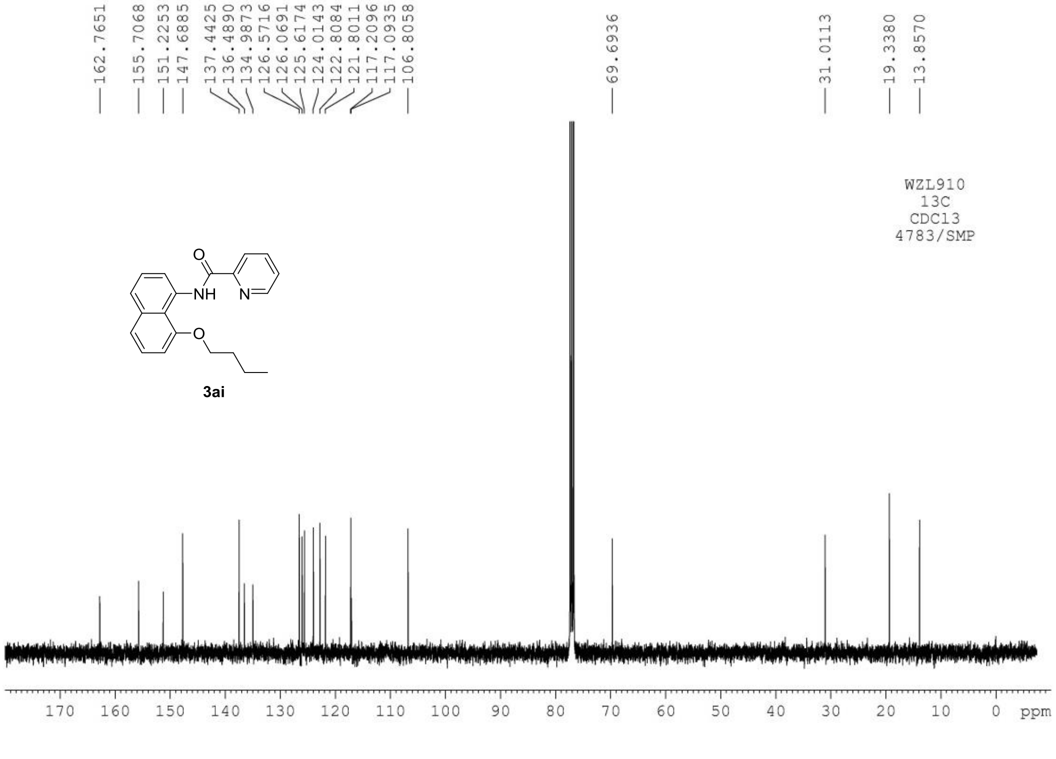
162.7651
155.7068
151.2253
147.6885
137.4425
136.4890
134.9873
126.5716
126.0691
125.6174
124.0143
122.8084
121.8011
117.2096
117.0935
106.8058
69.6936
31.0113
19.3380
13.8570
WZL910
13C
CDCl3
4783/SMP
O
NH
N
O
3ai
170 160 150 140 130 120 110 100 90 80 70 60 50 40 30 20 10 0 ppm

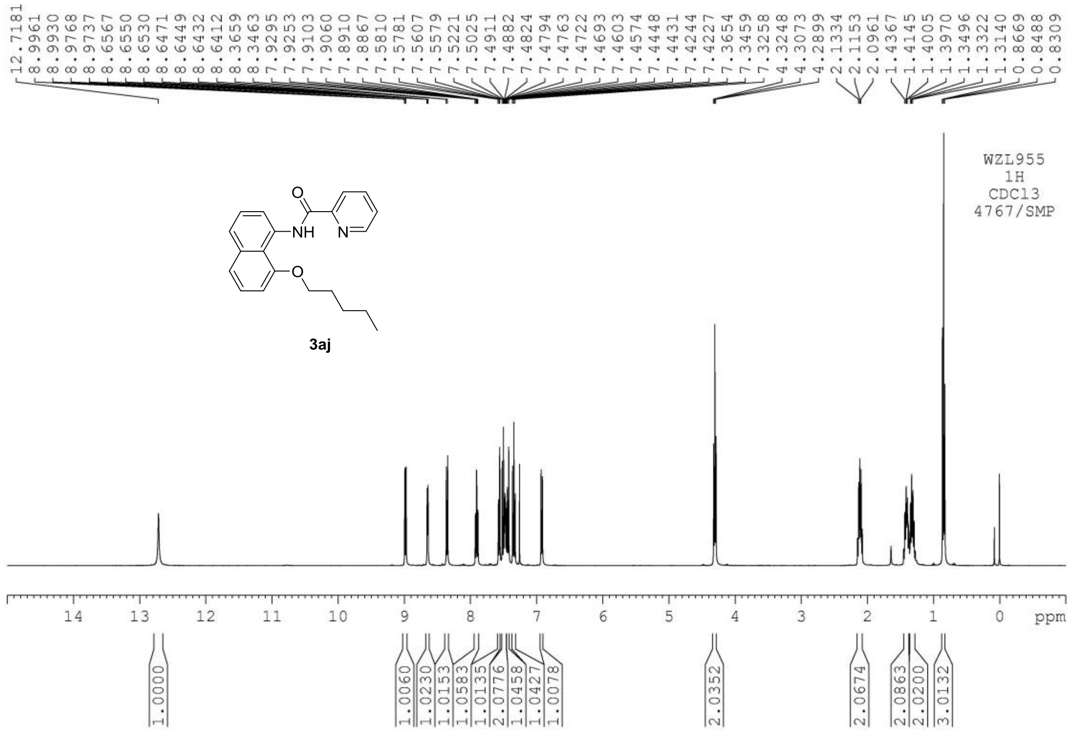
12.7181
8.9961
8.9930
8.9768
8.9737
8.6567
8.6550
8.6530
8.6471
8.6449
8.6432
8.6412
8.3659
8.3463
7.9295
7.9253
7.9103
7.9060
7.8910
7.8867
7.5810
7.5781
7.5607
7.5579
7.5221
7.5025
7.4911
7.4882
7.4824
7.4794
7.4763
7.4722
7.4693
7.4603
7.4574
7.4448
7.4431
7.4244
7.4227
7.3654
7.3459
7.3258
4.3248
4.3073
4.2899
2.1334
2.1153
2.0961
1.4367
1.4145
1.4005
1.3970
1.3496
1.3322
1.3140
0.8669
0.8488
0.8309
WZL955
1H
CDCl3
4767/SMP
O
NH
N
O
3aj
ppm
1.0000
1.0060
1.0230
1.0153
1.0583
1.0135
2.0776
1.0458
1.0427
1.0078
2.0352
2.0674
2.0863
2.0200
3.0132

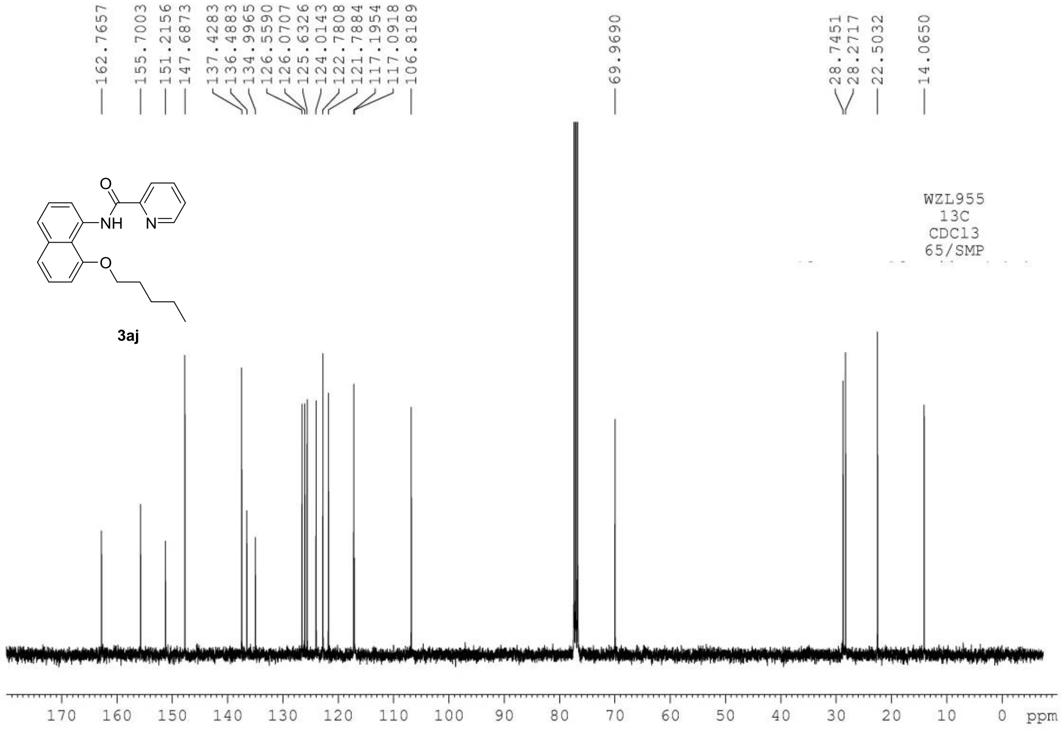
162.7657
155.7003
151.2156
147.6873
137.4283
136.4883
134.9965
126.5590
126.0707
125.6326
124.0143
122.7808
121.7884
117.1954
117.0918
106.8189
69.9690
28.7451
28.2717
22.5032
14.0650
WZL955
13C
CDCl3
65/SMP
O
NH
N
O
3aj
ppm

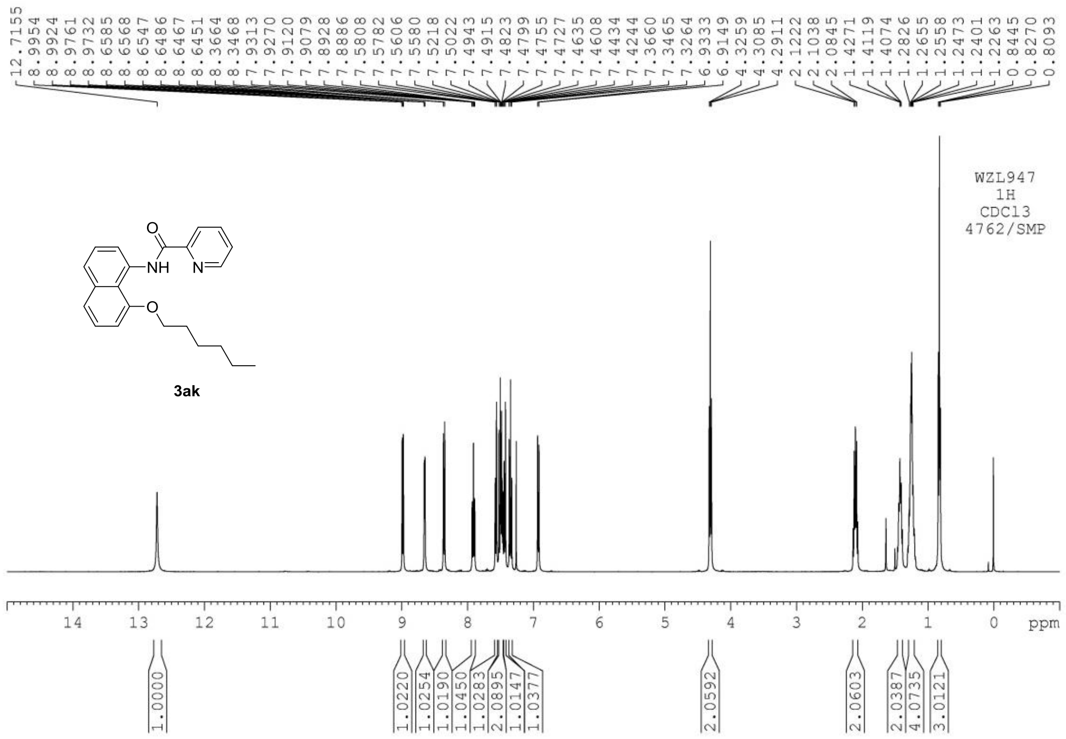
WZL947
1H
CDCl3
4762/SMP
3ak
ppm

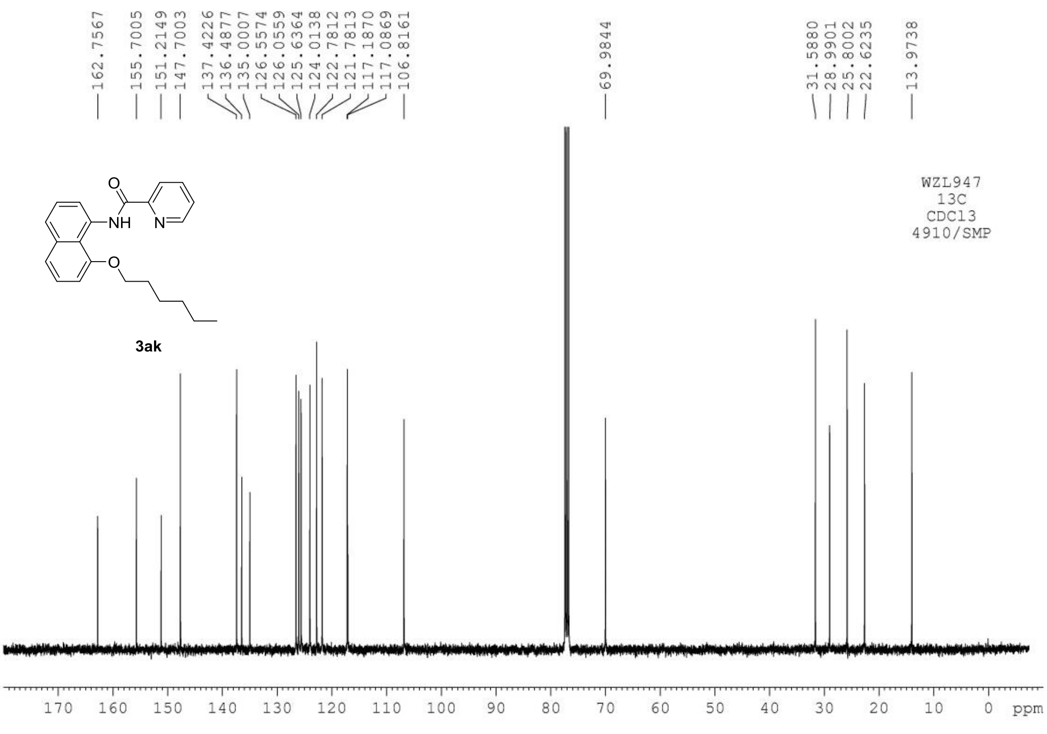
WZL947
13C
CDCl3
4910/SMP
3ak
ppm

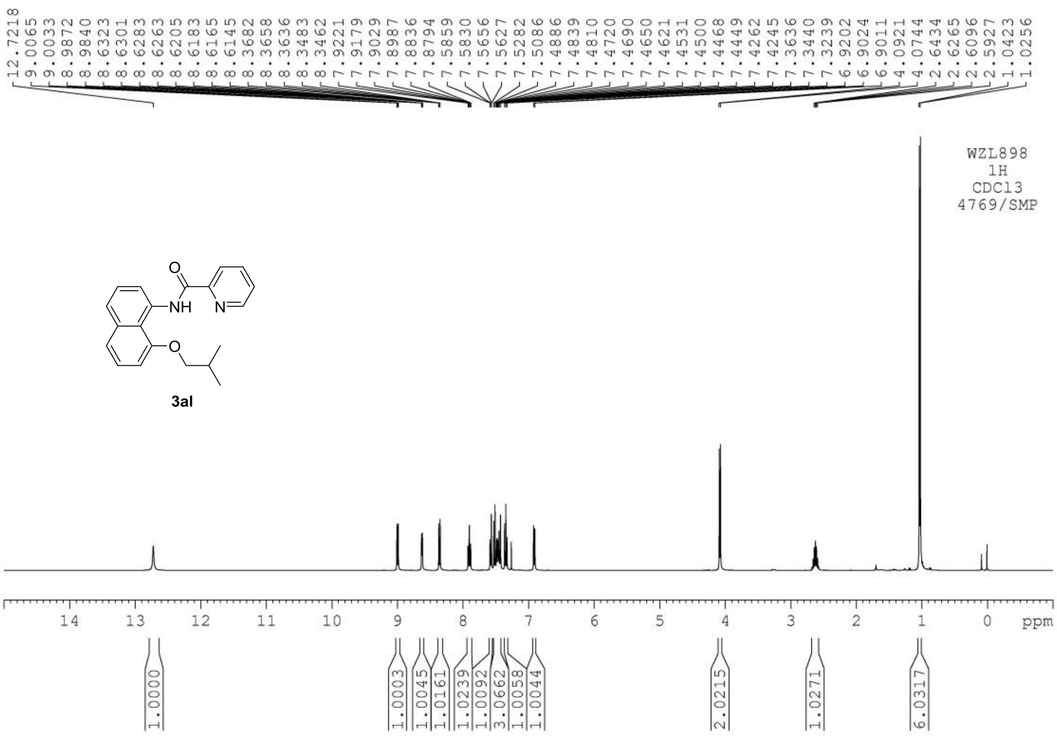
12.7218
9.0065
9.0033
8.9872
8.9840
8.6323
8.6301
8.6283
8.6263
8.6205
8.6183
8.6165
8.6145
8.3682
8.3658
8.3636
8.3483
8.3462
7.9221
7.9179
7.9029
7.8987
7.8836
7.8794
7.5859
7.5830
7.5656
7.5627
7.5282
7.5086
7.4886
7.4839
7.4810
7.4720
7.4690
7.4650
7.4621
7.4531
7.4500
7.4468
7.4449
7.4262
7.4245
7.3636
7.3440
7.3239
6.9202
6.9024
6.9011
4.0921
4.0744
2.6434
2.6265
2.6096
2.5927
1.0423
1.0256
WZL898
1H
CDCl3
4769/SMP
3al
14 13 12 11 10 9 8 7 6 5 4 3 2 1 0 ppm
1.0000
1.0003
1.0045
1.0161
1.0239
1.0092
3.0662
1.0058
1.0044
2.0215
1.0271
6.0317

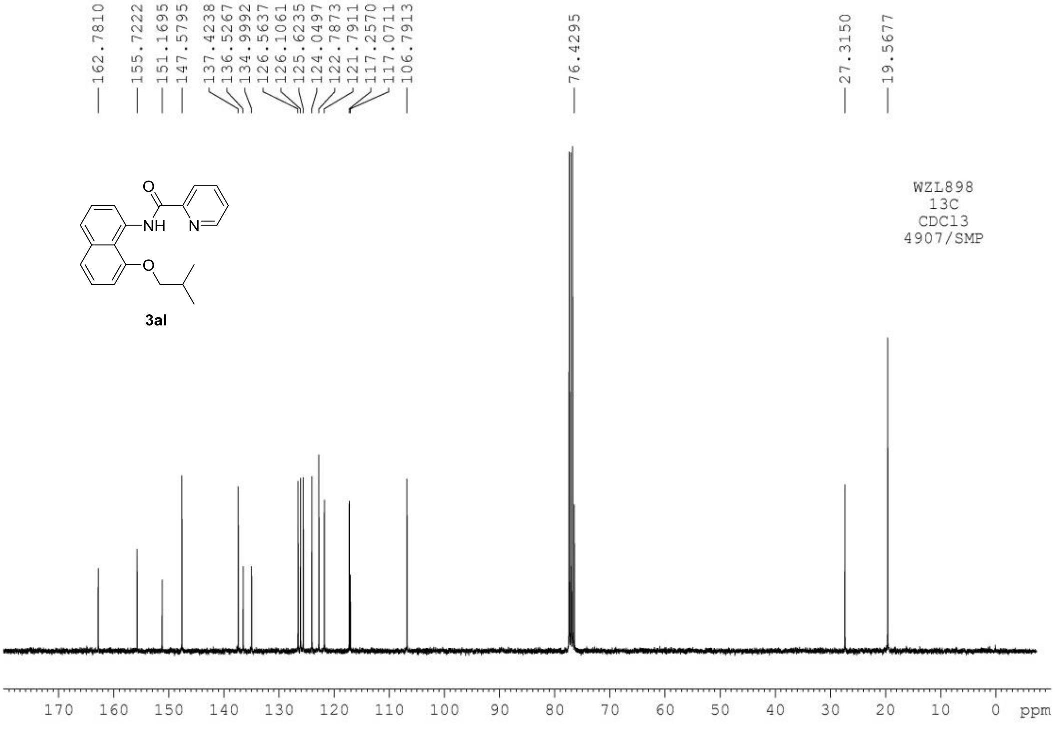
162.7810
155.7222
151.1695
147.5795
137.4238
136.5267
134.9992
126.5637
126.1061
125.6235
124.0497
122.7873
121.7911
117.2570
117.0711
106.7913
76.4295
27.3150
19.5677
WZL898
13C
CDCl3
4907/SMP
3al
170 160 150 140 130 120 110 100 90 80 70 60 50 40 30 20 10 0 ppm

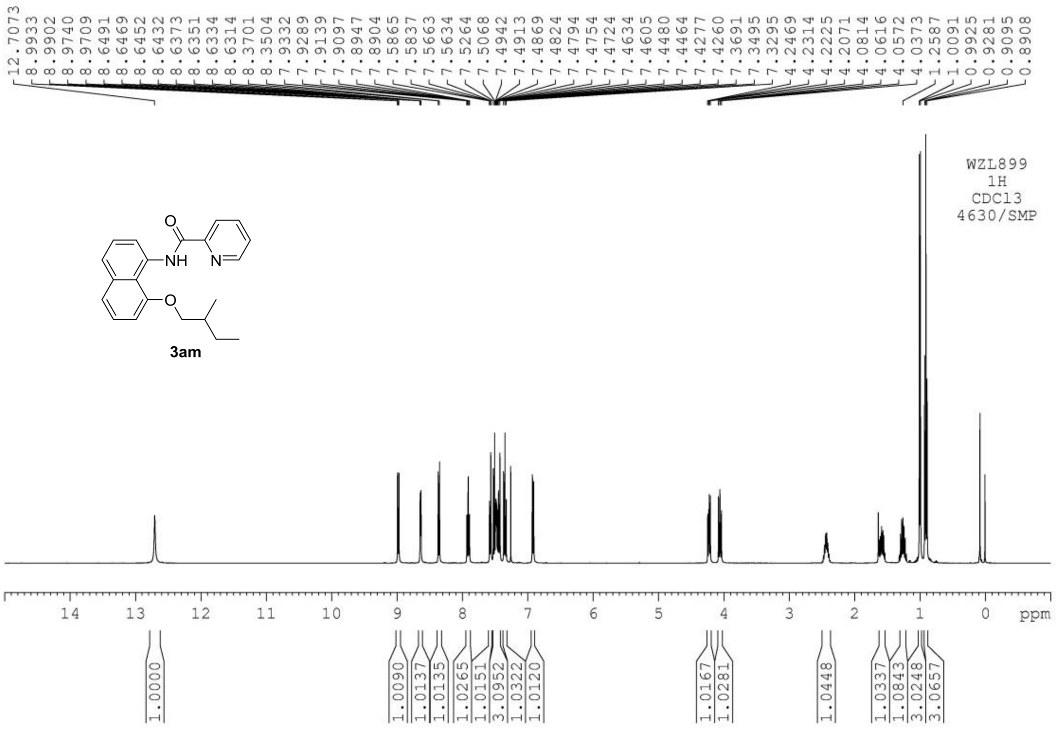
WZL899
1H
CDCl3
4630/SMP
3am
ppm

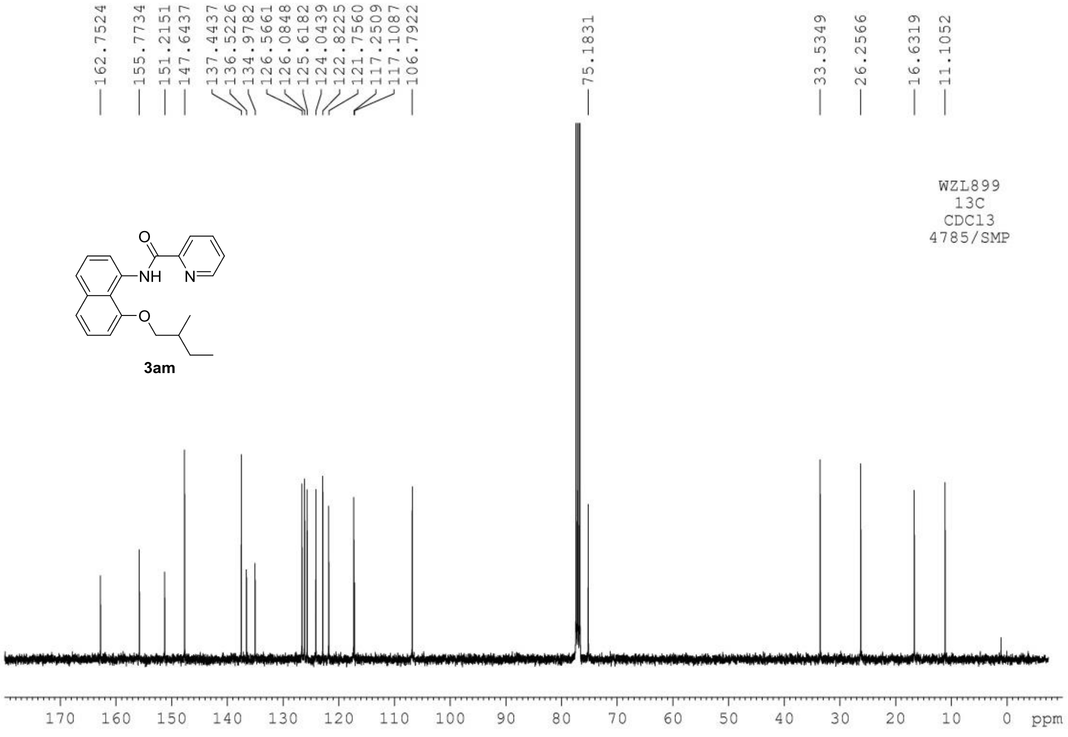
WZL899
13C
CDCl3
4785/SMP
3am
ppm

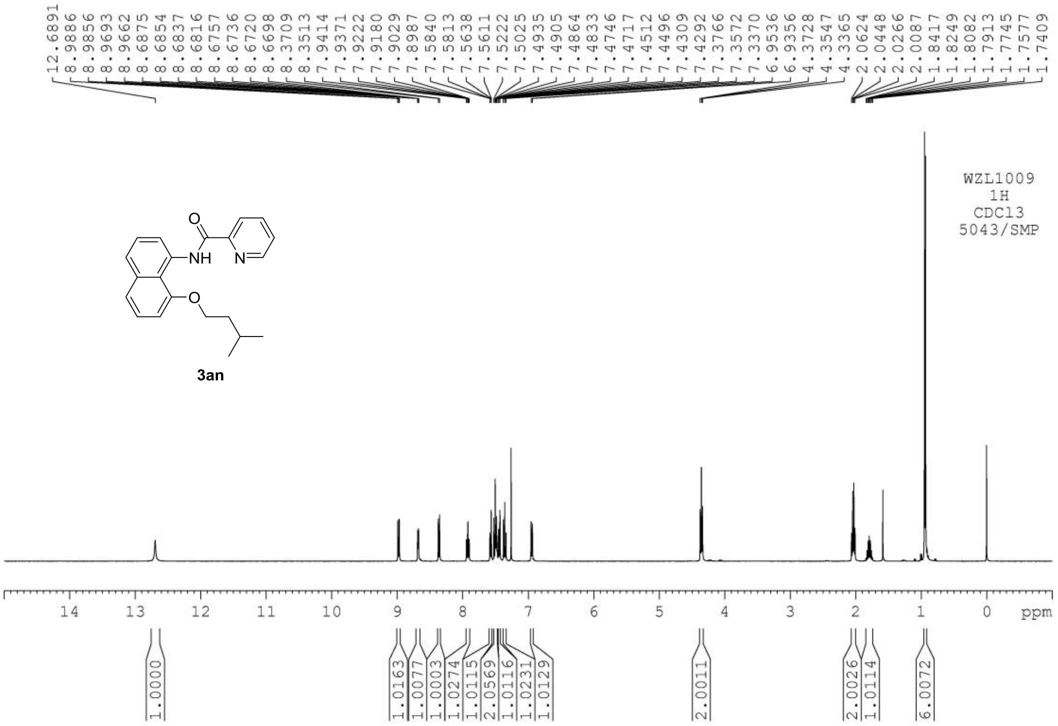
WZL1009
1H
CDCl3
5043/SMP
3an
ppm

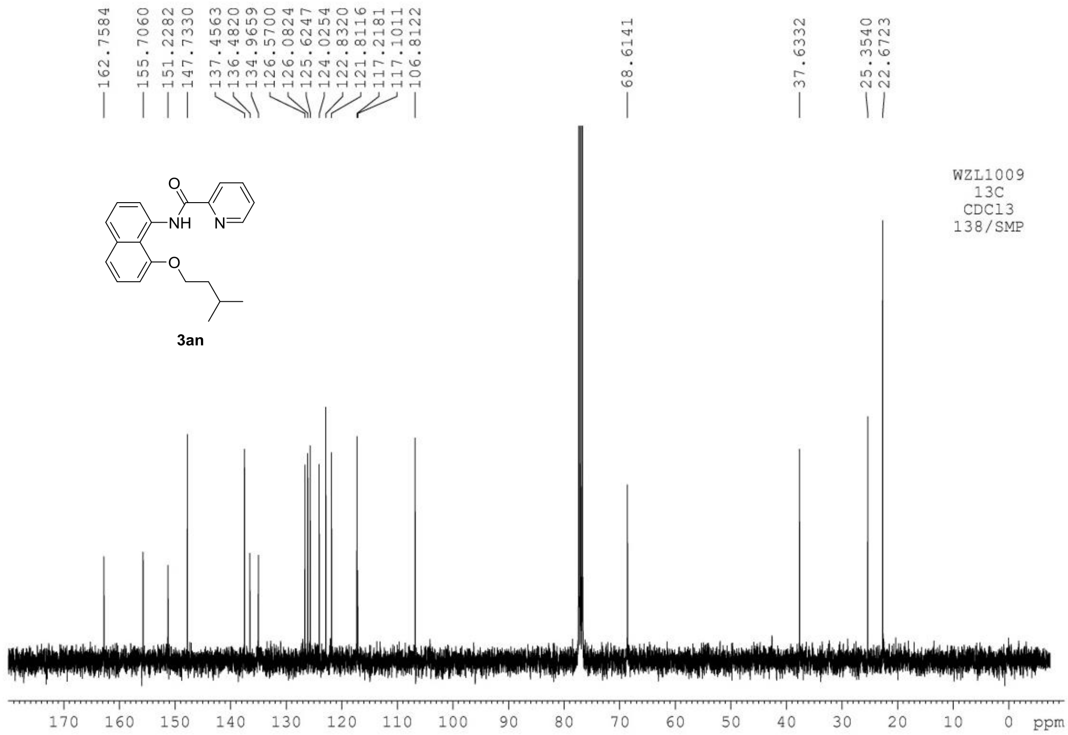
WZL1009
13C
CDCl3
138/SMP
3an
ppm

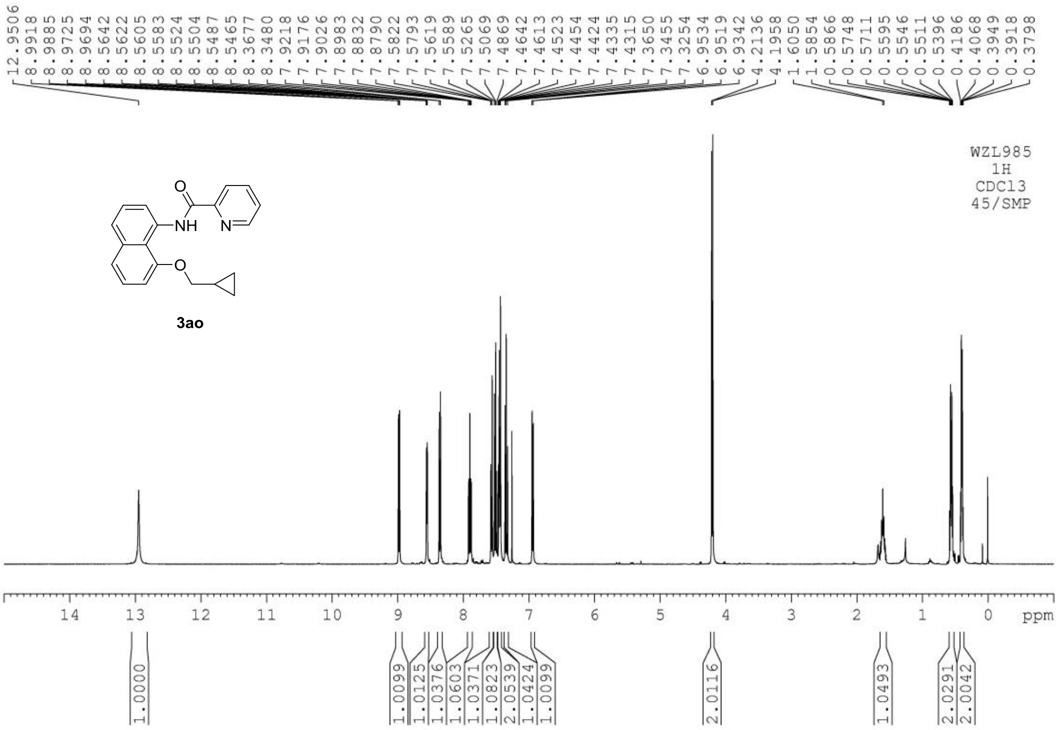
WZL985
1H
CDCl3
45/SMP
3ao
ppm

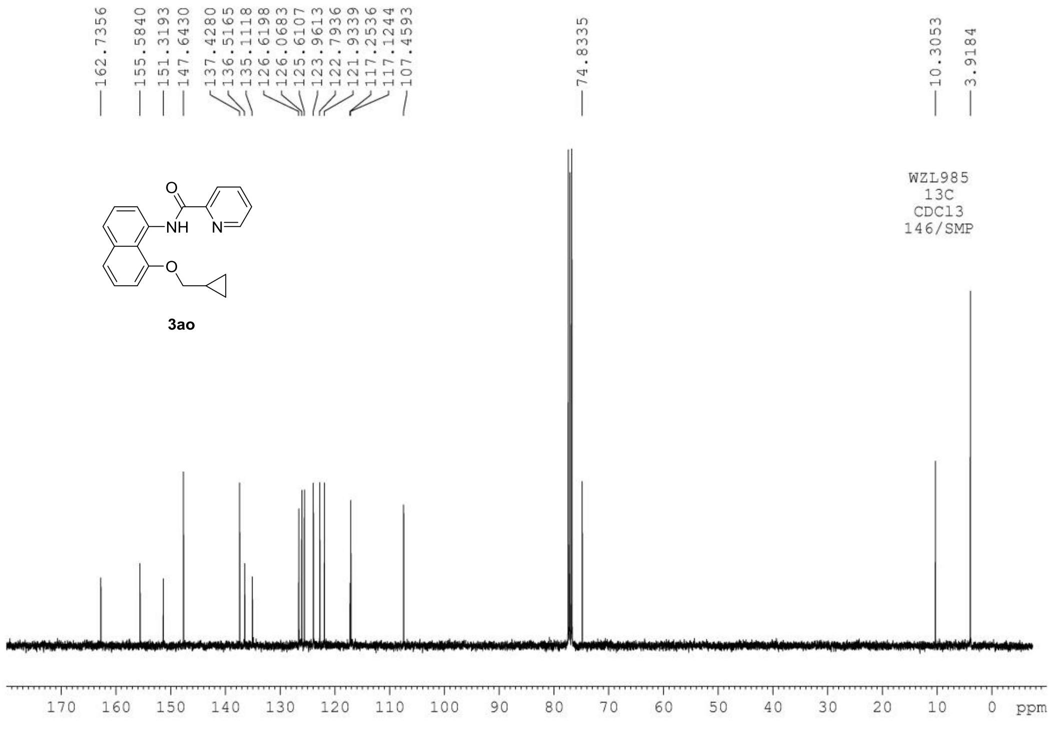
WZL985
13C
CDCl3
146/SMP
3ao
ppm

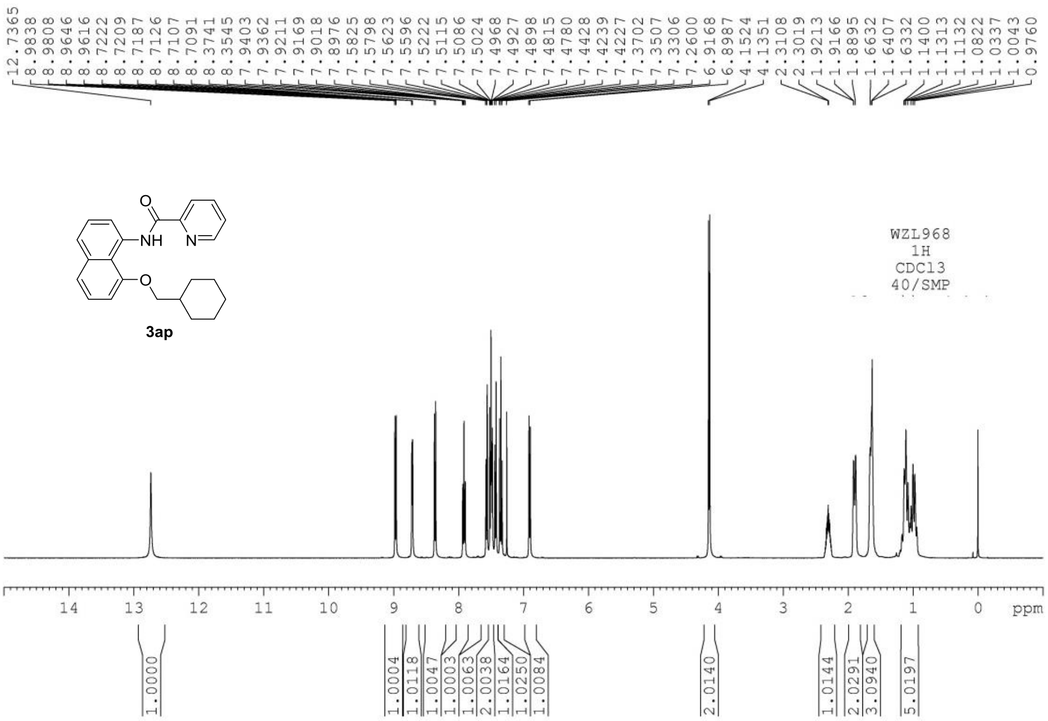
12.7365
8.9838
8.9808
8.9646
8.9616
8.7222
8.7209
8.7187
8.7126
8.7107
8.7091
8.3741
8.3545
7.9403
7.9362
7.9211
7.9169
7.9018
7.8976
7.5825
7.5798
7.5623
7.5596
7.5222
7.5115
7.5086
7.5024
7.4968
7.4927
7.4898
7.4815
7.4780
7.4428
7.4239
7.4227
7.3702
7.3507
7.3306
7.2600
6.9168
6.8987
4.1524
4.1351
2.3108
2.3019
1.9213
1.9166
1.8895
1.6632
1.6407
1.6332
1.1400
1.1313
1.1132
1.0822
1.0337
1.0043
0.9760
O
NH
N
O
3ap
WZL968
1H
CDCl3
40/SMP
14
13
12
11
10
9
8
7
6
5
4
3
2
1
0
ppm
1.0000
1.0004
1.0118
1.0047
1.0003
1.0063
2.0038
1.0164
1.0250
1.0084
2.0140
1.0144
2.0291
3.0940
5.0197

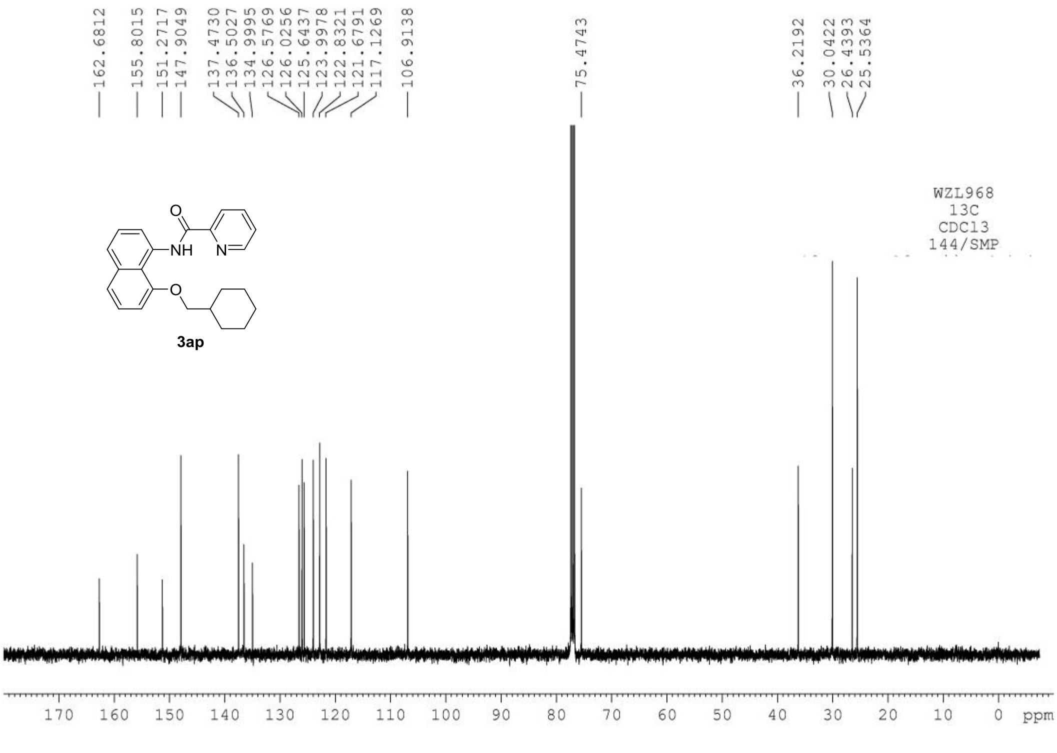
162.6812
155.8015
151.2717
147.9049
137.4730
136.5027
134.9995
126.5769
126.0256
125.6437
123.9978
122.8321
121.6791
117.1269
106.9138
75.4743
36.2192
30.0422
26.4393
25.5364
O
NH
N
O
3ap
WZL968
13C
CDCl3
144/SMP
170
160
150
140
130
120
110
100
90
80
70
60
50
40
30
20
10
0
ppm

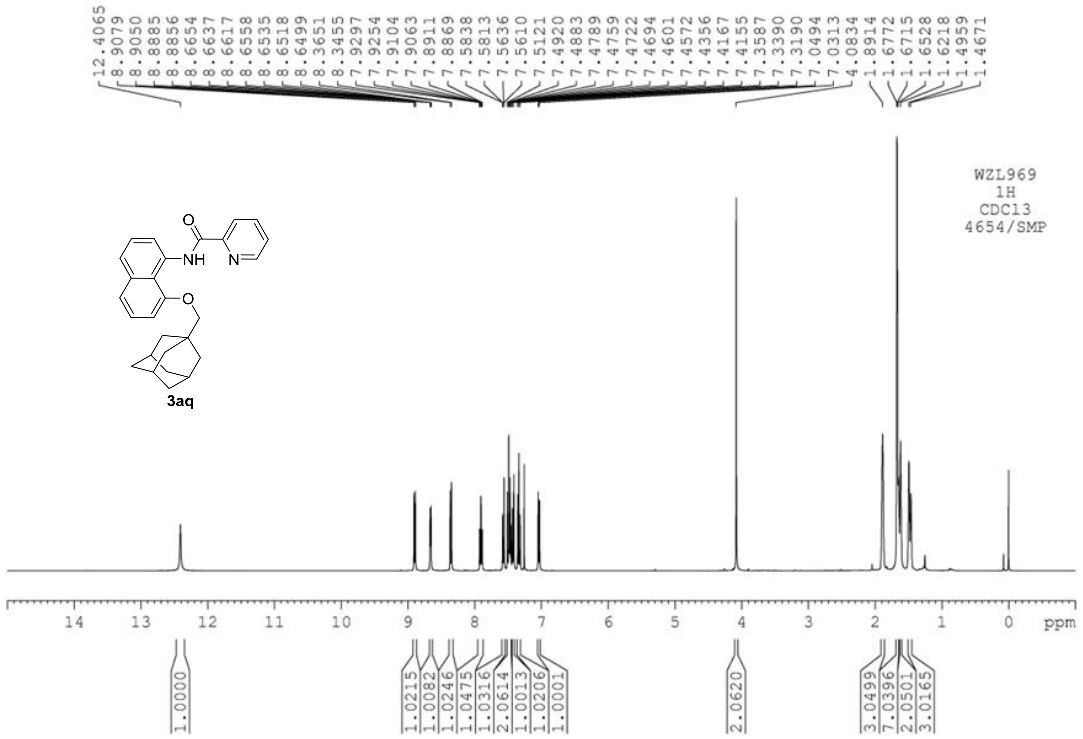
12.4065
8.9079
8.9050
8.8885
8.8856
8.6654
8.6637
8.6617
8.6558
8.6535
8.6518
8.6499
8.3651
8.3455
7.9297
7.9254
7.9104
7.9063
7.8911
7.8869
7.5838
7.5813
7.5636
7.5610
7.5121
7.4920
7.4883
7.4789
7.4759
7.4722
7.4694
7.4601
7.4572
7.4356
7.4167
7.4155
7.3587
7.3390
7.3190
7.0494
7.0313
4.0834
1.8914
1.6772
1.6715
1.6528
1.6218
1.4959
1.4671
O
NH
N
O
3aq
WZL969
1H
CDCl3
4654/SMP
ppm
1.0000
1.0215
1.0082
1.0246
1.0475
1.0316
2.0614
1.0013
1.0206
1.0001
2.0620
3.0499
7.0396
2.0501
3.0165

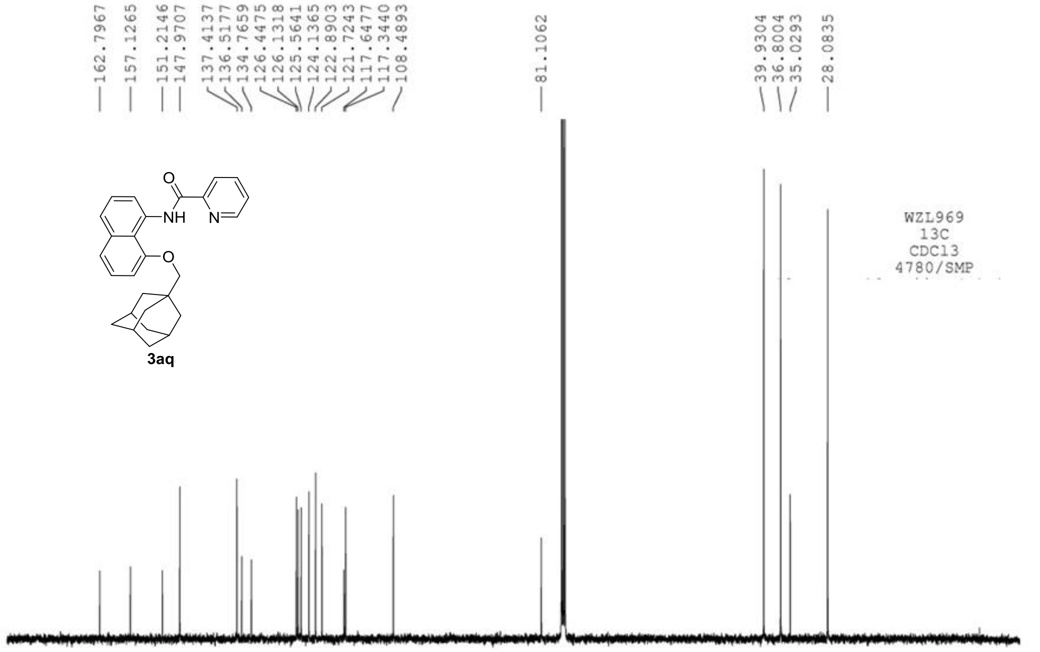
162.7967
157.1265
151.2146
147.9707
137.4137
136.5177
134.7659
126.4475
126.1318
125.5641
124.1365
122.8903
121.7243
117.6477
117.3440
108.4893
81.1062
39.9304
36.8004
35.0293
28.0835
O
NH
N
O
3aq
WZL969
13C
CDCl3
4780/SMP
ppm

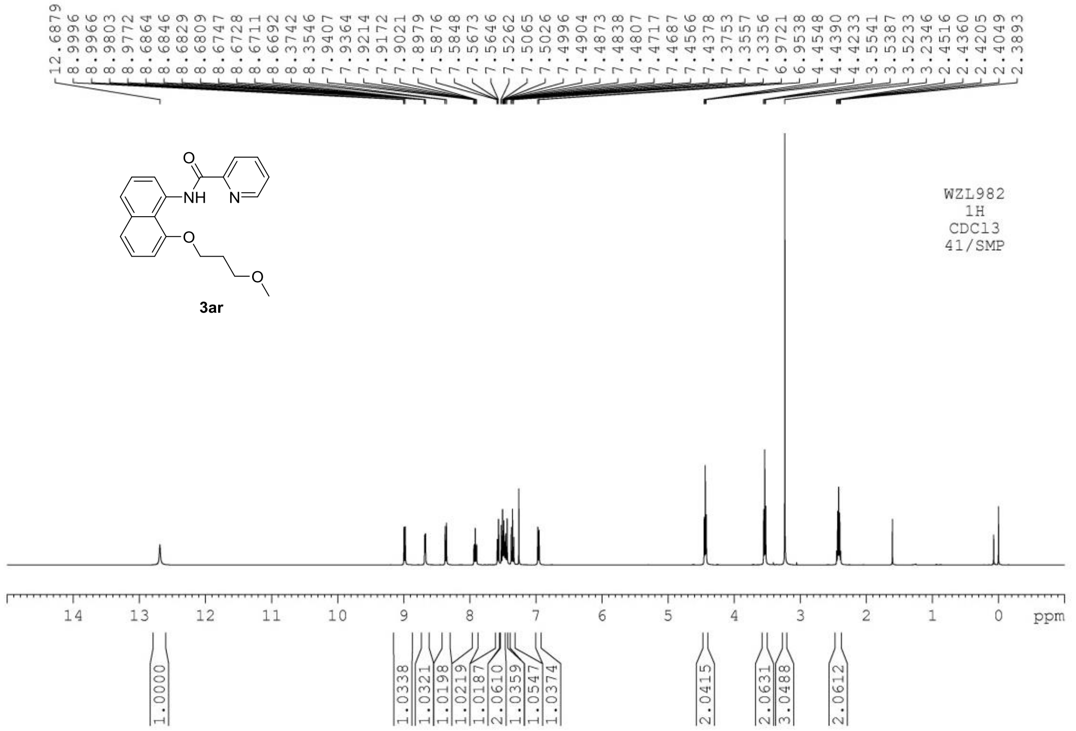

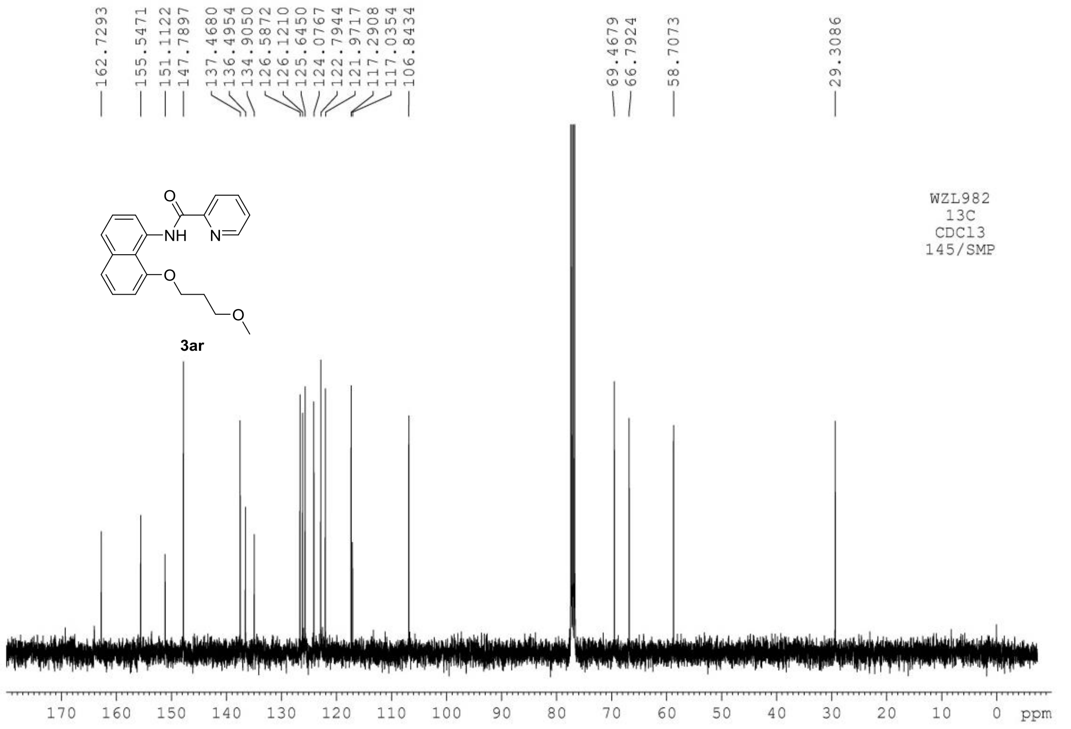

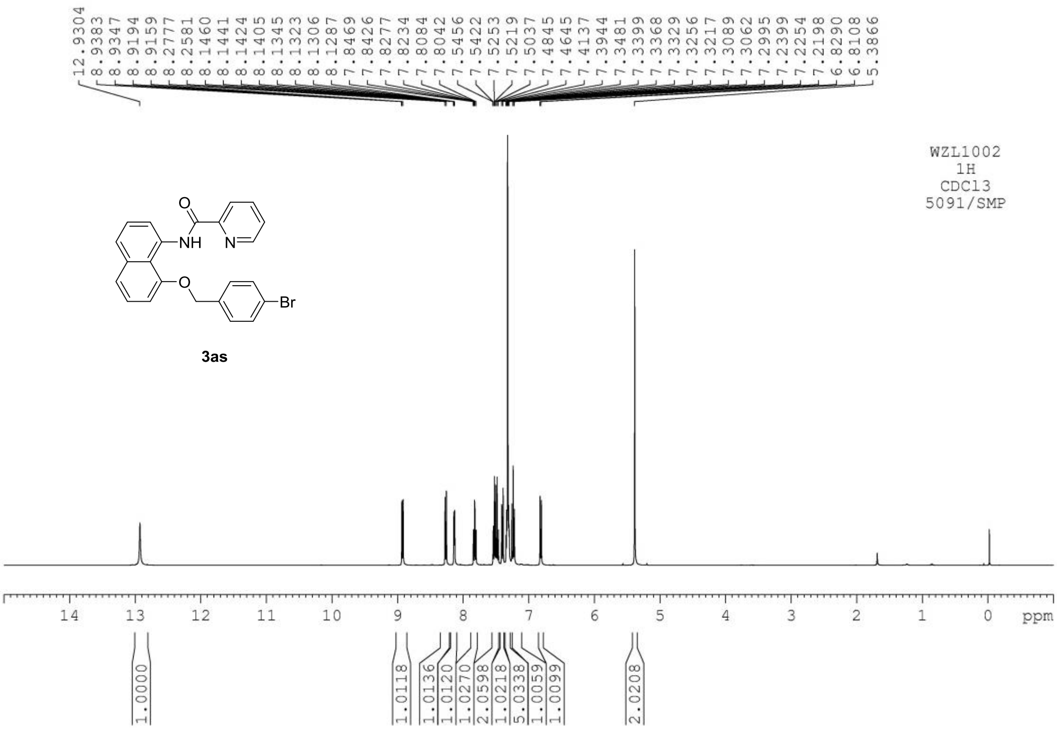
12.9304
8.9383
8.9347
8.9194
8.9159
8.2777
8.2581
8.1460
8.1441
8.1424
8.1405
8.1345
8.1323
8.1306
8.1287
7.8469
7.8426
7.8277
7.8234
7.8084
7.8042
7.5456
7.5422
7.5253
7.5219
7.5037
7.4845
7.4645
7.4137
7.3944
7.3481
7.3399
7.3368
7.3329
7.3256
7.3217
7.3089
7.3062
7.2995
7.2399
7.2254
7.2198
6.8290
6.8108
5.3866
WZL1002
1H
CDCl3
5091/SMP
O
NH
N
O
Br
3as
14 13 12 11 10 9 8 7 6 5 4 3 2 1 0 ppm
1.0000
1.0118
1.0136
1.0120
1.0270
2.0598
1.0218
5.0338
1.0059
1.0099
2.0208

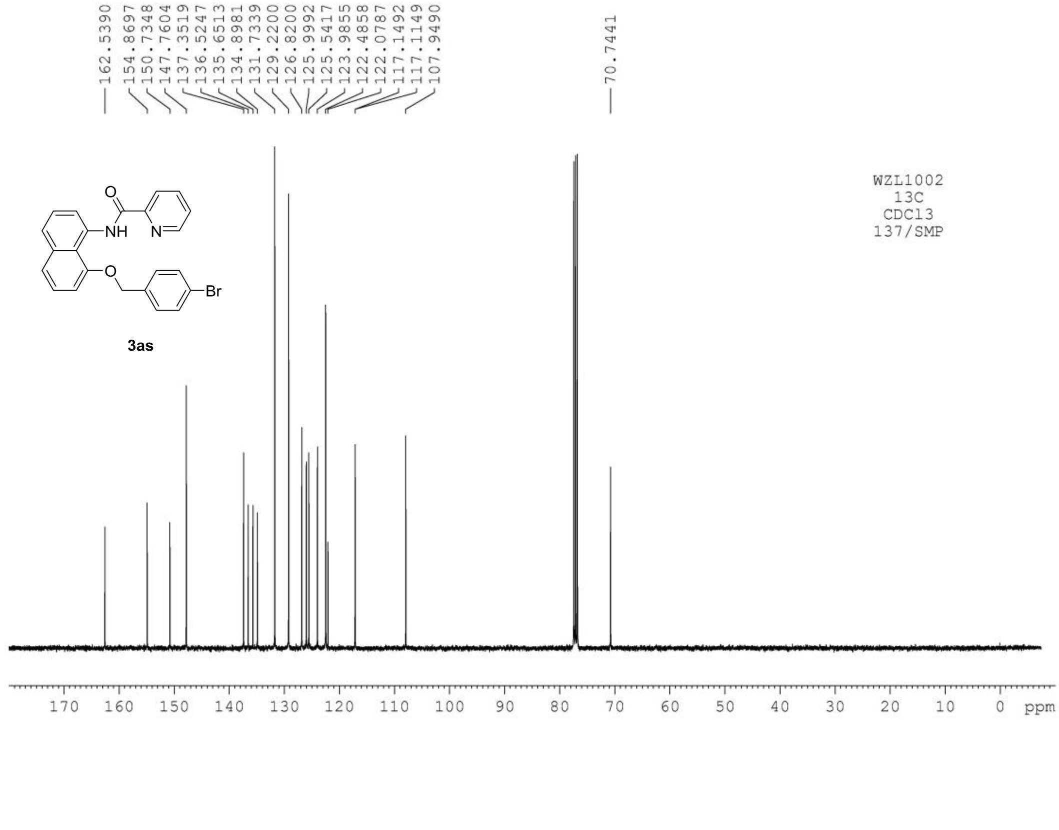
162.5390
154.8697
150.7348
147.7604
137.3519
136.5247
135.6513
134.8981
131.7339
129.2200
126.8200
125.9992
125.5417
123.9855
122.4858
122.0787
117.1492
117.1149
107.9490
70.7441
WZL1002
13C
CDCl3
137/SMP
O
NH
N
O
Br
3as
170 160 150 140 130 120 110 100 90 80 70 60 50 40 30 20 10 0 ppm

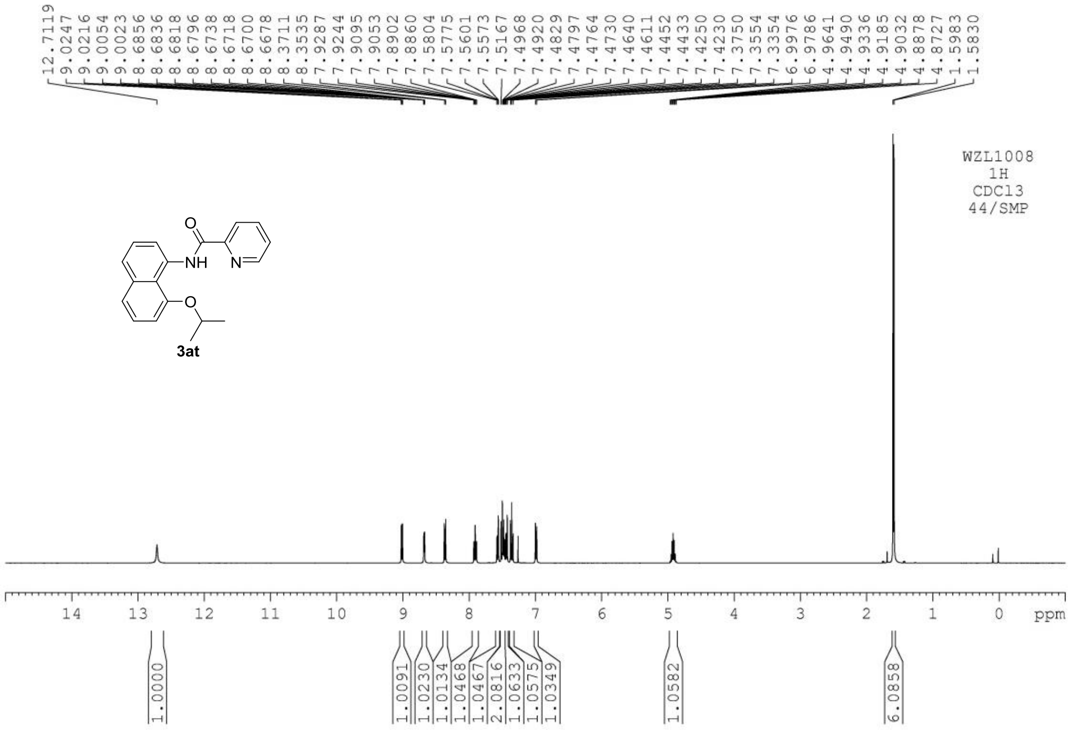
12.7119
9.0247
9.0216
9.0054
9.0023
8.6856
8.6836
8.6818
8.6796
8.6738
8.6718
8.6700
8.6678
8.3711
8.3535
7.9287
7.9244
7.9095
7.9053
7.8902
7.8860
7.5804
7.5775
7.5601
7.5573
7.5167
7.4968
7.4920
7.4829
7.4797
7.4764
7.4730
7.4640
7.4611
7.4452
7.4433
7.4250
7.4230
7.3750
7.3554
7.3354
6.9976
6.9786
4.9641
4.9490
4.9336
4.9185
4.9032
4.8878
4.8727
1.5983
1.5830
WZL1008
1H
CDCl3
44/SMP
O
NH
N
O
3at
14 13 12 11 10 9 8 7 6 5 4 3 2 1 0 ppm
1.0000
1.0091
1.0230
1.0134
1.0468
1.0467
2.0816
1.0633
1.0575
1.0349
1.0582
6.0858
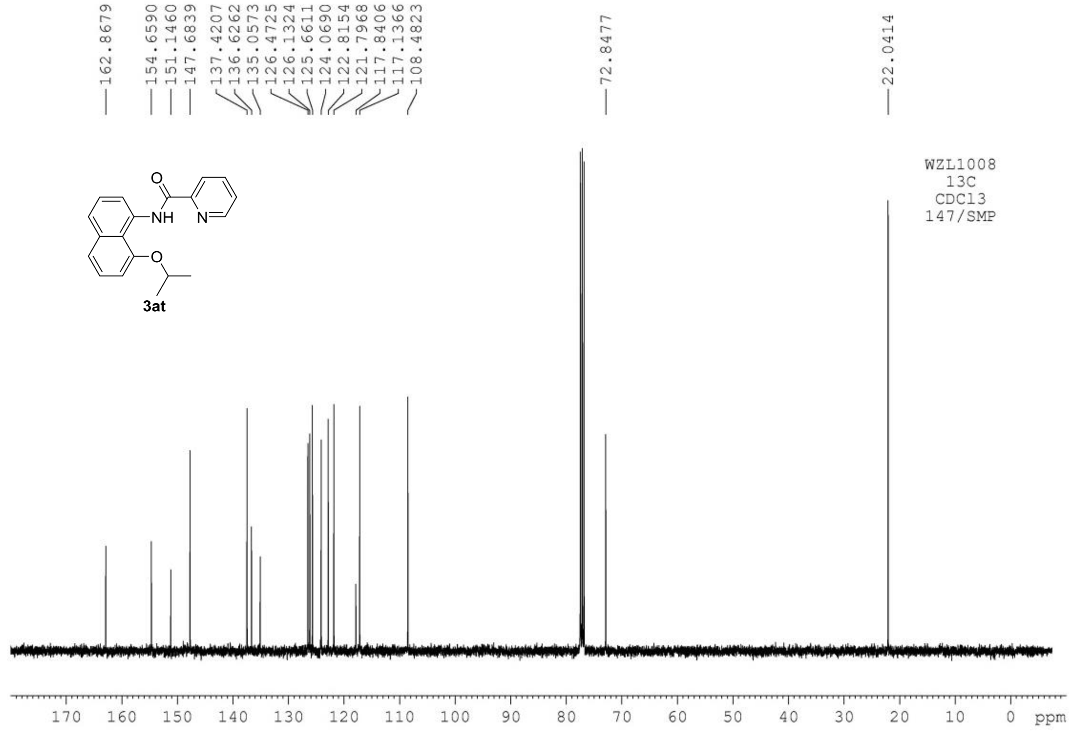
162.8679
154.6590
151.1460
147.6839
137.4207
136.6262
135.0573
126.4725
126.1324
125.6611
124.0690
122.8154
121.7968
117.8406
117.1366
108.4823
72.8477
22.0414
WZL1008
13C
CDCl3
147/SMP
O
NH
N
O
3at
170 160 150 140 130 120 110 100 90 80 70 60 50 40 30 20 10 0 ppm

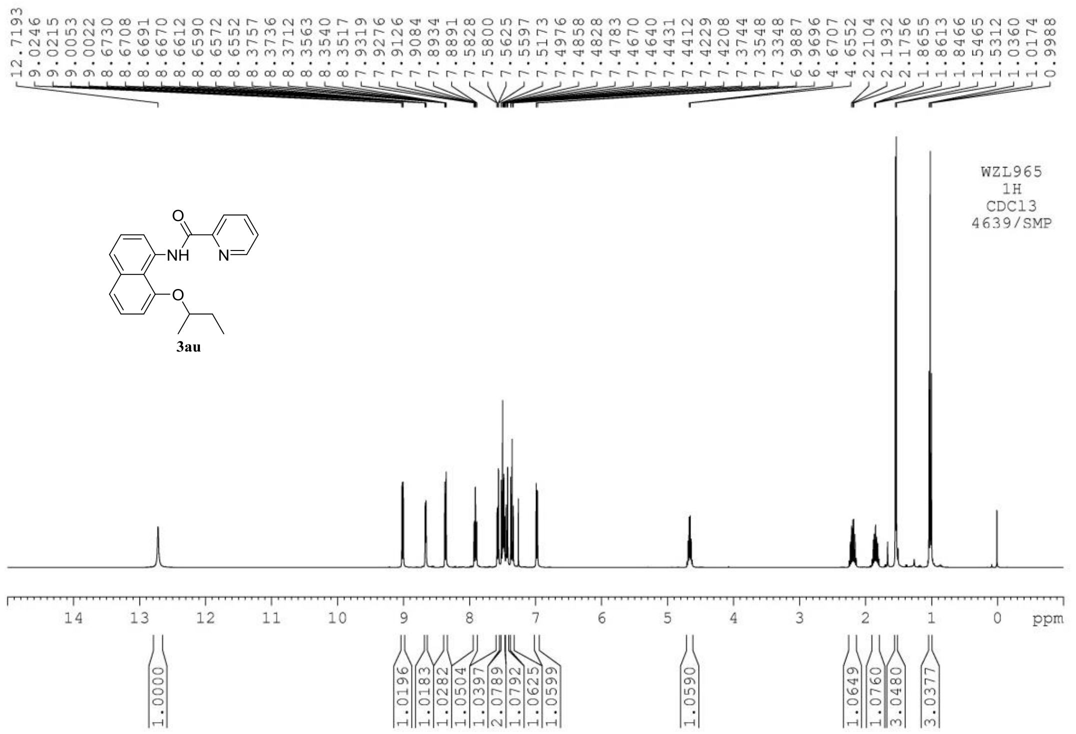
12.7193
9.0246
9.0215
9.0053
9.0022
8.6730
8.6708
8.6691
8.6670
8.6612
8.6590
8.6572
8.6552
8.3757
8.3736
8.3712
8.3563
8.3540
8.3517
7.9319
7.9276
7.9126
7.9084
7.8934
7.8891
7.5828
7.5800
7.5625
7.5597
7.5173
7.4976
7.4858
7.4828
7.4783
7.4670
7.4640
7.4431
7.4412
7.4229
7.4208
7.3744
7.3548
7.3348
6.9887
6.9696
4.6707
4.6552
2.2104
2.1932
2.1756
1.8655
1.8613
1.8466
1.5465
1.5312
1.0360
1.0174
0.9988
O
NH
N
O
3au
WZL965
1H
CDCl3
4639/SMP
ppm
1.0000
1.0196
1.0183
1.0282
1.0504
1.0397
2.0789
1.0792
1.0625
1.0599
1.0590
1.0649
1.0760
3.0480
3.0377

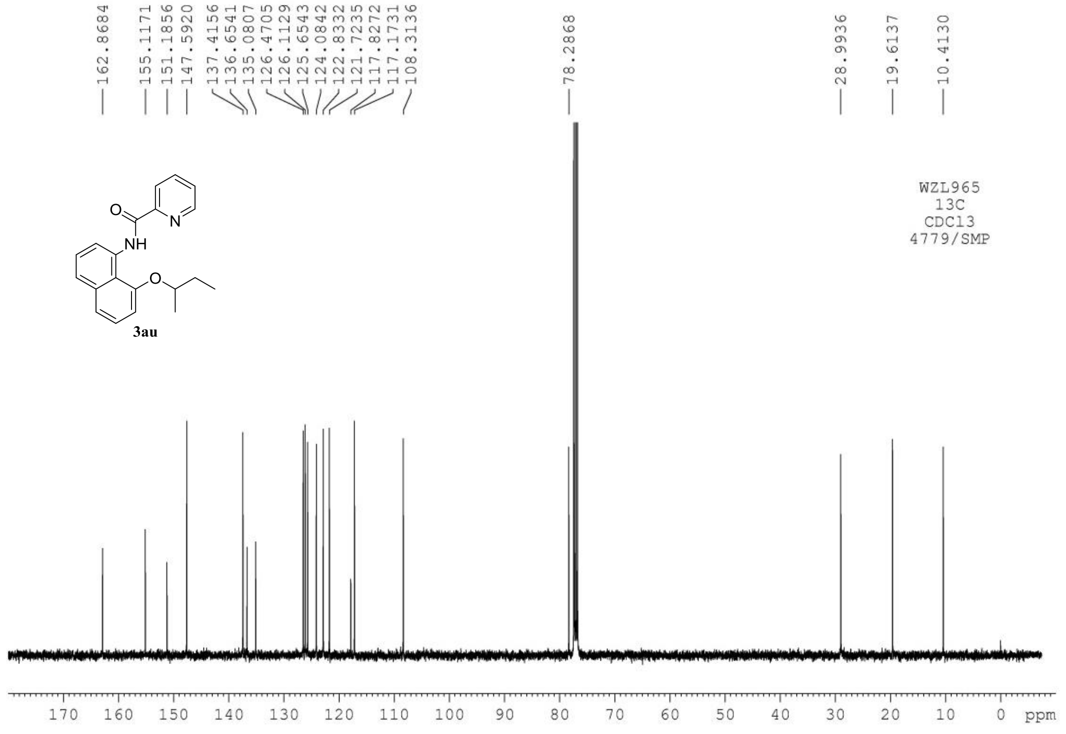
162.8684
155.1171
151.1856
147.5920
137.4156
136.6541
135.0807
126.4705
126.1129
125.6543
124.0842
122.8332
121.7235
117.8272
117.1731
108.3136
78.2868
28.9936
19.6137
10.4130
O
N
NH
O
3au
WZL965
13C
CDCl3
4779/SMP
ppm

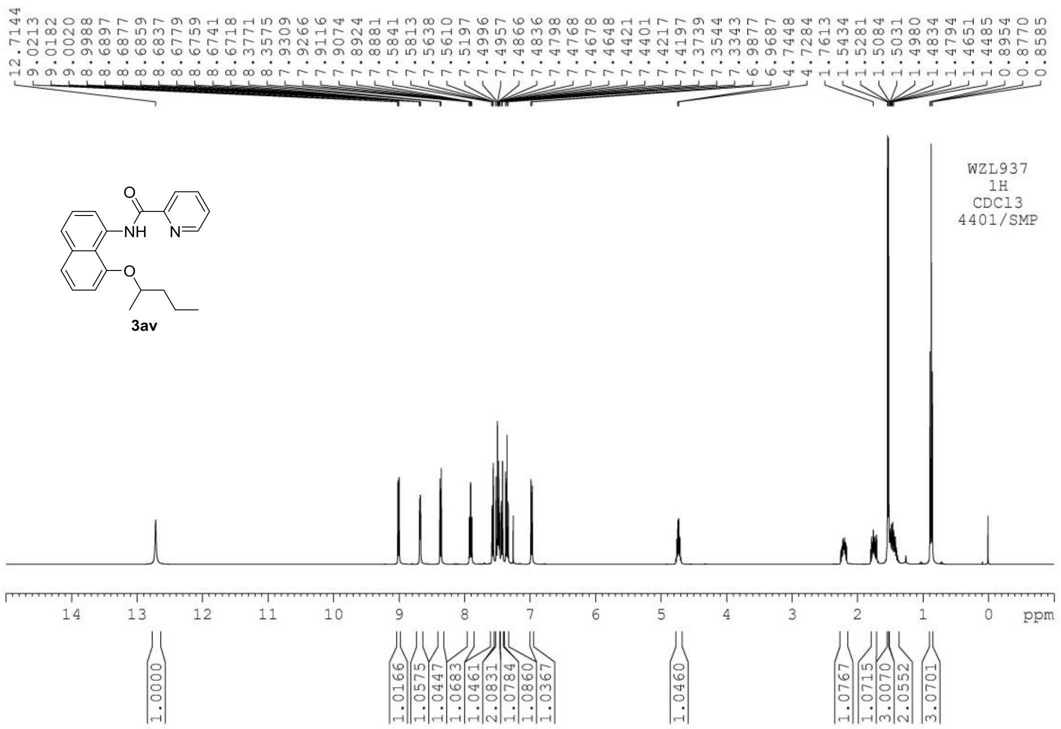
12.7144
9.0213
9.0182
9.0020
8.9988
8.6897
8.6877
8.6859
8.6837
8.6779
8.6759
8.6741
8.6718
8.3771
8.3575
7.9309
7.9266
7.9116
7.9074
7.8924
7.8881
7.5841
7.5813
7.5638
7.5610
7.5197
7.4996
7.4957
7.4866
7.4836
7.4798
7.4768
7.4678
7.4648
7.4421
7.4401
7.4217
7.4197
7.3739
7.3544
7.3343
6.9877
6.9687
4.7448
4.7284
1.7613
1.5434
1.5281
1.5084
1.5031
1.4980
1.4834
1.4794
1.4651
1.4485
0.8954
0.8770
0.8585
O
NH
N
O
3av
WZL937
1H
CDCl3
4401/SMP
14 13 12 11 10 9 8 7 6 5 4 3 2 1 0 ppm
1.0000
1.0166
1.0575
1.0447
1.0683
1.0461
2.0831
1.0784
1.0860
1.0367
1.0460
1.0767
1.0715
3.0070
2.0552
3.0701

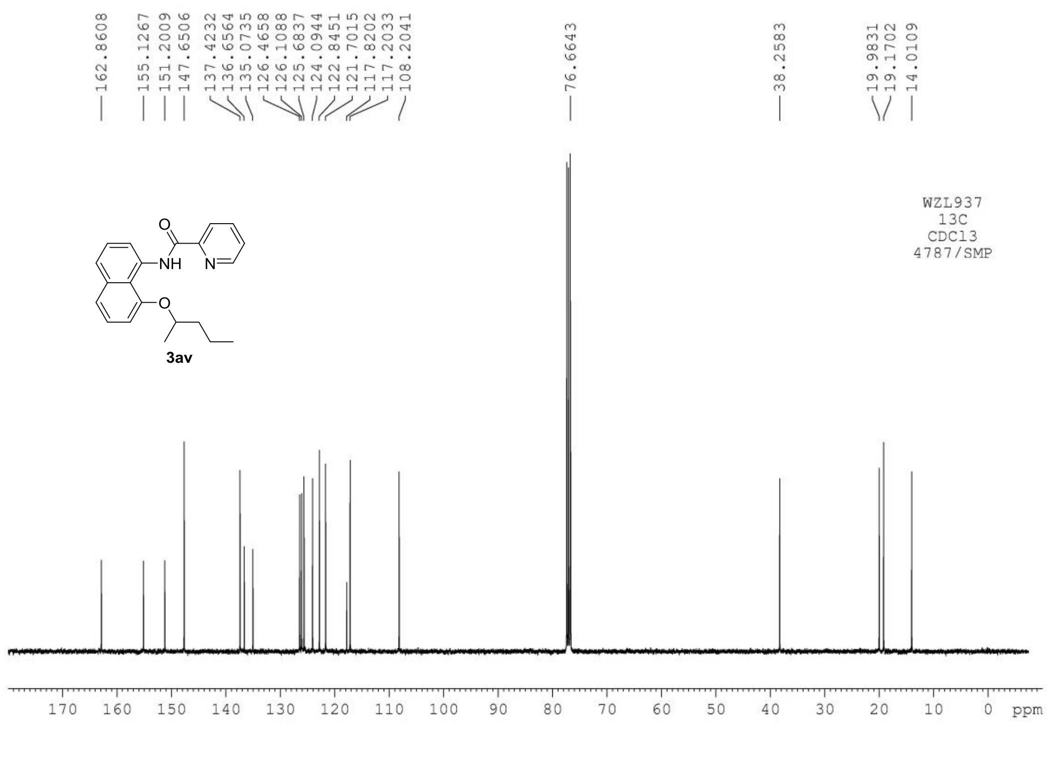
162.8608
155.1267
151.2009
147.6506
137.4232
136.6564
135.0735
126.4658
126.1088
125.6837
124.0944
122.8451
121.7015
117.8202
117.2033
108.2041
76.6643
38.2583
19.9831
19.1702
14.0109
O
NH
N
O
3av
WZL937
13C
CDCl3
4787/SMP
170 160 150 140 130 120 110 100 90 80 70 60 50 40 30 20 10 0 ppm
